# Exceptionally high brightness and long lifetime of efficient blue OLEDs for programmable active-matrix display

**DOI:** 10.1038/s41377-025-01817-x

**Published:** 2025-04-09

**Authors:** Chengcheng Wu, Kai-Ning Tong, Kefei Shi, Wei He, Manli Huang, Jie Yan, Siqi Li, Zhaoyun Jin, Xin Wang, Sinyeong Jung, Jingrui Ma, Yixi Zhuang, Rong-Jun Xie, Cunjiang Yu, Hong Meng, Xiao Wei Sun, Chuluo Yang, Yun Chi, Feiyu Kang, Guodan Wei

**Affiliations:** 1 Institute of Materials Research, Tsinghua Shenzhen International Graduate School (SIGS), Tsinghua University, Shenzhen, 518055 China; 2 Shenzhen Key Laboratory of Polymer Science and Technology, College of Materials Science and Engineering, Shenzhen University, Shenzhen, 518060 China; 3 Department of Materials Science and Engineering, Department of Chemistry, Center of Super-Diamond and Advanced Films (COSDAF), City University of Hong Kong, Kowloon Tong, Hong Kong SAR 999077 China; 4 Institute of Nanoscience and Applications, Department of Electrical and Electronic Engineering, Southern University of Science and Technology, Shenzhen, Guangdong 518055 China; 5 College of Materials and Fujian Key Laboratory of Surface andInterface Engineering for High Performance Materials, Xiamen University, Xiamen, 361005 China; 6 Department of Biomedical Engineering, Pennsylvania State University, University Park, PA 16802 USA; 7 Department of Engineering Science and Mechanics, Department of Materials Science and Engineering, Materials Research Institute, Pennsylvania State University, University Park, PA 16802 USA; 8 School of Advanced Materials, Peking University Shenzhen Graduated School, Peking University, Shenzhen, 518055 China

**Keywords:** Organic LEDs, Polymers

## Abstract

Blue phosphorescent OLEDs (Ph-OLEDs) have long faced critical challenges in efficiency, stability and brightness, which are crucial for advanced display. Herein, we introduce two novel Ir(III) emitters featuring a 3,6-di(*tert*-butyl)-9H-carbazolyl (tBuCz) substituted tridentate carbene pincer ligand, significantly improving efficiency and stability. The **tBuCz-*m*-CF3** and **tBuCz-*p*-CF3** complexes are designed to enhance steric encumbrance and minimize exciton accumulation. These innovations lead to exceptional photoluminescence quantum yields (PLQY) of 98% and an impressive decay rate constant of 7.97 × 10^5^ s^−1^ in doped thin films. The Ph-OLEDs emit blue light with a peak wavelength of 485 nm and CIE coordinates of (0.175, 0.446), exhibiting a peak external quantum efficiencies (EQE) of 31.62% and brightness up to 214,255 cd m^−2^. Notably, they shown minimal efficiency roll-off, retaining an EQE of 27.76% at 10,000 cd m^−2^, and 20.58% at 100,000 cd m^−2^. These consistent performances across various brightness levels represent a significant milestone for blue Ph-OLED technology. The devices also exhibit impressive stability, with an operational lifetime (LT_50_, the time taken for luminance to decrease by 50%) reaching 1237 h at 1000 cd m^−2^, setting new benchmarks for blue Ph-OLEDs. To enhance the color purity, hyper-OLEDs were developed with a full width at half maximum (FWHM) of 20 nm and the CIEy of 0.233, achieving an EQE_m_ of 29.78% and LT_50_ of 318 h at 1000 cd m^−2^. We also fabricated the active-matrix (AM) blue Hyper-OLEDs with 400 pixels per inch to demonstrate their application in AM displays.

## Abstract (web summary)

High-brightness, long-lifetime blue Ph-OLEDs were achieved by novel tBuCz-based asymmetric Ir(III) emitters.

## Graphical abstract

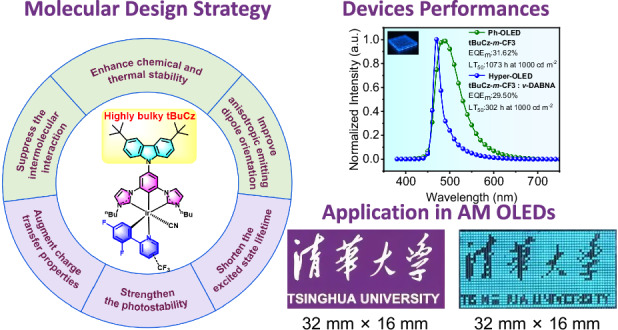

## Introduction

Organic light-emitting diodes (OLEDs) have been ubiquitous in various modern technologies such as smartphones, watches and televisions^1,2^ due to their advantages such as high resolution, high color quality, fast response time, energy efficiency and flexibility^3^. Nowadays, the development of stable and efficient blue phosphorescent OLEDs (Ph-OLEDs) with high brightness is critical for advancing augmented reality (AR) and virtual reality (VR) display technologies. These displays require high-resolution, high-brightness solutions to provide immersive and realistic visual experiences^4^. For high-resolution displays, high brightness is essential to ensure visibility and clarity of images, especially in AR applications where the display must compete with real-world lighting. Any deficiency in blue light emission can cause poor image quality and color distortion. Despite the success in making commercial red, yellow and green emitters^5,6^, blue Ph-OLEDs have traditionally faced challenges in terms of efficiency, stability, and brightness, making it essential to address these issues to meet the demands of next-generation high-end display applications^7^. The short lifetime of blue Ph-OLEDs can attribute to several key issues: (i) the higher blue photon energy compared with red or green counterparts; (ii) degradation of organic materials during continuous device operation; (iii) poor stability due to the high energies exerted to the excited molecules; (iv) inadequate carrier balance inside the compact recombination domains that caused severe efficiency roll-off^8^. The efficiency roll-off, in particular, is mainly due to triplet-triplet annihilation (TTA) and triplet-polaron annihilation (TPA), which are exacerbated by the long lifetime of triplet excitons (up to 100 μs) and their high concentration^9^.

The incorporation of heavy metal atoms in OLED emitters facilitates spin-orbit coupling (SOC), allowing fast singlet-triplet interconversion and ideal internal efficiency up to 100%^10^. Recent developments of triplet emitters, such as Au(III)^11^, Pt(II)^12^ and Pd(II)^13^ complexes, have shown significantly improved efficiency and stability. Other efforts to improve efficiencies can be done by increasing the thickness of light emitting layer^14–16^, and utilizing bipolar hosts^17^ and exciplex-forming co-hosts^18^. As for phosphorescent emitters, platinum(II) (Pt(II)) complexes have made significant advantages in color purity and device stability^19,20^. The “pure” blue-emitting PtON7-dtb demonstrated a high EQE of up to 24.8% with a CIE_y_ value of 0.078^21^. Furthermore, integrating the co-host system with PtON5-dbp-tBu (BD02), the Pt-based OLEDs exhibited remarkable device stability, achieving an operational lifetime of LT_70_ of 1113 h at a brightness level of 1000 cd m^−2^ ^14^. While Ph-OLEDs based on Ir(III) complexes have demonstrated high efficiency^15,22^, the challenge of efficiency roll-off at high brightness remains^23^. Numerous efforts have been explored to improve device efficiency and stability of Ir(III)-based blue Ph-OLEDs. For example, the stability could be effectively improved through deuteration^24^ and incorporation of pyrimidine-based ligands^25^. Through enhancing the intermolecular interactions via hydrogen bond and π–π interactions by incorporating the bulky trimethylsilyl (TMS) substituent on the C^N ligand, the blue OLED performance could be effectively enhanced to an EQE over 30%, while the LT_50_ remains only 5 h at the luminance of 200 cd m^−2^ ^26^. Sang Ook Kang et al. showed that the intramolecular hydrogen bond in an ancillary ligand from a heteroleptic Ir(III) complex could stabilize the blue phosphorescence for 34.3 h at an initial luminance of 400 cd m^−2^ ^27^. To further improve the device stability, device architecture optimization has been explored through the introduction of novel narrow-bandgap host material, named as DBF-DMS to reach a practical EQE up to 19.6% at 1000 cd m^−2^ and a LT_50_ up to 122 h^28^. Jun Yeob Lee et al. designed a series of hole transport type exciton blocking layer to improve the EQE to 24.8% and the LT_50_ of 140 h at 500 cd m^−2^, respectively^29^. Forrest et al. achieved effective management of hot excited states through gradient doping to enhance the devices stability, achieving a LT_80_ of 334 h at an initial luminance of 1000 cd m^−2^ ^30^. Most recently, they also demonstrated polariton-enhanced Purcel effects in deep blue Ph-OLEDs, resulting in LT_90_ up to 140 h^1^. Other strategies, such as controlling exciton-induced electron transfer^31^ and exciplex co-host system for efficient energy transfer^32^ have been actively introduced to improve device stability. However, high efficiency and high luminance usually cause serious reduction in device operation lifetimes. Therefore, critical challenges remain to develop intrinsic stable blue emitters with high efficiency and stability at high luminance for further success of blue Ph-OLEDs beyond conventional display and lighting application.

In this study, four novel blue Ir(III) based carbene pincer emitters, namely ***m*-CF3,** ***p*-CF3,** **tBuCz-*m*-CF3** and **tBuCz-*p*-CF3**, were rationally designed and synthesized. The strategic incorporation of tBuCz fragment on pincer chelate increases both the steric encumbrance and electron delocalization across the whole molecule. Hence, they exhibited emission peak wavelengths at 485 nm and photoluminescence quantum yield (Φ_PL_) between 86‒98%, high horizontal transition dipole orientation up to 93%, and fast radiative decay rate of 7.97 × 10^5 ^s^−1^. Ph-OLEDs based on **tBuCz-*m*-CF3** and **tBuCz-*p*-CF3** doped in an exciplex-forming co-host have achieved maximum EQE of 31.62% and 30.72%, respectively. Remarkably, their EQE values remained as high as 20.58% and 20.87% even at extremely high luminance of 100,000 cd m^−2^. As a result, these devices established new benchmarks in both the suppressed efficiency roll-off and high stability of sky-blue Ir(III) based Ph-OLEDs, reaching an unprecedented LT_50_ up to 1237 and 1073 h at 1000 cd m^−2^. To improve the color purity, the hyper-OLEDs has been conducted, in which their FWHM is down to 20 nm with the CIEy of 0.233. Such hyper-OLEDs also demonstrated high efficiency of EQEm of 29.78% and operational stability are over 300 h at 1000 cd m^−2^. The AM blue Hyper-OLEDs also have been fabricated with 400 pixels per inch to demonstrate their application in AM displays. Hence, this work sheds light on the rational design of new blue emitters employing iridium emitters to strengthen device stability for the next-generation display and lighting applications.

## Results

### Molecular design and theoretical analysis

**Scheme 1:**
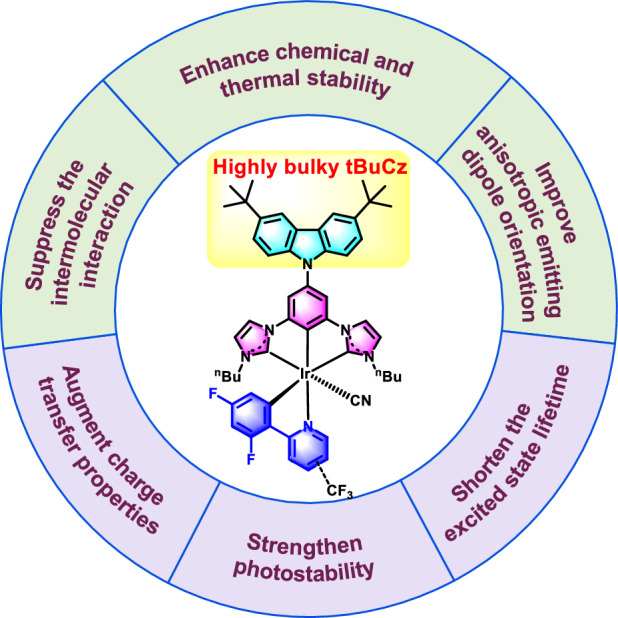
The molecular design concept of asymmetric Ir(III) complexes

**Fig. 1:**
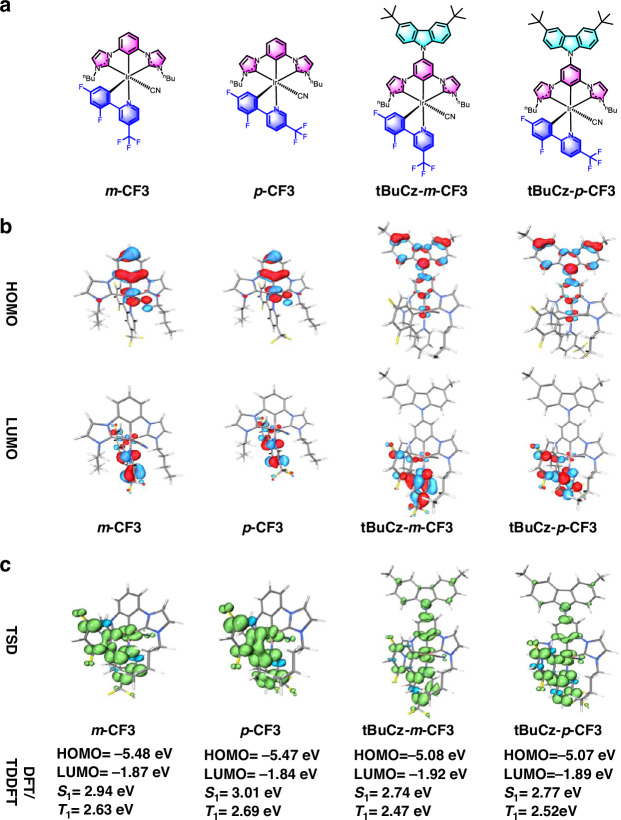
Essential characteristics of Ir(III) emitters *m*-CF3, *p*-CF3, tBuCz-*m*-CF3 and tBuCz-*p*-CF3. **a** Chemical structures; **b** isosurface of frontier molecular orbitals; **c** isosurface of triplet-state spin density (TSD)

Most of the neutral Ir(III) complexes are designed as the [2 + 2 + 2]- and [3 + 3]-types, featuring an iridium metal center configuration that provides rigidity and durability in OLED application^5^. Recently, [3 + 2 + 1] coordinated asymmetrical Ir (III) complexes have demonstrated innovative molecular configurations, incorporating a tridentate ligand bearing an N-heterocyclic carbene (NHC) ring to enhance stability and phosphorescent efficiency, a bidentate ligand to fine-tune the emission color, and a mono-dentate ligand to neutralize the compounds^33^. The tridentate ligand such as bicarbene pincer ligand 1,1′-(1,3-phenylene)bis(3-butyl-1H-imidazol-3-ium) dibromide (pbib) has been employed to raise the energy level of the triplet metal-centered state (^3^MC) states while stabilizing the filled dπ orbital and strengthening the bond dissociation energy^34^. Herein, a novel 3,6-di(tert-butyl)-9H-carbazolyl (tBuCz) fragment was introduced to the regular dicarbene pincer ligand to obtain the functionalized pincer ligand of tBuCz-pbib, which could effectively suppress intermolecular interactions in the solid state^20^. Specifically, the introduction of the bulky tBuCz- group to enhance anisotropic emitting dipole orientation, has proven to be an effective strategy to improve their electroluminescence^33^. Therefore, the tBuCz-pbib ligand, bearing highly bulky substituents on the tBuCz unit, provides more advantages than the regular pbib ligand in terms of :(i) enhanced chemical and thermal stability; (ii) improved photostability and electrochemical stability; (iii) suppressed intermolecular interactions and improved anisotropic emitting dipole orientation. The role and effect of this unique molecular design concept of asymmetric Ir(III) complexes with bulky tBuCz- are illustrated in Scheme 1. The molecular structures of four Ir(III) emitters ***m*-CF3,** ***p*-CF3,** **tBuCz-*m*-CF3** and **tBuCz-*p*-CF3** were depicted in Fig. 1a, to which the synthetic details are described in the supplementary materials in Fig. S1. The bidentate (C^N) ligands are prepared following the literature procedures^35^, and the targeted Ir(III) emitters are obtained using an established method^36–38^. Both **tBuCz-*m*-CF3** and **tBuCz-*p*-CF3** exhibited superb thermal stability (see Fig. S2 and Table S1), as evidenced by their high decomposition temperature (T_d_) of 416 °C and 412 °C, which are higher than that of ***m*-CF3** (374 °C) and ***p*-CF3** (378 °C), indicating an enhanced thermal stability resulting from the tBuCz moiety. Moreover, the glass transition temperature (T_g_) of **tBuCz-*p*-CF3** reaches 217 °C, higher than that of ***m*-CF3** (144 °C) and ***p*-CF3** (166 °C), which is beneficial for fabrication of vacuum-deposited OLEDs due to the higher thermostability. Cyclic voltammetry (CV) was conducted for exploration of their electrochemical property. As shown in Fig. S3a, both **tBuCz-*m*-CF3** and **tBuCz-*p*-CF3** illustrated a reversible oxidation process, which is in sharp contrast to a quasi-reversible oxidation that observed for ***m*-CF3** and ***p*-CF3**. Additionally, their HOMO energy levels are determined from the onset of oxidation peaks, while the LUMO energy levels are derived from the difference of HOMO and optical energy gap (*E*_g_), where *E*_g_ is calculated from the onset of UV-vis absorption spectra^39^. The measured HOMO levels of ***m*-CF3** and ***p*-CF3** are calculated to be ‒5.55 eV and ‒5.52 eV, respectively, which are more stabilized than that of **tBuCz-*m*-CF3** (‒5.36 eV) and **tBuCz-*p*-CF3** (‒5.36 eV), as shown in Fig. S3b. Alternatively, their LUMO energy levels are located at approx. ‒2.90 eV. Hence, the introduction of tBuCz moiety on the pbib ligand has only affected their HOMO energy level. Meanwhile, a multi-scan experiment is carried out, to which **tBuCz-*m*-CF3** showed good electrochemical stability, while ***m*-CF3** exhibited a gradually decrease in redox current (Fig. S3c, d)^40^.

Density functional theory (DFT) calculations were performed using the Gaussian 16 software package with the B3LYP functional and LANL2DZ basis set to investigate the electronic structures, optimized ground-state geometries, and molecular orbital configurations of the Ir(III) complexes, as detailed in Table 1, Fig. S4 and Table S2. For both **tBuCz-*m*-CF3** and **tBuCz-*p*-CF3**, the dihedral angle between the tBuCz moiety and the dicarbene pincer ligand are 55.64° and 56.31°, respectively. as illustrated in Fig. S5a. Additionally, the *meta*-substituted CF_3_ group on the C^N ligand showed a stronger electron withdrawing capacity than that of *para*-substituted CF_3_ group. This is evidenced by the lower electrostatic potential (ESP) near *p*-substituted CF_3_ group, as depicted in Fig. S5b. In addition, for the four complexes, the HOMO was mainly localized on the dicarbene pincer ligand, while the LUMO was predominantly residing on C^N ligand, as depicted in Fig. 1b. Upon introduction of the tBuCz substituent, while the LUMO of ***m*-CF3,** ***p*-CF3,** **tBuCz-*m*-CF3** and **tBuCz-*p*-CF3** remained similar, the HOMO is now dominated by the tBuCz group. All trends noted above align well with those observed in their electrochemical analysis.

**Table 1:** Electrochemical properties and DFT/TD-DFT results of the studied Ir(III) emitters

Complex	Experimental				DFT/TD-DFT Calculation				
	E_1/2_^ox^ (V)	HOMO (eV)	LUMO (eV)	E_g_ (eV)	HOMO (eV)	LUMO (eV)	H → L (eV)	Major Contribution (s_0_ → s_1_)	Oscilator Strength
*m*-CF3	+1.19	−5.55	−2.87	2.68	−5.48	−1.87	3.61	H → L 98.4%	0.0001
*p*-CF3	+1.15	−5.52	−2.94	2.58	−5.47	−1.84	3.63	H → L 98.5%	0.0001
tBuCz-*m*-CF3	+0.97	−5.36	−2.86	2.50	−5.08	−1.92	3.16	H → L 91.0% H−3 → L 8.6%	0.0007
tBuCz-*p*-CF3	+0.96	−5.36	−2.91	2.48	−5.07	−1.89	3.18	H → L 91.6% H−3 → L 8.1%	0.0005

Time dependent DFT (TD-DFT) calculation was employed to simulate their lowest singlet (S_1_) and lowest triplet (T_1_) excited state energy, the details are summarized in Figs. S6–S13 and Tables S3–S8. As listed in Fig. 1c, the computed T_1_ energies are 2.63 eV, 2.69 eV, 2.47 eV and 2.52 eV for ***m*-CF3,** ***p*-CF3,** **tBuCz-*m*-CF3** and **tBuCz-*p*-CF3**, respectively, corresponding to the calculated emission wavelength of 471 nm, 460 nm, 502 nm and 492 nm. This indicates that the incorporated strong electron-donating group of tBuCz has a redshift perturbation on emission peak, which aligns well with those observed in toluene at room temperature (RT), as listed in Table 2. As for the triplet spin density (TSD) simulation, the TSD of ***m*-CF3** and ***p*-CF3** revealed the strong association from the bidentate ligand and Ir(III) metal center, but with a minimal contribution from the cyano (CN) group. Besides, the introduction of the tBuCz fragment, as shown in **tBuCz-*m*-CF3** and **tBuCz-*p*-CF3**, expanded the exciton distribution from the bidentate C^N ligand and Ir(III) metal to encompass the entired molecule, as illustrated in Fig. 1c. Hence, the tBuCz group could not only increase the steric encumbrance, but also facilitate the exciton delocalization over the entired iridium complex, enhancing exciton distribution and energy transfer^41^.

**Table 2:** Summary of photophysical properties of the studied Ir(III) emitters ***m*-CF3,** ***p*-CF3,** **tBuCz-*m*-CF3** and **tBuCz-*p*-CF3**

Complex	298 K in solution ^a^				78 K in solution ^b^	298 K in thin film ^c^					
	Absorp. (nm) (10^−4 ^M^−1^ cm^−1^)	λ (nm)	τ (μs)	Φ (%)	λ (nm)	λ (nm)	τ (μs)	Φ (%)	k_*r*_ (10^5^ s^−1^)	k_*nr*_ (10^5^ s^−1^)	Θ_//_
*m*-CF3	316 (1.81); 344 (0.94)	466/489	3.16	82	463/489	488	2.05	86	4.19	0.68	85
*p*-CF3	315 (1.74); 344 (0.68)	480	2.28	85	480	480	1.75	88	5.02	0.69	83
tBuCz-*m*-CF3	344 (1.15); 347 (1.05)	483	3.63	78	496	484	1.44	98	6.81	0.14	93
tBuCz-*p*-CF3	334 (1.26); 346 (1.16)	488	3.30	79	499	488	1.23	98	7.97	0.16	90

^a^Recorded in degassed toluene at room temperature (2 ×10^−5 ^M)

^b^Recorded in degassed toluene at 78 K

^c^Recorded in mixed host (SiCzCz: SiTrzCz2)

### Photophysical properties

**Fig. 2:**
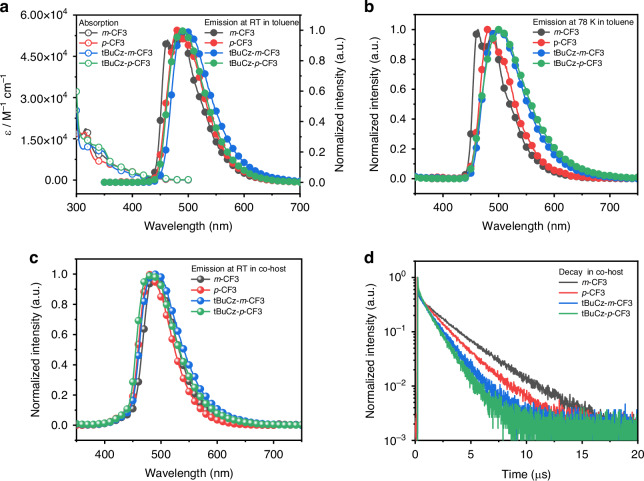
Photophysical properties of the studied Ir(III) emitters. **a** Absorption and emission spectra in toluene (2 × 10^−5 ^M) at room temperature (RT). **b** Emission spectra obtained in toluene solution at 78 K. **c** Emission spectra recorded in co-host (SiCzCz and SiTrzCz2). **d** PL decay curves in co-host (SiCzCz and SiTrzCz2)

The UV-visible absorption and emission spectra of ***m*-CF3,** ***p*-CF3,** **tBuCz-*m*-CF3** and **tBuCz-*p*-CF3** in toluene at 298 K are illustrated in Fig. 2a, and their numerical data are presented in Table 2. All complexes exhibit intense absorption bands before 300 nm with extinction coefficients (ε) on the order of 10^4 ^M^−1^ cm^−1^, which can be attributed to the ligand-centered (LC) transition originating from the N-heterocyclic carbenes (NHC) pincer and C^N ligands^42^. The moderately intense absorption band from 310 nm to 330 nm are assigned as a mixed intra-ligand (IL) [π → π*(C^N)] and ligand-to-ligand charge transfer (LLCT) [π → π*(C^C^C → C^N)] transitions. The absorption tails beyond 350 nm, characterized by extinction coefficient of 10^3 ^M^−1^ cm^−1^, are ascribed to the admixture of triplet ligand-to-ligand and metal-to-ligand charge transfer (^3^LLCT and ^3^MLCT) transitions^35^. Upon excitation at 350 nm in degassed toluene at 298 K, ***m*-CF3** exhibited a structured emission band with dual peak max. at 463 nm and 489 nm. In contrast, ***p*-CF3** displayed a structureless emission profile with single peak max. at 480 nm. For **tBuCz-*m*-CF3** and **tBuCz-*p*-CF3**, structureless emission was observed with peak max. at 483 nm and 488 nm, respectively, where the red-shifted peak wavelength could be attributed to the incorporation of tBuCz group. The phosphorescence of ***m*-CF3** and ***p*-CF3** are predominantly originated from the metal-perturbed ^3^LLCT[π → π*(C^C^C → C^N] transition; i.e., from pbib to C^N ligand^35,42^. Moreover, the emission spectra of **tBuCz-*m*-CF3** and **tBuCz-*p*-CF3** at 78 K were also recorded (Fig. 2b and Table 2), showing a red-shifted emission profile in reference to that of ***m*-CF3** and ***p*-CF3**, indicating the remarkable influence imposed by the tBuCz substituent. To further investigate the effect of the introduction of the tBuCz segment on the intrinsic photostability of the Ir(III) complexes, a PL stability test was conducted on the complexes under continuous irradiation to multi-scanning to 999 cycles with a CW ozone-free xenon arc lamp at room temperature, as shown in Fig. S14. To note, the emission intensity decreased over time, with reductions of 22.22% for **tBuCz-*m*-CF3** and 19.05% for **tBuCz-*p*-CF3**. These values are significantly lower than the reductions observed for the complexes without the tBuCz segment, which exhibited intensity reductions of 34.37% for ***m*-CF3** and 35.89% for ***p*-CF3**. This suggests that the incorporation of the tBuCz segment enhances the photostability of the Ir(III) complexes. These trends are consistent with the device stability results presented in the subsequent section on electroluminescence (EL) performance.

All the studied Ir(III) complexes revealed the structureless emission characteristics at 298 K in polymethylmethacrylate (PMMA) matrices (2 weight-% dopant level, Table S9, Fig. S15), in which ***m*-CF3** and **tBuCz-*m*-CF3** shown the comparable peak emission compared with their PL behavior in toluene at RT, and ***p*-CF3** and **tBuCz-*p*-CF3** depicted a somewhat red-shift emission. With the exception of ***m*-CF3**, the excited state lifetimes of all the complexes are reduced, and there is a corresponding increase in PLQY, which could be contributed to the microenvironment changed, such as the polarity and arrangement mode etc., affect the non-radiative decay processes of the emitter, influencing its excited-state lifetime and PLQY. The emission behaviors of ***m*-CF3,** ***p*-CF3,** **tBuCz-*m*-CF3** and **tBuCz-*p*-CF3** in co-host (60 wt% of SiCzCz, 35 wt% of SiTrzCz2, and 5 wt% of these emitters) were investigated and the results are depicted in Fig. 2c and Table 2. In general, all these samples have displayed structureless sky-blue emission between 480 ‒ 488 nm and with PLQY closer to unity. The different behavior of PLQY in solution vs films also has been observation, in which ***m*-CF3** and ***p*-CF3** shown a higherPLQY compared to those complexes bearing tBuCz unit in solution, which could be attributed to the introduction of tBuCz to increase their rotation motion of tBuCz in dispersed solution. Yet now, **tBuCz-*m*-CF3** and **tBuCz-*p*-CF3** depicted a higher PLQY in solid state, which could be due to the bulky tBuCz group introduces significant steric hindrance, reducing the likelihood of intermolecular interactions that can lead to non-radiative decay. Notably, in thin films, both ***m*-CF3** and ***p*-CF3** exhibited a notable red shifting of emission in comparison to the spectra recorded in toluene, while those of **tBuCz-*m*-CF3** and **tBuCz-*p*-CF3** remained essentially unaltered with high color stability. Moreover, the PLQY of **tBuCz-*m*-CF3** and **tBuCz-*p*-CF3** featuring the tBuCz moiety have both reached 98%, which is higher than that of ***m*-CF3** (86%) and ***p*-CF3** (88%). This improvement could be owing to the greater steric encumbrance introduced by the tBuCz unit and the suppressed TTA and TPA processes^43^. Finally, all complexes displayed observed radiation lifetimes in the range of 1.23 – 2.05 µs (see Table 2 and Fig. 2d), yielding radiative decay rate constant (k_*r*_) as fast as 7.97 × 10^5 ^s^−1^(Table 2).

### Electroluminescence

To evaluate the EL performances of the obtained Ir(III) complexes, OLED devices were fabricated by vacuum deposition. The device configurations consisted of indium tin oxide (ITO, 185 nm) / dipyrazino[2,3-f:2′,3′-h]quinoxaline-2,3,6,7,10,11-hexacarbonitrile (HAT-CN, 10 nm) / N-(biphenyl-4-yl)-9,9-dimethyl-N-(4-(9-phenyl-9H-carbazol-3-yl)phenyl)-9H-fluoren-2-amine (BCFN, 60 nm) / 9-(3-(triphenylsilyl)phenyl)-9H-3,9’-bicarbazole (SiCzCz, 5 nm) / the emitting layer (EML) / 2-phenyl-4,6-bis(3-(triphenylsilyl)phenyl)-1,3,5-triazine (mSiTrz, 5 nm) / mSiTrz: Liq (31 nm, 50 wt% : 50 wt%) / 8-hydroxyquinoline lithium (Liq, 2 nm) / aluminum (Al, 120 nm), in which EML consisted of approx. 2:1 ratio of SiCzCz and SiTrzCz2 plus x wt% of Ir(III) emitters. The co-host system, comprising SiCzCz and SiTrzCz2, has been previously reported to exhibit a fast reverse intersystem crossing (RISC) process, which not only enhance the overall efficiency but also contribute significantly to the stability of the devices by facilitating efficient energy transfer and reducing the likelihood of triplet-triplet annihilation, resulting in low efficiency roll-off^14^. The dopant concentration was tuned over the range of 3 ‒ 9 wt%, among which the optimized dopant ratio is 5 wt%, as shown by the highest EQE and lowest efficiency roll-off for the representative emitter **tBuCz-*m*-CF3**, see Fig. S16a–c. The optimized EML thickness was next confirmed to be 35 nm. These performance characteristics were depicted in Fig. S16d–f, while their numerical data is summarized in Table S10.

**Fig. 3:**
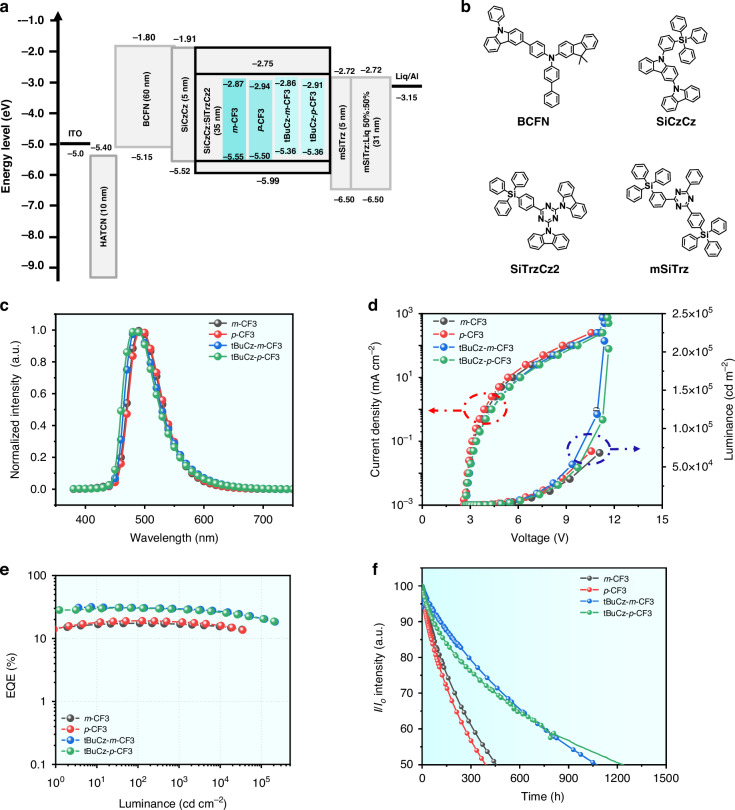
The electroluminescence performance of the studied Ir(III) emitters. **a** Device architecture and energy diagrams. **b** Chemical structures of the employed functional layers. **c** EL spectra. **d** J-V-L curves. **e** EQE versus luminance curves. **f** Operational lifetimes of fabricated devices with an initial luminescence of 1000 cd m^−2^

The aforementioned device architecture was then applied in the fabrication of a complete set of OLED devices. Their energy level diagram and chemical structures of the employed materials were illustrated in Fig. 3a, b. The representative EL spectra are measured at the current density (*J*) of 10 mA cm^−2^, exhibiting a structureless peak at *ca*. 485 nm, as shown in Fig. 3c. The current density-voltage-luminance (*J-V-L*) curves of devices featuring ***m*-CF3,** ***p*-CF3,** **tBuCz-*m*-CF3** and **tBuCz-*p*-CF3** are provided in Fig. 3d, in which their turn-on voltages span the narrowed range of 2.7 ‒ 2.8 V. Also, max. luminance of 214,255 cd m^−2^ and 205,301 cd m^−2^ and small efficiency roll-off were observed for **tBuCz-*m*-CF3** and **tBuCz-*p*-CF3**, respectively. To the best of our knowledge, these results are among the best device performance ever reported for known Ir(III) phosphors, as illustrated in Table S11. On the contrary, the lower max. luminance of 71,190 and 68,180 cd m^−2^ were recorded for devices based on the dopants ***m*-CF3** and ***p*-CF3**. Figure 3e showed the EQE versus the luminance diagram, to which the dopants of **tBuCz-*m*-CF3** and **tBuCz-*p*-CF3** provided excellent max. EQE up to 31.62% and 30.72%, respectively, while the corresponding CE_m_ and PE_m_ are 73.82 cd A^−1^ and 78.81 lm W^−1^ for **tBuCz-*m*-CF3**, and 67.17 cd A^−1^ and 65.95 lm W^−1^ for **tBuCz-*p*-CF3**. Impressively, the EQE values of 20.58% and 20.87% are observed for **tBuCz-*m*-CF3** and **tBuCz-*p*-CF3** at the luminance of 100,000 cd m^−2^. Therefore, the tBuCz fragment has successfully reduced the exciton quenching and accumulation through efficient exciton distribution. Such a result serves as a testimony of our ongoing design in achieving efficient performances. Finally, stability of the Ph-OLEDs was evaluated by assessing the operational lifetime LT_50_ at a current density (*J*) of 10 mA cm^−2^. The LT_50_ at a brightness of 1000 cd m^−2^ can be extrapolated using the equation:

$${L}_{0}^{n}\times {{\rm{LT}}}_{50}^{10\,{\rm{mA}}\; {\rm{c}}{{\rm{m}}}^{-2}}={L}^{n}\times {{\rm{LT}}}_{50}^{1000\,{\rm{cd}}\,{{\rm{m}}}^{-2}}$$

where (*L*_0_) is the initial brightness recorded at 10 mA cm^−2^ and *n* refers to an acceleration factor. Despite the high measured value of n based on **tBuCz-*m*-CF3** at 1.95 as depicted in Fig. S17, it generally falls within the range of 1.5–1.8. Therefore, we have chosen to use a conservative estimate of 1.8. The Ph-OLEDs utilizing **tBuCz-*m*-CF3** and **tBuCz-*p*-CF3** demonstrated LT_50_ of 1073 and 1237 h at 1000 cd m^−2^, respectively, as illustrated in Fig. 3f and Table 3. Conversely, devices employing ***m*-CF3** and ***p*-CF3** as emitters exhibited relatively inferior LT_50_ of 452 and 390 h, respectively. On the one hand, the fast radiative process of **tBuCz-*m*-CF3** and **tBuCz-*p*-CF3** is as high as 6.81 × 10^5^ s^−1^ and 7.97 × 10^5^ s^−1^, which is advantageous for the concomitant improvement of the operational lifetime of their devices since it greatly contributes to the reduction of triplet exction concentration accumuation. Such a low triplet population at high brightness, on the other hand, could significantly suppress TTA and TPA processes, resulting in suppressed efficiency roll-off. As a result, the EQE based on **tBuCz-*m*-CF3** and **tBuCz-*p*-CF3** could sustain remarkably high values of 28.01% and 26.20%, respectively, even at an elevated luminance of 10,000 cd m^−2^. (Table 3 and Fig. 3e). Therefore, the greatly enhanced device lifetime with introduced **tBuCz-*m*-CF3** and **tBuCz-*p*-CF3** as emitter is consistent with the suppressed efficiency roll-off^44^. These results also validate both the high efficiency and stable lifetime of our Ph-OLEDs based on **tBuCz-*m*-CF3** and **tBuCz-*p*-CF3**, making them highly desirable for future commercial applications.

**Table 3:** EL performances of the blue emitting OLEDs based on the studied Ir(III) emitters

Dopant	V_on_^a^	EL Peak ^b^	EQE (%) ^c^				CE ^c^	PE ^c^	CIE ^d^	L_max_^e^	FWHM ^f^	LT_50_^g^
	(V)	(nm)	Max.	10000 (cd m^−2^)	50000 (cd m^−2^)	100000 (cd m^−2^)	(cd A^−1^)	(lm W^−1^)	(x,y)	(cd m^−2^)	(nm)	1000 (cd m^−2^)
*m*-CF3	2.80	492	19.05	16.00	8.40	/	50.92	52.73	0.179,0.498	71190	63	452
*p*-CF3	2.73	496	17.40	15.51	9.69	/	44.28	45.30	0.167,0.463	68180	62	390
tBuCz-*m*-CF3	2.77	485	31.62	27.76	22.74	20.58	73.82	78.81	0.175,0.446	214255	65	1073
tBuCz-*p*-CF3	2.73	485	30.72	25.67	22.67	20.87	67.17	65.95	0.166,0.410	205301	66	1237
1% *v*-DABNA/ 5% tBuCz-*m*-CF3	2.75	472	29.50	22.08	16.03	15.08	42.20	40.30	0.141,0.233	105737	20	302
1% *v*-DABNA/ 5% tBuCz-*p*-CF3	2.80	472	29.78	20.34	13.72	12.04^h^	41.86	40.27	0.140,0.233	83474	21	318

^a^V_on_ is defined as the driving voltage at the brightness of 1 cd m^−2^

^b^Electroluminescent peak is recorded at current density of 10 mA cm^−2^

^c^External quantum efficiency(EQE), current efficiency(CE) and power eficiency(PE) are recorded at their maximum, 10,000 cd m^−2^, 50,000 cd m^−2^ and 100,000 cd m^−2^

^d^ClE coordinate is recorded at current density of 10 mA cm^−2^

^e^L_m_ is defined as the maximum luminance

^f^FWHM refers to the full width at half maximum

^g^The operational lifetime are from the initial brightness to half

^h^The value was measured at the max. brightness

**Fig. 4:**
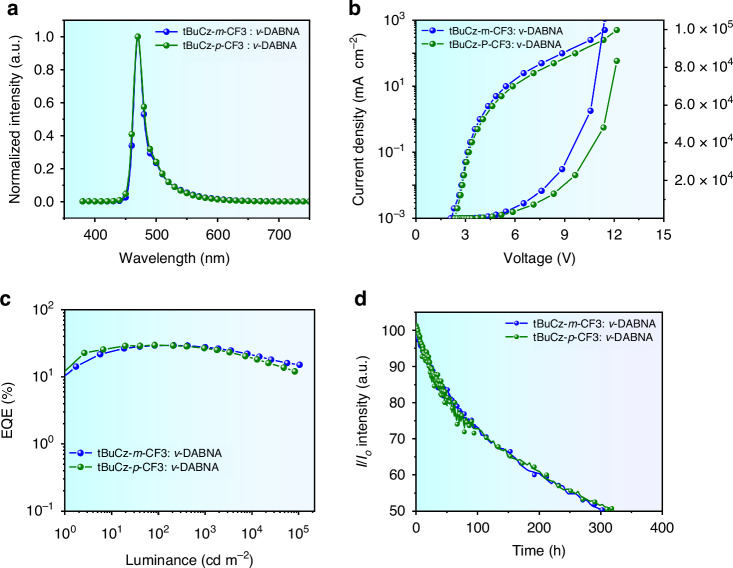
The electroluminescence performance of the studied Ir(III) emitters for Hyper-OLED. **a** EL spectra. **b** J-V-L curves. **c** EQE versus luminance curves. **d** Operational lifetimes of fabricated devices with an initial luminescence of 1000 cd m^−2^

To further unravel their EL performances, the emitting dipole orientation are recorded and the results are depicted in Fig. S18. As can be seen, **tBuCz-*m*-CF3 (Fig. 18(c))** and **tBuCz-*p*-CF3 (Fig. 18(d))** possess the horizontal transition dipole ratio (Θ_//_) of 93% and 90% in co-deposited SiCzCz and SiTrzCz2 films, which are slightly higher than that of ***m*-CF3** (85%) **(**Fig. 4a**)** and ***p*-CF3** (83%) **(**Fig. 4b**)** in the EML thickness of 35 nm. The higher emitting dipole orientation towards horizontal alignment improves the efficiency of light out-coupling, reduces non-radiative losses, and better aligns with the optical design of the OLED, leading to a higher EQE. The impact of enhanced EQE on outcoupling efficiency and EQE improvement has been quantified by modal power dissipation analysis, which was conducted utilizing the classical dipole model and transfer matrix method^45^. The optical constants of all materials utilized were ascertained through variable angle spectroscopic ellipsometry (VASE) and are displayed in Fig. S19. The outcoupling efficiency (*η*_out_), depicted as the air mode in Fig. S20a, was calculated for different configurations: 0.276 for ***m*-CF3**, 0.261 for ***p*-CF3**, 0.329 for **tBuCz-*m*-CF3**, and 0.317 for **tBuCz-*p*-CF3**. These calculations suggest that an enhancement in molecular packing orientation has led to an increase in outcoupling efficiency. Additionally, the theoretically achievable values of EQE were computed and depicted in a contour map, as shown in Fig. S20b, with experimental EQEs and their corresponding simulated values indicated. An ideal correspondence between experimental and simulated EQEs for these phosphorescent OLED devices was demonstrated. Moreover, the electron- and hole-current behaviors have been investegated by the electron- and hole-only devices (EOD and HOD), where the device configurations consisted of (i) ITO / HAT-CN (10 nm) / BCFN (60 nm) / SiCzCz (5 nm) / EML (35 nm) / SiCzCz (5 nm) / BCFN (60 nm) / HAT-CN (10 nm) / Al, and (ii) ITO / Liq (2 nm) / mSiTrz: Liq (1:1, 31 nm) / mSiTrz (5 nm) / EML (35 nm) / mSiTrz (5 nm) / mSiTrz: Liq (1:1, 31 nm) / Liq (2 nm) / Al, respectively, as depicted in Fig. S21. Typically, the charge mobility of the hole is more rapid than that of the electron^23^. However, the electron-currents of all the devices depicted a higher process than those of hole-currents, which are in an agreement with recently reported work^16,46^, implied that the charge mobility orders of magnitude difference in electron compared to hole mobility has been solved. Furthermore, a comprehensive investigation into the hole and electron only devices of these Ir(III) complexes reveals that the hole/ electron charge currents of **tBuCz-*m*-CF3** and **tBuCz-*p*-CF3** are more balanced than that of ***m*-CF3** and ***p*-CF3** as the existence of tBuCz moiety. This observation could lead to significant implications for the performance and efficiency of electronic devices. Figure 3a shows schematic energy diagrams of the electron blocking layer (EBL, SiCzCz), hole blocking layer (HBL, mSiTrz) and emitters, with distinct differences in the relative HOMO energy levels of these emitters. The measured LUMO energy level of these emitters are ranged from −2.86 to −2.94 eV, corresponding to −2.72 eV of mSiTrz, indicating their hole-current can be confined in SiCzCz and SiTrzCz2; **tBuCz-*m*-CF3** and **tBuCz-*p*-CF3** dispayed a better confinement than those of ***m*-CF3** and ***p*-CF3**, as their HOMO energy level are shallower than that of SiCzCz (−5.52 eV), whereas the HOMO energy of ***m*-CF3** (−5.55 eV) and ***p*-CF3** (−5.50 eV) are comparable to that of SiCzCz. Therefore, the electron- and hole-carrier can be effectively confined in the emissive layer, and excitons are then generated and effectively transferred from the host to the emitters of **tBuCz-*m*-CF3** and **tBuCz-*p*-CF3**. With these observations, we concluded that our tBuCz based emitters, namely: **tBuCz-*m*-CF3** and **tBuCz-*p*-CF3** with tBuCz bulky group, have clearly shown the better electrochemical and photophysical data, reduced host-guest stacking, fast radiative rate constant, and even enhanced horizontal transition dipole orientation.

Inspiration by the success of TADF sensitized fluorescence (TSF) devices^47^, the hyper-OLEDs featuring the high color purity and durability have attracted significant attentions due to their effective energy transfer and excitons recombination in emissive layer^38^. Concurrently, *v*-DABNA^48^, an ideal candidate for achieving high color purity and efficiency^16^, has been frequently utilized as the terminal emitter in hyper devices due to its attributes of nearly 100% internal quantum efficiency, a near-perfect 100% emitting dipole orientation, and its characteristic of exhibiting narrow-band emission by multi-resonance effect^49^. To augment the device’s color purity, the complexes **tBuCz-*m*-CF3** and **tBuCz-*p*-CF3** were employed as phosphor sensitizers in phosphorescence sensitized fluorescence (PSF) devices, specifically for the narrowed-band saturated-blue MR emitter *ν*-DABNA, as depicted in Fig. S22a. Leveraging the substantial spectral overlap between the absorption spectrum of *v*-DABNA and the emission spectrum of phosphors in thin films, we demonstrate a more effective energy transfer within the emissive layer of the devices, as evidenced in Fig. S22b. Shown in Fig. 4a, their EL spectra of the PSF devices depicted a sharp blue peak at 472 nm, originated from *ν*-DABNA. Additionally, their FWHM values were approximately 20 nm, corresponding to CIE coordinates of (0.141, 0.233) and (0.140, 0.233), respectively. Additionally, their current density-voltage-luminance (J-V-L) characteristics also has been investigated to understand their EL behaviors, the Hyper-OLEDs based on **tBuCz-*m*-CF3** and **tBuCz-*p*-CF3** depicted a lower driving voltage of 2.75 and 2.80 V compared with the reported ν-DABNA-based hyper OLED devices with different sensitizers in Table S12, which demonstrated the enhancement of the charge injection from the electrodes to transporting layers, reducing the energy barriers and improving overall devices stability and efficiency. Their maximum CE, PE, and EQE were 42.20 cd A^−1^, 40.30 lm W^−1^, and 29.50% for **tBuCz-*m*-CF3**; 41.86 cd A^−1^, 40.27 lm W^−1^, and 29.78% for **tBuCz-*p*-CF3**, respectively, as numerous data summarized in Fig. 4c and Table 3. We have indeed conducted extensive studies on the outcoupling efficiency of hyper-OLEDs. Specifically, we measured the emitting dipole orientation of the thin film, which is a crucial factor influencing the outcoupling efficiency. Our results show that the orientation values are 0.98 for **tBuCz-*m*-CF3/ v-DABNA** and 0.95 for **tBuCz-*p*-CF3/ v-DABNA** in the co-host matrix. These high orientation values indicate that the dipoles are predominantly oriented in a direction that enhances light extraction. Based on these measurements, we calculated the theoretical EQEs for the hyper-OLEDs. For devices based on 5% Ir(III) complexes sensitized with 1% v-DABNA, the theoretical EQEs are 35.9% and 34.2%, respectively, and the values are depicted in Fig. S23.

**Fig. 5:**
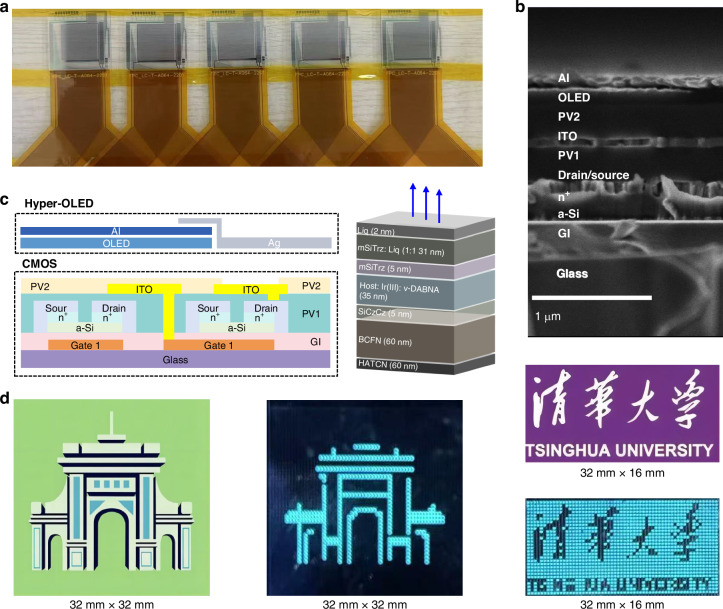
Microscopic and Performance of active-matrix hyper-OLED display. **a** Image of a single pixel in the TFT backplane displaying the arrangement of TFT and LED Regions; **b** Cross-sectional scanning electron microscope (SEM) images of the drive TFT and ITO, where the scale bar is 1 μm; **c** Structure diagram of the drive TFT and Hyper-OLED; **d** Digital photographs of the hyper-OLEDs operated at the switch state

The electroluminescence (EL) intensities at various current densities of 10, 20, and 50 mA cm⁻² were plotted against operational time for the **tBuCz-*m*-CF3/v-DABNA**-based hyper-devices, as shown in Fig. S24a. Additionally, the half-lifetime (LT_50_) values were plotted against the initial luminance (L₀) for the hyper-OLEDs, as depicted in Fig S24b. The exponent n was determined by fitting the LT₅₀ versus L₀ data on a log–log scale, yielding an n value of 1.79 for the **tBuCz-*m*-CF3/*v*-DABNA**-based hyper-devices. Notably, the LT_50_ values of the hyper-OLEDs were 302 and 318 h at 1000 cd m^−2^ in Fig. 4d, demonstrating a significant advancement in device stability, complemented by high efficiency, thereby validating the effectiveness of our molecular design approach in enhancing the stability of blue-emitting compounds. To identify the concept of AM OLEDs display, a thin film transistor (TFT) integrated with hyper-OLEDs based on **tBuCz-*m*-CF3** has been fabricated. The commercialized TFTs and their cross-sectional structures are depicted in Fig. 5a, b. Our Hyper-OLEDs, deposited on the TFT, and their diagram are depicted in Fig. 5c. Each pixel can be independently controlled by the TFT, as displayed in Fig. 5d, where clear pictures with uniform brightness are shown. As a results, the commercial potential of these Ir(III) complexes is underscored by their high durability, energy efficiency, and integration capabilities with thin film transistors for advanced display technologies.

## Discussion

Efficiency and operational lifetime are critical factors for the commercial success of blue phosphorescent OLEDs (Ph-OLEDs). Our research introduces novel Ir(III) complexes that significantly enhance both efficiency and operational lifetime in blue OLEDs. These improvements are attributed to three key factors: 1) The incorporation of highly bulky tBuCz groups suppresses intermolecular interactions and exciton quenching, promoting efficient radiative recombination, especially at high brightness levels. 2) The tBuCz-substituted Ir(III) emitters exhibit high horizontal dipole orientations (93% and 90%), which are crucial for enhancing light extraction and out-coupling efficiency. 3) Reduced efficiency roll-off: These Ir(III) complexes showed ultra-low efficiency roll-off and high EQE even at brightnesses as high as 100,000 cd m^‒2^. This indicates significant suppression of triplet-triplet and triplet-polaron annihilations, common causes of efficiency roll-off at high current densities. Operational lifetime, particularly under high luminance, is a significant challenge for blue Ph-OLEDs. Our Ir(III) complexes achieve lifetimes (LT_50_) of 1073 and 1237 h at 1000 cd m^-^², setting new benchmarks for blue-emitting OLED devices. These improved lifetimes are attributed to: High stability of emitters: The multi-dentate structures and functionalization enhance stability, making the emitters less susceptible to thermal and oxidative degradation. Effective exciton management: Dispersing excitons over a broader area of the EML and improving horizontal dipole orientation minimize triplet exciton concentration. The fast radiative decay rate constant (7.97 × 10^−5^ s^−1^) further reduces triplet exciton concentration in the EML. Device integration and optimization: Using an exciplex-forming co-host with novel emitters provides balanced carrier transport, reducing exciton and polaron accumulation within the device. The hyper-OLEDs also illustrated the comparable EQE_max_ are closed to 30%, corresponding to CIE_y_ of 0.233 with the LT_50_ over 300 h at 1000 cd m^−2^, demonstrating their high color purity, durability, energy efficiency, and integration capabilities with thin film transistors for advanced display technologies. In summary, these Ir(III) carbene pincer emitters demonstrate improved device efficiency and extended operational lifetime. These advancements are crucial for integrating these materials into next-generation displays and lighting solutions, where high efficiency and durability are essential.

## Materials and Methods

### Materials and characterization

All commercially available reagents were used directly without further purification. The materials for device fabrication were either purchased or synthesized by co-authors. UV-vis absorption spectra were obtained using a Cary 5000 UV-vis-NIR spectrophotometer (Agilent, USA) at room temperature with a concentration of 2.0 × 10^−5^ M. Phosphorescence spectra were measured at 77 K using an Edinburgh Instruments Ltd. FS5 spectrofluorometer. Transient PL decay curves were recorded using an Edinburgh FLSP920 fluorescence/phosphorescence spectrometer with time-dependent single photon counting technique, using a sodium lamp as the light source. Solid-state PL quantum yields were measured using a Hamamatsu C11347 instrument equipped with an integrating sphere, purged with dry argon to maintain an inert atmosphere. All samples were excited at 320 nm. The TFT was purchased from LinkZill Technology Co. Ltd.

### Device fabrication

ITO-coated glasses were precleaned in deionized water, acetone, and ethanol, then dried and treated with UV-ozone for 30 min. Device fabrication was conducted in a FS-450 chamber (Suzhou Fangsheng). Organic functional layers and metal electrodes were deposited via direct thermal vacuum evaporation at pressures below 4 × 10^–6^ Torr. Evaporation rates were 0.1 – 0.2 nm s^−1^ for organic materials, 0.01 nm s^−1^ for Liq, and 0.6 – 1.0 nm s^−1^ for Al to prevent oxidation. Ph-OLED device characteristics, including EL spectra, J-V-L curves, and CIE coordinates, were measured using a Keithley 2400 system. Device lifetimes were assessed with an OLED aging lifetime tester (ADVANTECH, 610 L).

## Supplementary information

supporting

## Data Availability

All data needed to evaluate the conclusions in the paper are present in the paper and/or the Supplementary Materials. Additional data related to this paper may be requested from the corresponding author.
