# Supplementary material for: Exceptionally high brightness and long lifetime of efficient blue OLEDs for programmable active-matrix display
Source: Light Sci Appl. 2025 Apr 9;14:156. doi: 10.1038/s41377-025-01817-x (PMC11982528; doi:10.1038/s41377-025-01817-x)
Supplement: Supplementary file 1 — supporting [file 41377_2025_1817_MOESM1_ESM.docx]

**Supplementary Information for**

**Exceptionally High Brightness and Long Lifetime of Efficient Blue OLEDs for Programmable Active-Matrix Display**

Chengcheng Wu^1,#^, Kai-Ning Tong^1,#^, Kefei Shi^1#^, Wei He^1^, Manli Huang^2^, Jie Yan^3^, Siqi Li^1^, Zhaoyun Jin^1^, Xin Wang^1^, Sinyeong Jung^1^, Jingrui Ma^4^, Yixi Zhuang^5^, Rong-Jun Xie^5^, Cunjiang Yu^6,7,*^, Hong Meng^8^, Xiao Wei Sun^4^, Chuluo Yang^2,^*, Yun Chi^3,^*, Feiyu Kang^1^, Guodan Wei^1,^*

Affiliations

^1^ Institute of Materials Research, Tsinghua Shenzhen International Graduate School (SIGS), Tsinghua University, Shenzhen 518055, China

^2^ Shenzhen Key Laboratory of Polymer Science and Technology, College of Materials Science and Engineering, Shenzhen University, Shenzhen 518060, China

^3^ Department of Materials Science and Engineering, Department of Chemistry, Center of Super-Diamond and Advanced Films (COSDAF), City University of Hong Kong, Kowloon Tong 999077, Hong Kong SAR

^4^ Institute of Nanoscience and Applications, Department of Electrical and Electronic Engineering, Southern University of Science and Technology, Shenzhen, Guangdong 518055, China

^5^ College of Materials and Fujian Key Laboratory of Surface andInterface Engineering for High Performance Materials, Xiamen University, Xiamen 361005, China

^6^ Department of Biomedical Engineering, Pennsylvania State University, University Park, PA 16802, USA

^7^ Department of Engineering Science and Mechanics, Department of Materials Science and Engineering, Materials Research Institute, Pennsylvania State University, University Park, PA 16802, USA

^8^ School of Advanced Materials, Peking University Shenzhen Graduated School, Peking University, Shenzhen, 518055, China

^#^These authors have contributed equally to this work

*Corresponding author. Guodan Wei: [weiguodan@sz.tsinghua.edu.cn](mailto:weiguodan@sz.tsinghua.edu.cn)

Yun Chi: [yunchi@cityu.edu.hk](mailto:yunchi@cityu.edu.hk)

Chuluo Yang: [clyang@szu.edu.cn](mailto:clyang@szu.edu.cn)

Cunjiang Yu: [cmy5358@psu.edu](mailto:cmy5358@psu.edu)

Contents

[Material synthesis 3](#_Toc191815386)

[**Fig. S1.** Synthetic pathway for the studied Ir(III) complexes. 3](#_Toc191815387)

[Thermal stability 8](#_Toc191815388)

[**Fig. S2.** Thermal stability of the studied Ir(III) complexes. 8](#_Toc191815389)

[**Table S1**. Thermophysical data of the studied Ir(III) complexes. 9](#_Toc191815390)

[Electrochemistry 9](#_Toc191815391)

[**Fig. S3**. Electrochemical characteristics of the studied Ir(III) complexes. 9](#_Toc191815392)

[DFT/ TD-DFT Calculation 10](#_Toc191815393)

[**Fig. S4.** Simulated absorption spectrum of (a) ***m*-CF3**, (b) ***p*-CF3**, (c) **tBuCz-*m*-CF3** and (d) **tBuCz-*p*-CF3**. 10](#_Toc191815394)

[**Table S2**. Calculated first 15 singlet excited state energies (λ / nm), the associated oscillator strengths (*f*) and the nature of the transitions at the optimized ground state (S_0_) geometries of ***m*-CF3**, ***p*-CF3**, **tBuCz-*m*-CF3** and **tBuCz-*p*-CF3** in the dichloromethane (DCM) by TD−B3LYP. 11](#_Toc191815395)

[**Fig. S5.** The DFT calculation. (a) The optimized geometrical and electronic structures calculated by B3LYP / 6-31G (d, p) method. (b) Electrostatic potential (ESP) analysis of the studied Ir(III) complexes; (c) Root mean square deviation (RMSD) of the studied Ir(III) complexes**. 14**](#_Toc191815396)

[**Fig. S6.** Spatial plots (isovalue = 0.02) of selected molecular orbitals of ***m*-CF3** at the optimized B3LYP ground-state geometry. 14](#_Toc191815397)

[**Fig. S7.** Spatial plots (isovalue = 0.02) of selected molecular orbitals of ***p*-CF3** at the optimized B3LYP ground-state geometry. 15](#_Toc191815398)

[**Fig. S8.** Spatial plots (isovalue = 0.02) of selected molecular orbitals of **tBuCz-*m*-CF3** at the optimized B3LYP ground-state geometry. 15](#_Toc191815399)

[**Fig. S9.** Spatial plots (isovalue = 0.02) of selected molecular orbitals of **tBuCz-*p*-CF3** at the optimized B3LYP ground-state geometry. 16](#_Toc191815400)

[**Table S3**. The major excitations contributing to the T_1_ states of ***m*-CF3**, ***p*-CF3**, **tBuCz-*m*-CF3** and **tBuCz-*p*-CF3** optimized with the TD-DFT/B3LYP method. 16](#_Toc191815401)

[**Fig. S10.** Spatial plots (isovalue = 0.02) of selected molecular orbitals of ***m*-CF3** at the optimized T_1_ state geometry. 17](#_Toc191815402)

[**Fig. S11.** Spatial plots (isovalue = 0.02) of selected molecular orbitals of ***p*-CF3** at the optimized T_1_ state geometry. 17](#_Toc191815403)

[**Fig. S12.** Spatial plots (isovalue = 0.02) of selected molecular orbitals of **tBuCz-*m*-CF3** at the optimized T_1_ state geometry. 17](#_Toc191815404)

[**Fig. S13.** Spatial plots (isovalue = 0.02) of selected molecular orbitals of **tBuCz-*p*-CF3** at the optimized T_1_ state geometry. 18](#_Toc191815405)

[**Table S4**. Relative energies of the lowest triplet excited states (T_1_) of ***m*-CF3**, ***p*-CF3**, **tBuCz-*m*-CF3** and **tBuCz-*p*-CF3** optimized at the B3LYP level. 18](#_Toc191815406)

[**Table S5**. Cartesian coordinates for optimized structures of ***m*-CF3**. 18](#_Toc191815407)

[**Table S6**. Cartesian coordinates for optimized structures of ***p*-CF3**. 20](#_Toc191815408)

[**Table S7**. Cartesian coordinates for optimized structures of **tBuCz-*m*-CF3**. 22](#_Toc191815409)

[**Table S8**. Cartesian coordinates for optimized structures of **tBuCz-*p*-CF3**. 24](#_Toc191815410)

[Photoluminescence Properties 28](#_Toc191815411)

[**Fig. S14** Multi-scan of PL spectra (a) ***m*-CF3**, (b) ***p*-CF3**, (c) **tBuCz-*m*-CF3** and (d) **tBuCz-*p*-CF3** at 2 10^-5^ M in toluene under irradiation of 150 W xenon lamp.. 28](#_Toc191815412)

[**Fig. S15**. Photophysical properties of the studied Ir(III) emitters in PMMA matrix with concentration of 2 wt% at room temperature. (a) emission spectra. (b) PL decay curves. 28](#_Toc191815413)

[**Table S9**. Summary of photophysical properties of the studied Ir(III) emitters in PMMA matrix with concentration of 2 wt% at room temperature. 29](#_Toc191815414)

[Electroluminescent Performance 30](#_Toc191815415)

[**Fig. S16.** Electroluminescent performances based on **tBuCz-*m*-CF3** doped in EML with varied thickness and doping concentrations. 30](#_Toc191815416)

[**Table S10.** OLED performances based on **tBuCz-*m*-CF3** with varied EML thickness and doping concentrations. 31](#_Toc191815417)

[**Fig. S17**. The measurement of device stability. 31](#_Toc191815418)

[**Fig. S18.** Angle dependent PL (ADPL) measurement showing the experimental and fitting result of horizontal dipole ratio (Θ_//_). 32](#_Toc191815419)

[**Fig. S19.** Refractive index of organic materials used in OLED device that were measured by variable angle spectroscopic ellipsometry (VASE). Red line represents refractive index (n) and blue line represents extinction coefficient (k). Ellipsometry data Ψ and Δ were fitted using isotropic general oscillator model provided by CompleteEase® program manufactured by J. A. Woollam. 33](#_Toc191815420)

[**Fig. S20**. a) Simulated outcoupling efficiency (air mode) and the probability of light dissipated to other modes. b) Simulated maximum achievable EQE values presented as a contour plot, with a color bar representing the estimated EQE values. 33](#_Toc191815421)

[**Figure S21**. Current density-voltage characteristics of the (a) electron only device; and (b) hole-only device for all the Ir(III) complexes; the EOD and HOD curves for (c) ***m*-CF3**, (d) ***p*-CF3**, (e) **tBuCz-*m*-CF3** and (d) **tBuCz-*p*-CF3**. 34](#_Toc191815422)

[**Figure S22**. (a)The molecular structure of *v*-DABNA; (b) the absorption of *v*-DABNA in toluene and the emissions of **tBuCz-*m*-CF3** and **tBuCz-*p*-CF3** in co-host matrix. 35](#_Toc191815423)

[**Figure S23.** Angle dependent PL (ADPL) measurement showing the experimental and fitting result of horizontal dipole ratio (Θ_//_) for (a) tBuCz-*m*-CF3/ v-DABNA and (b) tBuCz-*p*-CF3/ v-DABNA in co-host materix with the thickness of 35 nm; (c) Simulated outcoupling efficiency (air mode) and the probability of light dissipated to other modes. (d) Simulated maximum achievable EQE values presented as a contour plot, with a color bar representing the estimated EQE values. 35](#_Toc191815424)

[**Figure S24.** The measurement of hyper-OLED stability. 36](#_Toc191815425)

[**Table S11**. Reported performances of green to blue-emitting Ph-OLEDs based on Ir(III) phosphors. 37](#_Toc191815426)

[**Table S12.** EQE-CIEy relationship of recently reported ν-DABNA-based hyper OLED devices with different sensitizers. 38](#_Toc191815427)

[References 40](#_Toc191815428)

Material synthesis


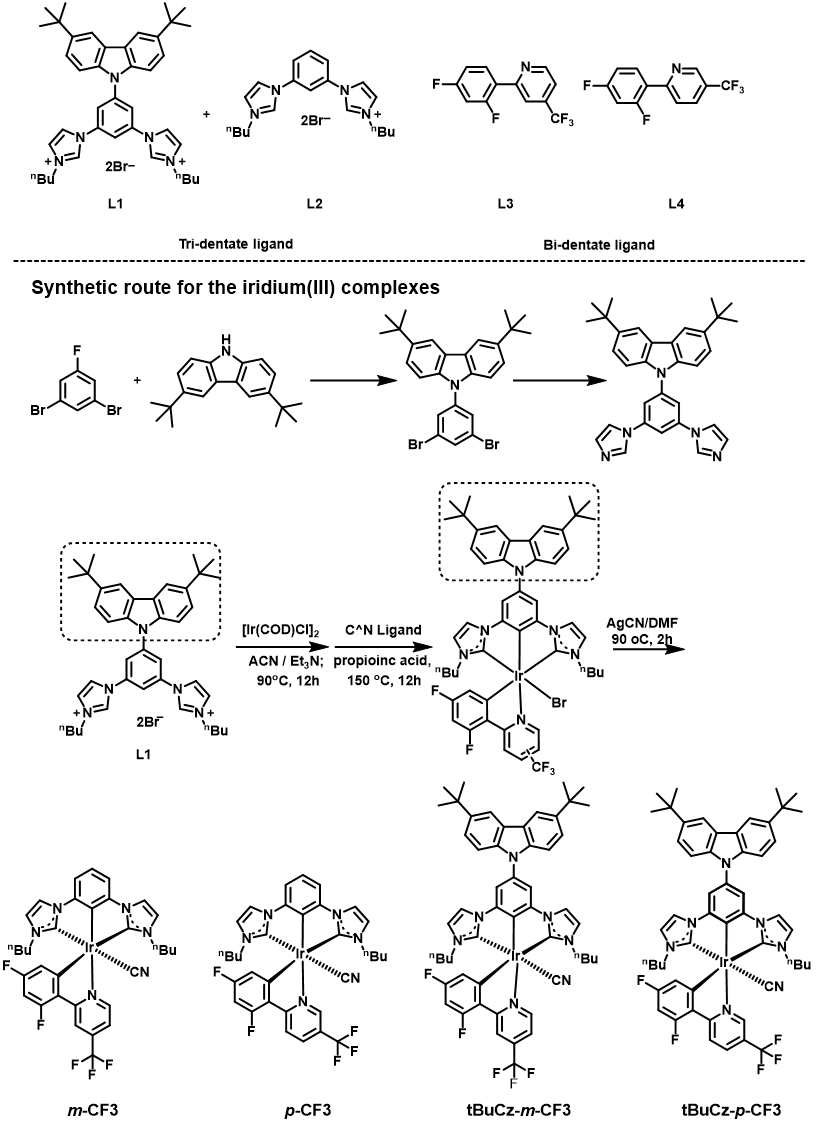


**Fig. S1.** Synthetic pathway for the studied Ir(III) complexes.

**Synthesis of 3,6-di-*tert*-butyl-9-(3,5-dibromophenyl)-9H-carbazole**

1,3-Dibromo-5-fluorobenzene (4.0 g, 15.8 mmol, 1.0 eq), 3,6-di-*tert*-butyl-9H-carbazole (5.33 g, 19.1 mmol, 1.2 eq), cesium carbonate (7.7 g, 31.6 mmol, 1.5 eq) and 100 mL of DMF were refluxed at 150 °C for 48 h under nitrogen atmosphere. The mixture was filtered and the organic layer is washed with water and extracted with DCM. It was then dried with anhydrous MgSO_4_ and concentrated to dryness under reduced pressure. The crude product was purified through silica gel column chromatography eluting with petroleum ether to obtain the desired product 3,6-di-*tert*-butyl-9-(3,5-dibromophenyl)-9H-carbazole as white powder (6.46 g, 80%).

^1^H NMR (400 MHz, DMSO-*d_6_*) δ 8.29 (d, J = 2.0 Hz, 2H), 7.97 (t, J = 1.7 Hz, 1H), 7.88 (d, J = 1.7 Hz, 2H), 7.51 (d, J = 2.0 Hz, 1H), 7.49 (d, J = 2.0 Hz, 1H), 7.35 (s, 1H), 7.33 (s, 1H), 1.41 (s, 18H).

**Synthesis of 3,6-di-*tert*-butyl-9-(3,5-di(1H-imidazol-1-yl)phenyl)-9H-carbazole**

3,6-Di-*tert*-butyl-9-(3,5-dibromophenyl)-9H-carbazole (3.20 g, 6.27 mmol, 1.0 eq), imidazole (1.07 g, 15.68 mmol, 2.5 eq), potassium carbonate (2.17 g, 15.68 mmol, 2.5 eq) and cupric oxide (0.5 g, 20%) was dissolved in 50 mL of DMSO, and then refluxed at 150 °C for 48 h under nitrogen atmosphere. After cooled to RT, the mixture was filtered and the filtrate was washed with water and extracted with DCM. The organic phase was collected and dried over anhydrous MgSO_4_. The solution was concentrated under reduced pressure, and petroleum ether was added and filtered to obtain 3,6-di-*tert*-butyl-9-(3,5-di(1H-imidazol-1-yl)phenyl)-9H-carbazole white solid (1.68 g, 55%).

^1^H NMR (400 MHz, DMSO-*d_6_*) δ 8.55 (s, 2H), 8.32 (s, 2H), 8.12 (t, J = 2.0 Hz, 1H), 8.04 (t, J = 1.4 Hz, 2H), 7.97 (d, J = 2.0 Hz, 2H), 7.54 – 7.46 (m, 4H), 7.16 (s, 2H), 1.43 (s, 18H).

**Synthesis of 1,1'-(5-(3,6-di-*tert*-butyl-9H-carbazol-9-yl)-1,3-phenylene)bis(3-butyl-1H-imidazol-3-ium) dibromide (L1)**

**L1**: 3,6-Di-*tert*-butyl-9-(3,5-di(1H-imidazol-1-yl)phenyl)-9H-carbazole (1.68 g, 3.45 mmol, 1.0 eq) and 1-bromobutane (2.36g, 17.2 mmol, 5.0 eq) were dissolved in 50 mL acetonitrile and refluxed at 100 °C under nitrogen atmosphere for 24 h. After cooled to RT, the volatiles were removed under reduced pressure, then diethyl ether was added and filtered to afford the colorless product (1.89 g, 91%).

^1^H NMR (400 MHz, DMSO-*d_6_*) δ 10.13 (s, 2H), 8.59 (s, 2H), 8.51 (s, 1H), 8.39 (d, J = 2.0 Hz, 2H), 8.36 (d, J = 1.9 Hz, 2H), 8.15 (t, J = 1.9 Hz, 2H), 7.63 (s, 1H), 7.61 (s, 1H), 7.54 (d, J = 2.0 Hz, 1H), 7.51 (d, J = 2.0 Hz, 1H), 4.31 (t, J = 7.3 Hz, 4H), 1.97 – 1.86 (m, 4H), 1.44 (s, 18H), 1.36 (dt, J = 14.7, 7.4 Hz, 4H), 0.95 (t, J = 7.4 Hz, 6H).

**L2: 1,3-Bis(1-butylimidazolium-3-yl)benzene dibromide (L2) is obtained using the same method as for L1.**

^1^H NMR (400 MHz, DMSO-*d_6_*) δ 10.21−10.16 (m, 2H), 8.55−8.53 (m, 2H), 8.49−8.47 (m, 2H), 8.15 (t, J = 1.8 Hz, 2H), 8.07−8.04 (m, 2H), 7.97 (dd, J = 9.2, 7.0 Hz, 1H), 4.31 (t, J = 7.2 Hz, 4H), 1.96– 1.88 (m, 4H), 1.40−1.29 (m, 4H), 0.95 (t, J = 7.4 Hz, 6H).

**L3**: 2,4-Difluorophenylboronic acid (1.91 g, 12 mmol), K_2_CO_3_ (6.8 g, 50 mmol) and Pd(PPh_3_)_4_ (0.578 g, 0.5 mmol) were placed in a 250 mL two-necked round bottom flask equipped with a condenser. The reaction flask was evacuated and filled with N_2_ gas three times. THF (70 mL), H_2_O (30 mL) and 2-bromo-4-trifluoromethylpyridine (2.26 g, 10 mmol) were then added. The mixture was refluxed for 24 h under N_2_ atmosphere. After cooled to RT, THF was removed by evaporation and the residue was dissolved in CH_2_Cl_2_ (150 mL). The precipitate was filtered, and the filtrate was washed with 1 N NaOH (2 × 50 mL) and sat. NaCl(aq) (50 mL) and then, dried over Na_2_SO_4_ and filtered. After evaporation of solvent, the reaction mixture was purified by silica gel column chromatography (EtOAc/n-hexane = 1 / 15) to provide 2-(2',4'-difuorophenyl)-4-trifluoromethylpyridine (**L3**, 2.15 g, 83.00 %) as a colorless solid.

^1^H NMR (400 MHz, chloroform-d) δ 8.90 (d, J = 5.1 Hz, 1H), 8.75 (dt, J = 9.6, 8.0 Hz, 1H), 8.16 – 8.05 (m, 1H), 7.61 – 7.51 (m, 1H), 7.02 (ddd, J = 8.3, 3.1, 0.9 Hz, 1H).

^19^F NMR (376 MHz, chloroform-d) δ -64.96, -66.35 (d, J = 9.9 Hz), -68.63 (d, J = 10.1 Hz).

**L4**: 2-(2',4'-Difuorophenyl)-5-trifluoromethylpyridine (**L4**) is obtained using the same method as for **L3**. (**L4**, 2.22 g, 85.70 %) as a colorless solid.

^1^H NMR (400 MHz, chloroform-d) δ 9.00 (dd, J = 2.2, 1.1 Hz, 1H), 8.87 – 8.75 (m, 1H), 8.12 – 8.00 (m, 2H), 7.05 (ddd, J = 8.3, 3.0, 0.9 Hz, 1H).

^19^F NMR (376 MHz, chloroform-d) δ -62.46, -65.89 (d, J = 10.2 Hz), -68.15 (d, J = 10.0 Hz).

**Synthesis of the Ir(III) intermediates**

A solution of [Ir(COD)(μ-Cl)]_2_ (500 mg, 0.75 mmol) and two equiv. of carbene pincer ligands (**L1** or **L2**) in 20 mL of acetonitrile (ACN) was heated at 90 °C for 12 hours. After removal of solvent, it was further added 1.5 equiv. of C^N ligands (**L3** or **L4**) and 20 mL of propionic acid. This mixture was next stirred at 150 °C overnight. After cooled to RT, the solvent was removed in vacuo. The crude product was separated through silica gel column chromatography (eluting with petroleum ether (PE) and to CH_2_Cl_2_ / PE, v/ v = 10/ 1) to obtain a yellow solid that was recrystallized from acetonitrile, and then, filtered and dried in air.

**General synthetic procedure of Ir(III) complexes.**

Under the N_2_ atmosphere, the intermediate complex and two eq. of of AgCN were dissolved in a round-bottom flask containing 60 mL of N,N-dimethylformamide (DMF). The suspension was heated at 110 °C for 2 h. After cooled to RT, the solution was filtered and solvent was removed by rotary evaporation to afford the crude product. It was purified by silica gel column chromatography to afford the Ir(III) product, followed by recrystallization from a mixed CH_2_Cl_2_ and diethyl ether solution.

***m*-CF3*,* yellow solid 283 mg, total yield: 88.7%**

^1^H NMR (400 MHz, chloroform-*d*) δ 10.47 (d, *J* = 5.9 Hz, 1H), 8.60 (d, *J* = 4.4 Hz, 1H), 7.52 – 7.43 (m, 3H), 7.24 (s, 1H), 7.16 (d, *J* = 7.7 Hz, 2H), 6.79 (d, *J* = 2.1 Hz, 2H), 6.32 (ddd, *J* = 13.3, 8.9, 2.4 Hz, 1H), 5.54 (dd, *J* = 8.5, 2.5 Hz, 1H), 3.31 – 3.12 (m, 4H), 1.47 – 1.34 (m, 2H), 1.14 – 1.02 (m, 2H), 0.97 – 0.65 (m, 10H).

^19^F NMR (376 MHz, chloroform-*d*) δ -64.82, -107.50 (d, *J* = 10.0 Hz), -109.78 (d, *J* = 10.8 Hz).

ESI-MS calcd for C_33_H_30_F_5_IrN_6_: [M+1]^+^, 799.2154, found 799.2142 ([M+1]^+^).

***p-*CF3**, **yellow solid 290 mg, total yield: 88.1%**

^1^H NMR (400 MHz, chloroform-*d*) δ 10.46 (s, 1H), 8.51 (dd, *J* = 8.8, 3.7 Hz, 1H), 8.10 (dd, *J* = 8.7, 2.2 Hz, 1H), 7.49 (d, *J* = 2.1 Hz, 2H), 7.24 (s, 1H), 7.21 – 7.11 (m, 2H), 6.79 (d, *J* = 2.1 Hz, 2H), 6.30 (ddd, *J* = 13.3, 8.8, 2.5 Hz, 1H), 5.53 (dd, *J* = 8.5, 2.5 Hz, 1H), δ 3.24 (ddd, *J* = 13.4, 9.8, 6.1 Hz, 2H), 3.10 (ddd, *J* = 13.4, 9.7, 5.8 Hz, 2H), 1.46 (dddd, *J* = 13.6, 9.7, 6.7, 4.0 Hz, 2H), 1.21 (dddd, *J* = 13.5, 9.7, 6.7, 4.0 Hz, 2H), 0.96 – 0.80 (m, 4H), 0.75 (t, *J* = 7.1 Hz, 6H).

^19^F NMR (377 MHz, chloroform-*d*) δ -61.99, -106.93, -109.00 (d, *J* = 12.9 Hz).

ESI-MS calcd for C_33_H_30_F_5_IrN_6_: [M+1]^+^ 799.2154, found 799.2146 ([M+1]^+^).

**tBuCz-*m*-CF3**: **yellow solid 354 mg, total yield: 94.2%**

^1^H NMR (400 MHz, chloroform-*d*) δ 10.51 (d, *J* = 5.9 Hz, 1H), 8.64 (d, *J* = 4.5 Hz, 1H), 8.20 (d, *J* = 1.9 Hz, 2H), 7.53 (dt, *J* = 9.7, 4.8 Hz, 3H), 7.47 – 7.41 (m, 4H), 7.35 (s, 2H), 6.82 (d, *J* = 2.1 Hz, 2H), 6.39 (ddd, *J* = 13.2, 8.8, 2.5 Hz, 1H), 5.68 (dd, *J* = 8.5, 2.5 Hz, 1H), 3.36 – 3.26 (m, 2H), 3.26 – 3.16 (m, 2H), 1.51 (s, 18H), 1.46 – 1.38 (m, 2H), 1.17 – 1.08 (m, 2H), 0.91 (dt, *J* = 13.3, 7.0 Hz, 2H), 0.79 (s, 2H), 0.76 – 0.71 (m, 6H).

^19^F NMR (376 MHz, chloroform-*d*) δ -64.82, -106.72 (d, *J* = 10.9 Hz), -109.42 (d, *J* = 10.5 Hz).

ESI-MS calcd for C_53_H_54_F_5_IrN_7_: [M+1]^+^ 1076.3985, found 1076.3969 ([M+1]^+^).

**tBuCz-*p*-CF3**: **yellow solid 386 mg, total yield: 90.5%**

^1^H NMR (400 MHz, chloroform-*d*) δ 10.55 (s, 1H), 8.56 (dd, *J* = 8.7, 3.7 Hz, 1H), 8.20 (d, *J* = 1.8 Hz, 2H), 8.17 – 8.11 (m, 1H), 7.54 (dd, *J* = 8.6, 1.9 Hz, 2H), 7.48 – 7.42 (m, 4H), 7.34 (s, 2H), 6.82 (d, *J* = 2.1 Hz, 2H), 6.37 (ddd, *J* = 13.3, 8.7, 2.5 Hz, 1H), 5.67 (dd, *J* = 8.4, 2.4 Hz, 1H), 3.30 (ddd, *J* = 13.5, 9.7, 6.1 Hz, 2H), 3.21 – 3.12 (m, 2H), 1.51 (s, 18H), 1.46 (d, *J* = 5.6 Hz, 2H), 1.31 – 1.23 (m, 2H), 0.98 – 0.85 (m, 4H), 0.78 (t, *J* = 7.1 Hz, 6H).

^19^F NMR (376 MHz, chloroform-*d*) δ -61.94, -106.28, -108.66.

ESI-MS calcd for C_53_H_54_F_5_IrN_7_: [M+1]^+^ 1076.3985, found 1076.3970 ([M+1]^+^).

Thermal stability


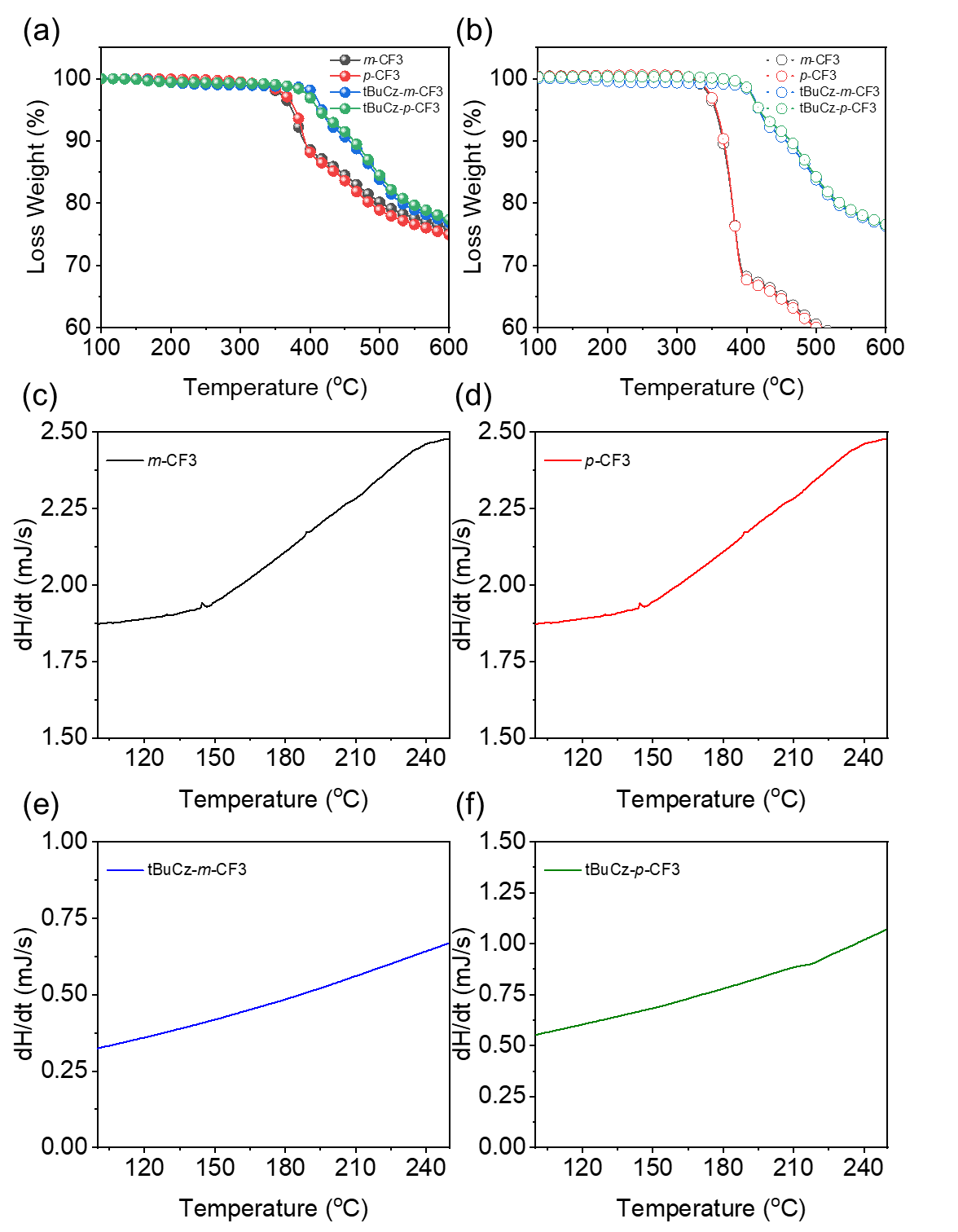


Fig. S2. Thermal stability of the studied Ir(III) complexes.

(a) The thermogravimetric analysis (TGA) curves before vacuum deposition, and (b) after vacuum deposition process; Differential scanning calorimetry (DSC) analysis for (c) ***m*-CF3**, (d) ***p*-CF3**, (e) **tBuCz-*m*-CF3** and (f) **tBuCz-*p*-CF3**.

Table S1. Thermophysical data of the studied Ir(III) complexes.

| **Complex** | **T_d_^a^ / °C** | **T_d_^b^ / °C** | **T_g_^c^ / °C** |
| --- | --- | --- | --- |
| ***m*-CF3** | 374 | 355 | 144 |
| ***p*-CF3** | 378 | 356 | 166 |
| **tBuCz-*m*-CF3** | 416 | 418 | - |
| **tBuCz-*p*-CF3** | 412 | 418 | 217 |

*^a^* Decomposition temperature (T_d_) is defined as the temperature that showed a 5 % weight loss.

*^b^* T_d_ recorded for the sublimed Ir(III) complexes.

*^c^* T*_g_* is defined as glass transition temperature.

Electrochemistry


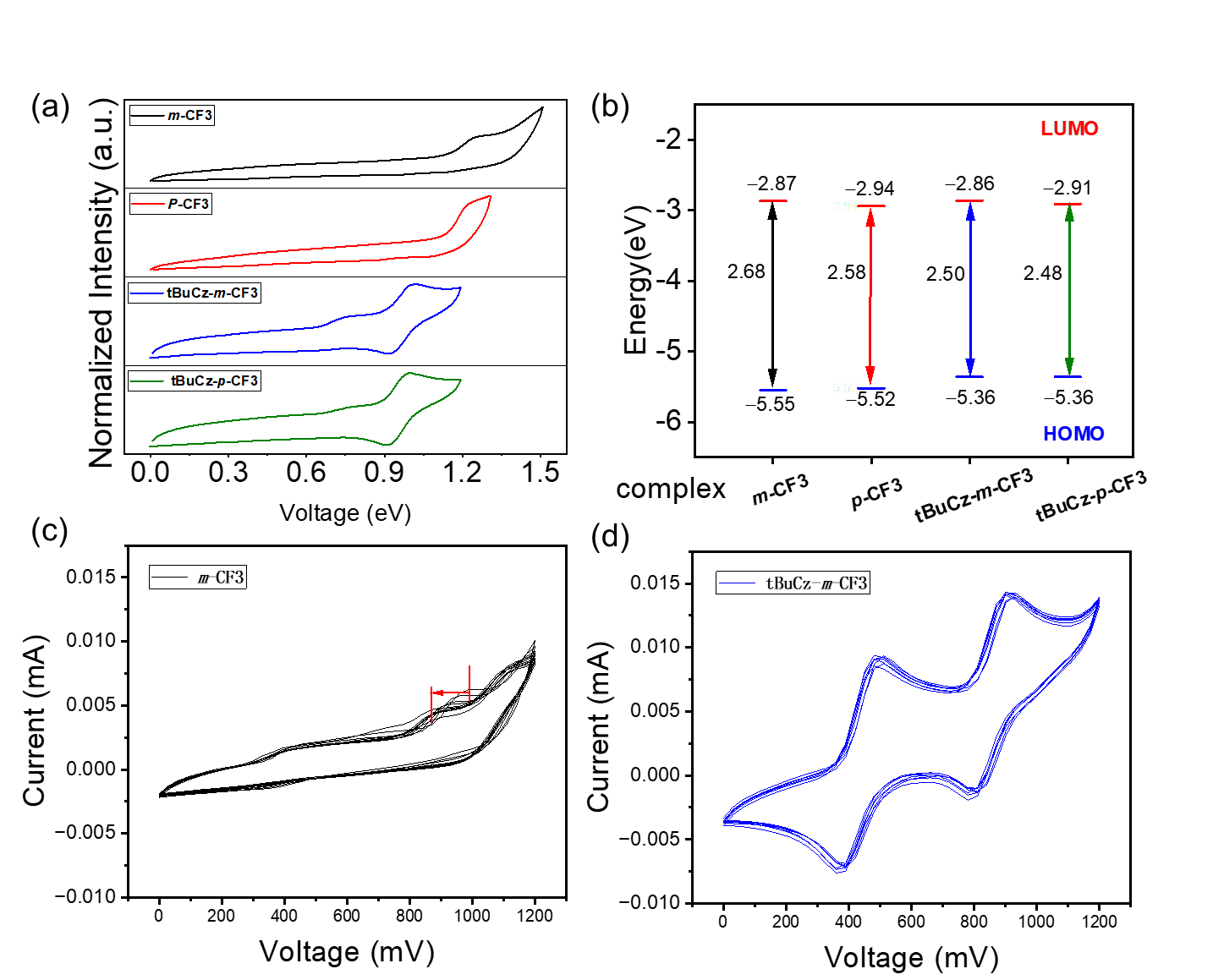


Fig. S3. Electrochemical characteristics of the studied Ir(III) complexes.

(a) The oxidation scan of the CV curves; (b) The measured HOMO / LUMO energy levels; repeated cyclic voltammograms of c) ***m*-CF3** (ten cycles); and d) **tBuCz-*m*-CF3** (ten cycles).

DFT/ TD-DFT Calculation

Density functional theory (DFT) and time-dependent density functional theory (TD-DFT) calculations were performed to understand the geometries and the electronic structures of all complexes using the Gaussian 16 package.^1^ The vibrational frequency calculations at the same level were carried out to verify that every optimized structure is an energy minimum (no imaginary frequency). B3LYP, 6−31G** (LANL2DZ)^2,3^ was used for both the geometry optimization and TDDFT calculations. The solvent effects were examined using the self−consistent reaction field (SCRF) method based on PCM models.^4,5^ The choice of solvents (DCM, a dielectric constant ε = 8.93) was based on the solvent media for experiments. Calculated first 15 singlet excited state energies (λ / nm), the associated oscillator strengths (*f*) and the nature of the transitions at the optimized ground state (S_0_) geometries of all complexes in the DCM by TD−B3LYP. The values in the parentheses are the % contributions of that particular configuration state function.


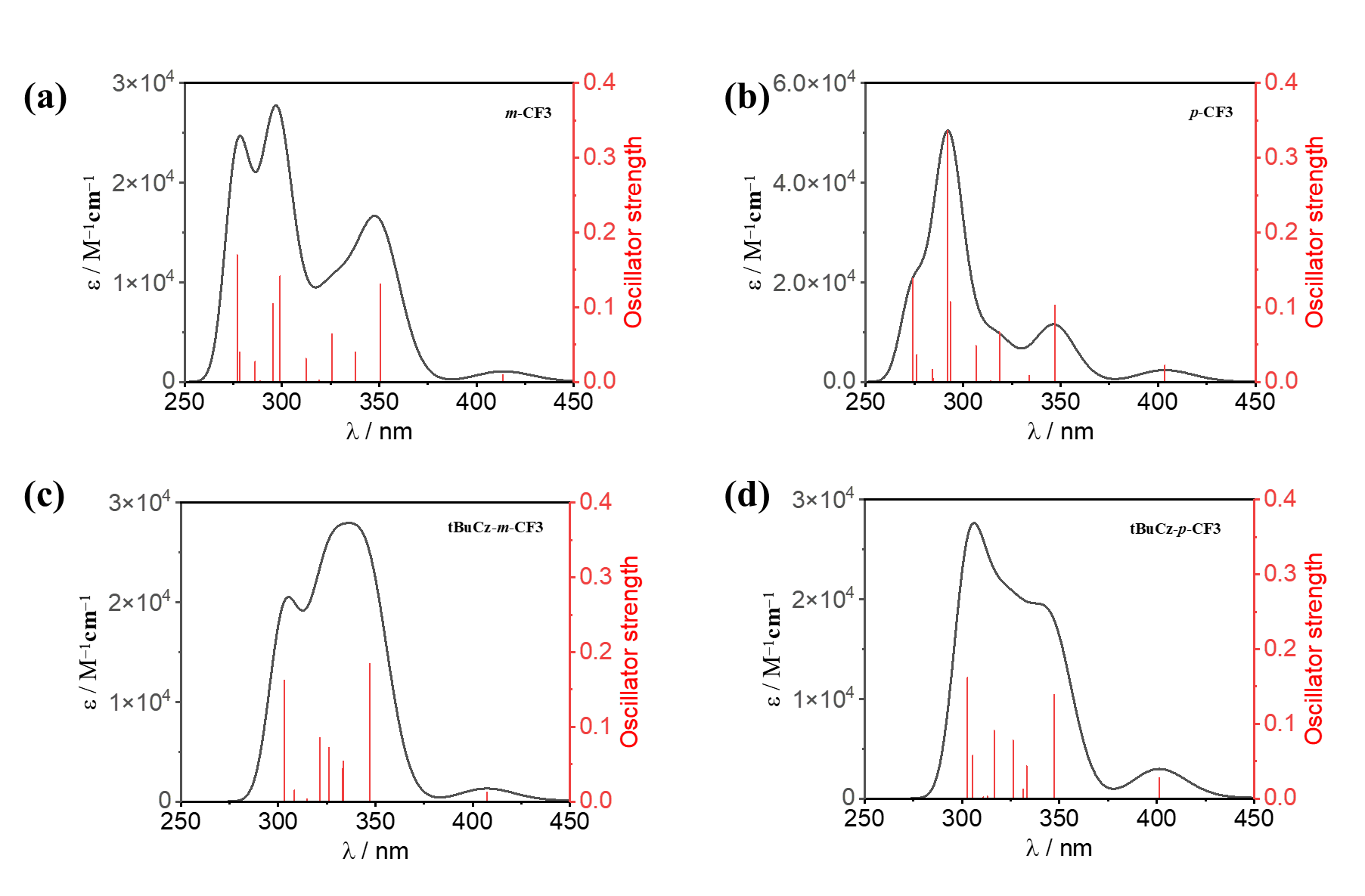


**Fig. S4.** Simulated absorption spectrum of (a) ***m*-CF3**, (b) ***p*-CF3**, (c) **tBuCz-*m*-CF3** and (d) **tBuCz-*p*-CF3**. The red vertical lines refer to the unbroadened oscillator strengths of the singlet–singlet transitions, and the black line is the fitting line of the UV−vis absorption.

**Table S2**. Calculated first 15 singlet excited state energies (λ / nm), the associated oscillator strengths (*f*) and the nature of the transitions at the optimized ground state (S_0_) geometries of ***m*-CF3**, ***p*-CF3**, **tBuCz-*m*-CF3** and **tBuCz-*p*-CF3** in the dichloromethane (DCM) by TD−B3LYP. The values in the parentheses are the % contributions of that particular configuration state function (CSF).

| ***m*-CF3** | Energy (eV) | λ (nm) | ƒ | Major contribs |
| --- | --- | --- | --- | --- |
| 1 | 2.9396 | 421.77 | 0.0001 | HOMO(H)→LUMO(L)(98.4%) |
| 2 | 2.9968 | 413.73 | 0.0097 | H-1→L (96.9%) |
| 3 | 3.5366 | 350.58 | 0.1303 | H-2→L (90.5%)  H-1→L+1 (5.0%) |
| 4 | 3.6174 | 342.74 | 0.0001 | H→L+1 (98.7%) |
| 5 | 3.6708 | 337.76 | 0.0395 | H-2→L (5.0%)  H-1→L+1 (92.1%) |
| 6 | 3.8076 | 325.62 | 0.0643 | H-3→L (87.7%)  H-2→L+1 (8.3%) |
| 7 | 3.885 | 319.14 | 0.0026 | H-4→L (97.1%) |
| 8 | 3.9679 | 312.47 | 0.0313 | H-5→L (69.6%)  H-2→L+1 (21.9%) |
| 9 | 4.1456 | 299.07 | 0.1416 | H-5→L (20.9%)  H-3→L (5.0%)  H-3→L+1 (4.2%)  H-2→L+1 (64.5%) |
| 10 | 4.198 | 295.34 | 0.1043 | H→L+2 (94.1%) |
| 11 | 4.2925 | 288.84 | 0.0012 | H-6→L (91.2%) |
| 12 | 4.3321 | 286.2 | 0.0274 | H-1→L+2 (79.1%)  H→L+3 (16.7%) |
| 13 | 4.355 | 284.69 | 0.0005 | H-7→L (93.4%) |
| 14 | 4.4555 | 278.27 | 0.0399 | H-1→L+3 (96.6%) |
| 15 | 4.4748 | 277.08 | 0.1697 | H-3→L+1 (84.9%) |
| ***p*-CF3** | Energy (eV) | λ (nm) | ƒ | Major contribs |
| 1 | 3.0083 | 412.14 | 0.0001 | HOMO(H)→LUMO(L)(98.5%) |
| 2 | 3.0722 | 403.57 | 0.0219 | H-1→L (96.8%) |
| 3 | 3.5724 | 347.06 | 0.1027 | H-2→L (94.7%) |
| 4 | 3.6645 | 338.34 | 0.0000 | H→L+1 (98.5%) |
| 5 | 3.7124 | 333.97 | 0.0085 | H-1→L+1 (96.9%) |
| 6 | 3.8906 | 318.67 | 0.0668 | H-3→L (85.8%)  H-2→L+1 (10.1%) |
| 7 | 3.9471 | 314.12 | 0.0015 | H-4→L (97.0%) |
| 8 | 4.0449 | 306.52 | 0.0483 | H-5→L (58.9%)  H-2→L+1 (32.6%) |
| 9 | 4.225 | 293.46 | 0.1069 | H→L+2 (94.0%) |
| 10 | 4.2466 | 291.96 | 0.3364 | H-5→L (29.6%)  H-3→L (6.9%)  H-2→L+1 (49.9%) |
| 11 | 4.3567 | 284.58 | 0.0046 | H-6→L (84.1%)  H-1→L+2 (6.2%) |
| 12 | 4.3622 | 284.22 | 0.0161 | H-6→L (7.3%)  H-1→L+2 (71.5%)  H→L+3 (15.9%) |
| 13 | 4.4325 | 279.72 | 0.0005 | H-7→L (93.4%) |
| 14 | 4.489 | 276.2 | 0.0368 | H-1→L+3 97.7%) |
| 15 | 4.5251 | 273.99 | 0.1385 | H-3→L+1 (84.9%) |
| **tBuCz-*m*-CF3** | Energy (eV) | λ (nm) | ƒ | Major contribs |
| 1 | 2.7401 | 452.48 | 0.00070 | HOMO(H)→LUMO(L) (91.0%)  H-3→L (8.6%) |
| 2 | 3.0437 | 407.35 | 0.01210 | H**-**2→L(86.3%)  H-1→L (11.0%) |
| 3 | 3.3053 | 375.11 | 0.00020 | H-3→L(88.6%)  H→L(8.8%) |
| 4 | 3.3882 | 365.93 | 0.00010 | H→L+1(93.4%)  H**-**3→L+1(6.0%) |
| 5 | 3.4475 | 359.64 | 0.00000 | H**-**1→L (88.8%)  H-2→L (11.1%) |
| 6 | 3.5707 | 347.23 | 0.18470 | H**-**4→L (90.8%) |
| 7 | 3.7165 | 333.60 | 0.05400 | H-2→L+1 (78.6%)  H-1→L+1 (10.2%) |
| 8 | 3.7209 | 333.21 | 0.04400 | H→L+2 (93.0%) |
| 9 | 3.8035 | 325.97 | 0.07240 | H→L+3 (95.0%) |
| 10 | 3.8560 | 321.54 | 0.08550 | H**-**5→L (83.7%)  H-4→L+1 (11.8%) |
| 11 | 3.9374 | 314.89 | 0.00360 | H-6→L (96.0%) |
| 12 | 3.9596 | 313.12 | 0.00040 | H-3→L+1 (91.9%)  H→L+1 (6.1%) |
| 13 | 4.0207 | 308.36 | 0.01540 | H-7→L (69.2%)  H-4→L+1 (13.0%)  H-8→L (8.0%) |
| 14 | 4.0664 | 304.90 | 0.00020 | H-1→L+1 (88.4%)  H→L+1 (11.5%) |
| 15 | 4.0910 | 303.07 | 0.16250 | H→L+4 (85.7%)  H-2→L+2 (7.4%) |
| **tBuCz-*p*-CF3** | Energy (eV) | λ (nm) | ƒ | Major contribs |
| 1 | 2.7657 | 448.29 | 0.00050 | HOMO(H)→LUMO(L) (91.6%)  H-3→L (8.1%) |
| 2 | 3.0894 | 401.32 | 0.02750 | H**-**2→L(85.9%)  H-1→L (11.2%) |
| 3 | 3.3319 | 372.11 | 0.00030 | H-3→L(87.0%)  H→L(8.2%) |
| 4 | 3.4234 | 362.17 | 0.00010 | H→L+1(90.4%)  H-3→L+1 (6.5%) |
| 5 | 3.4731 | 356.98 | 0.00000 | H-1→L (88.6%)  H-2→L (11.3%) |
| 6 | 3.5711 | 347.19 | 0.13910 | H-4→L (94.5%) |
| 7 | 3.7213 | 333.17 | 0.04350 | H→L+2 (97.1%) |
| 8 | 3.7406 | 331.46 | 0.01270 | H-2→L+1 (85.8%)  H-1→L+1 (11.2%) |
| 9 | 3.8011 | 326.18 | 0.07800 | H→L+3 (95.1%) |
| 10 | 3.9172 | 316.51 | 0.09100 | H**-**5→L (81.5%)  H-4→L+1 (13.2%) |
| 11 | 3.9617 | 312.96 | 0.00280 | H-6→L (93.5%) |
| 12 | 3.9874 | 310.94 | 0.00170 | H-3→L+1 (88.5%)  H→L+1 (6.9%) |
| 13 | 4.0582 | 305.52 | 0.05790 | H-7→L (54.5%)  H-4→L+1 (21.2%)  H→L+4 (9.4%)  H-8→L (6.9%) |
| 14 | 4.0950 | 302.77 | 0.16210 | H→L+4 (77.9%)  H-2→L+2 (7.5%)  H-7→L (7.1%) |
| 15 | 4.1120 | 301.52 | 0.00000 | H-1→L+1 (88.4%)  H-2→L+1 (11.5%) |

**
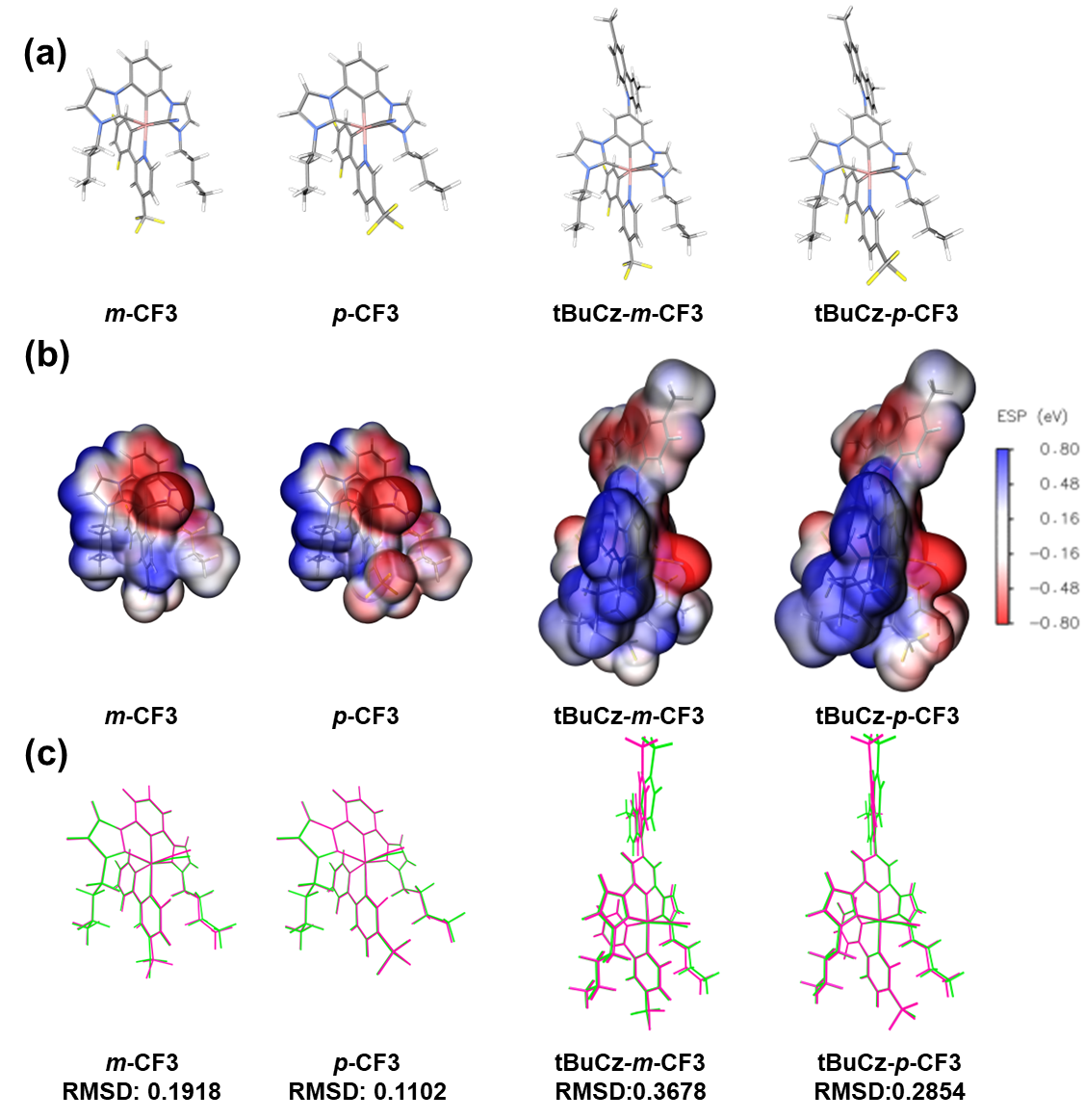
**

Fig. S5. The DFT calculation. (a) The optimized geometrical and electronic structures calculated by B3LYP / 6-31G (d, p) method. (b) Electrostatic potential (ESP) analysis of the studied Ir(III) complexes; (c) Root mean square deviation (RMSD) of the studied Ir(III) complexes.


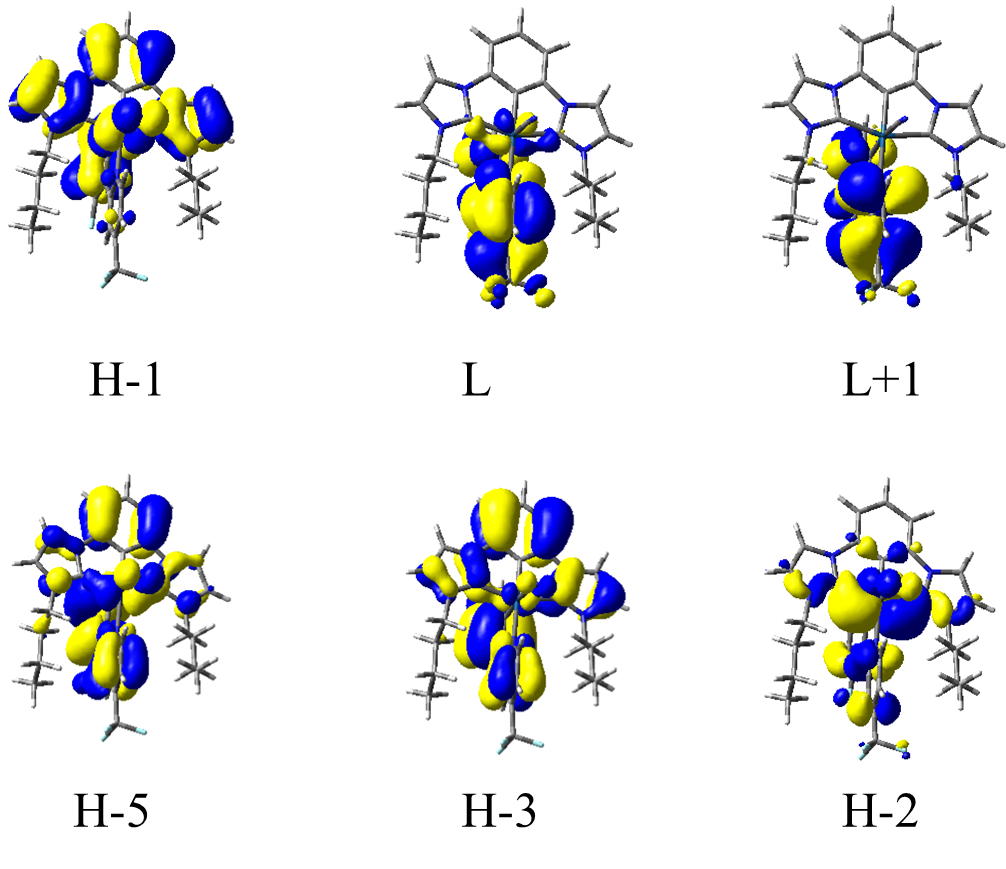


**Fig. S6.** Spatial plots (isovalue = 0.02) of selected molecular orbitals of ***m*-CF3** at the optimized B3LYP ground-state geometry.


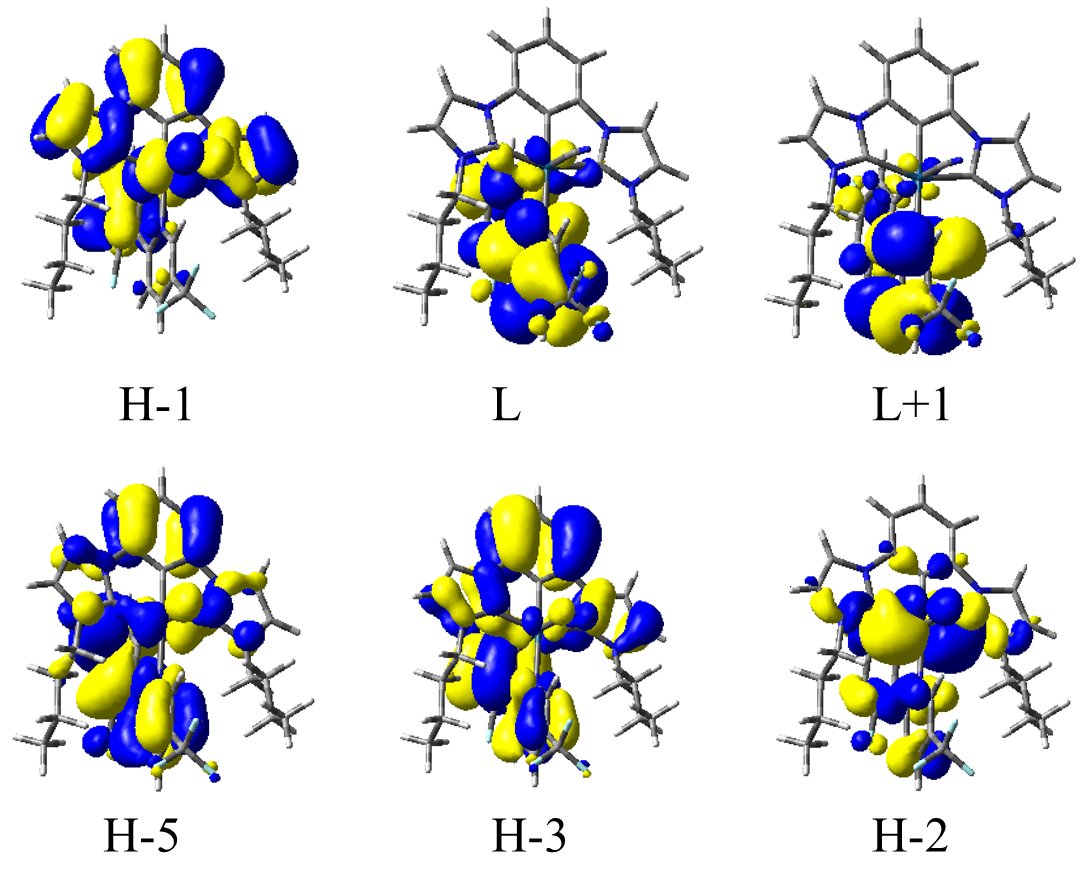


**Fig. S7.** Spatial plots (isovalue = 0.02) of selected molecular orbitals of ***p*-CF3** at the optimized B3LYP ground-state geometry.


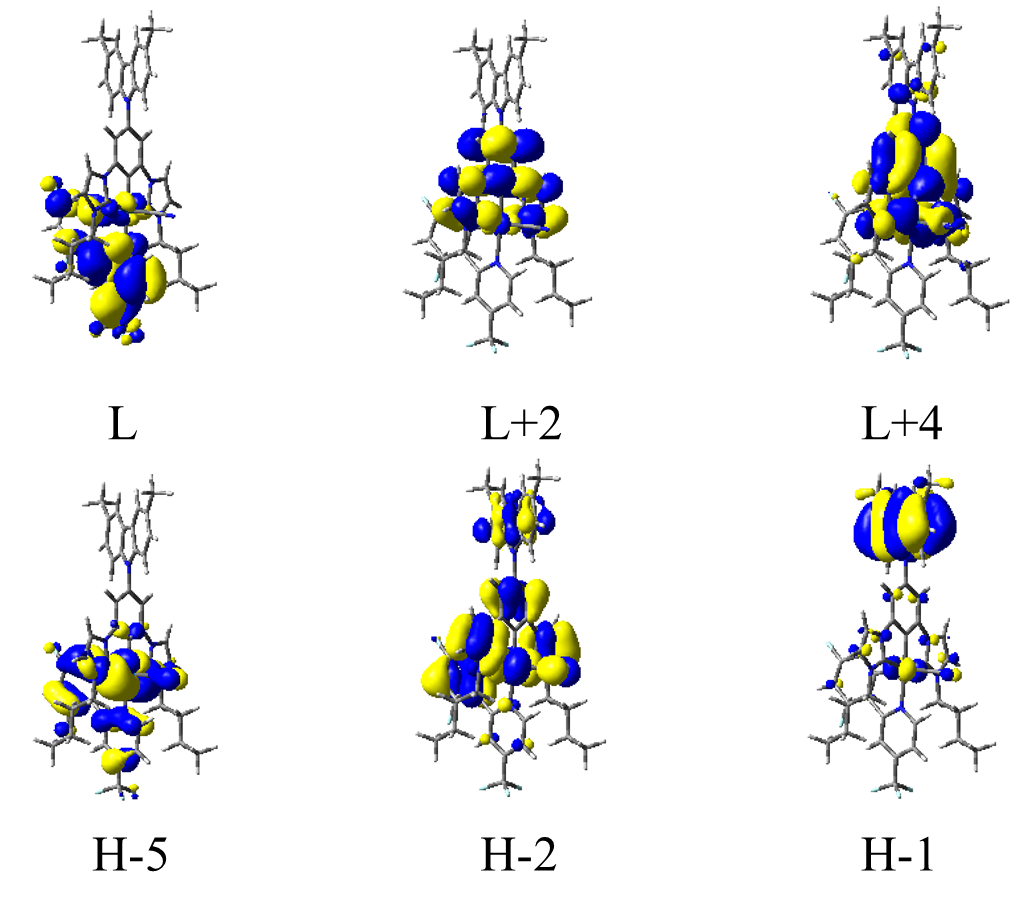


**Fig. S8.** Spatial plots (isovalue = 0.02) of selected molecular orbitals of **tBuCz-*m*-CF3** at the optimized B3LYP ground-state geometry.


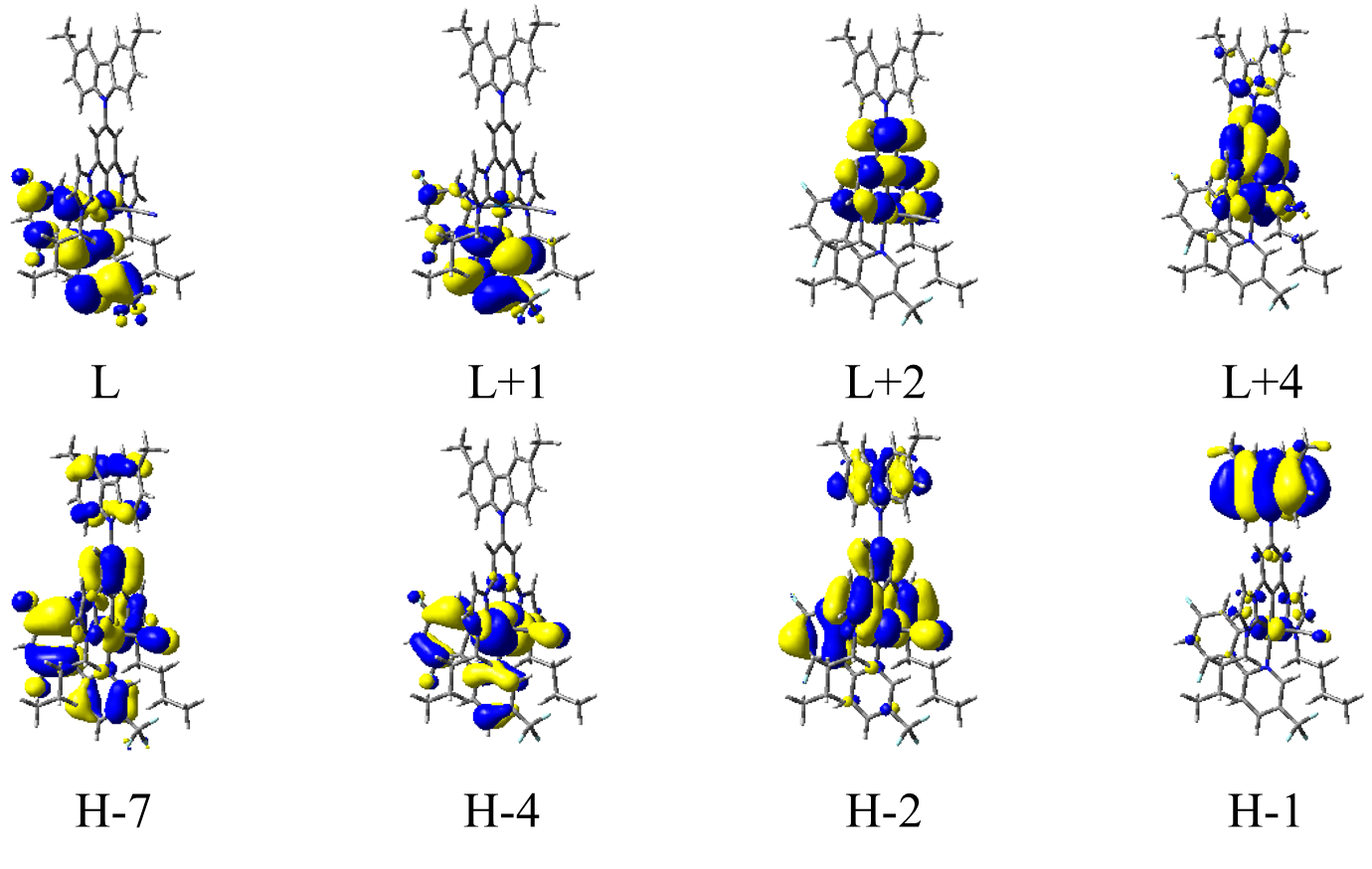


**Fig. S9.** Spatial plots (isovalue = 0.02) of selected molecular orbitals of **tBuCz-*p*-CF3** at the optimized B3LYP ground-state geometry.

**Table S3**. The major excitations contributing to the T_1_ states of ***m*-CF3**, ***p*-CF3**, **tBuCz-*m*-CF3** and **tBuCz-*p*-CF3** optimized with the TD-DFT/B3LYP method.

| Complex | Excitations contributing to the T_1_ state | Character |
| --- | --- | --- |
| ***m*-CF3** | HOMO–1→LUMO (93.2%) | ^3^LLCT /^3^MLCT |
|  | HOMO→LUMO (3.0%) | ^3^ILCT/^3^MLCT |
| ***p*-CF3** | HOMO–1→LUMO (26.2%) | ^3^LLCT /^3^MLCT |
|  | HOMO→LUMO (71.5%) | ^3^ILCT/^3^MLCT |
| **tBuCz-*m*-CF3** | HOMO–7→LUMO (3.7%) | ^3^LLCT /^3^MLCT/ ^3^ILCT |
|  | HOMO–5→LUMO (9.5%) | ^3^ILCT |
|  | HOMO–3→LUMO (21.0%) | ^3^LLCT /^3^MLCT/ ^3^ILCT |
|  | HOMO–1→LUMO (57.8%) | ^3^LLCT /^3^MLCT |
| **tBuCz-*P*-CF3** | HOMO–7→LUMO (9.3%) | ^3^LLCT /^3^MLCT/ ^3^ILCT |
|  | HOMO–6→LUMO (3.3%) | ^3^ILCT/^3^MLCT |
|  | HOMO–5→LUMO (10.2%) | ^3^LLCT /^3^MLCT/ ^3^ILCT |
|  | HOMO–4→LUMO (24.8%) | ^3^LLCT /^3^ILCT |
|  | HOMO–1→LUMO (44.0%) | ^3^LLCT /^3^MLCT |


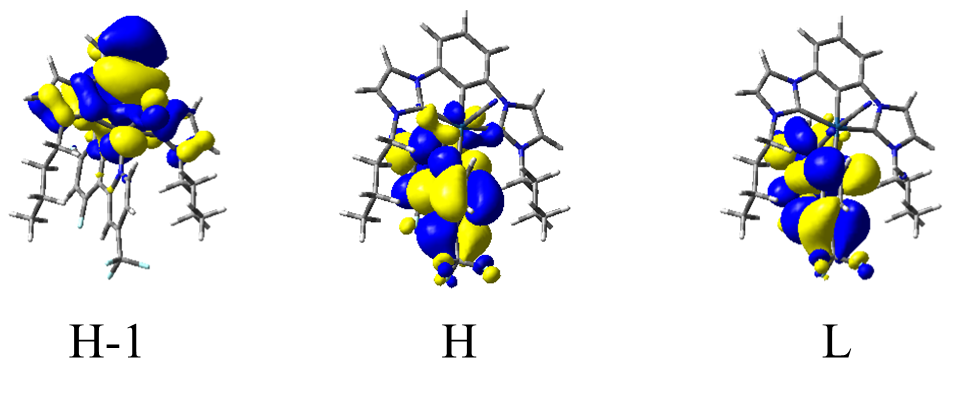


**Fig. S10.** Spatial plots (isovalue = 0.02) of selected molecular orbitals of ***m*-CF3** at the optimized T_1_ state geometry.


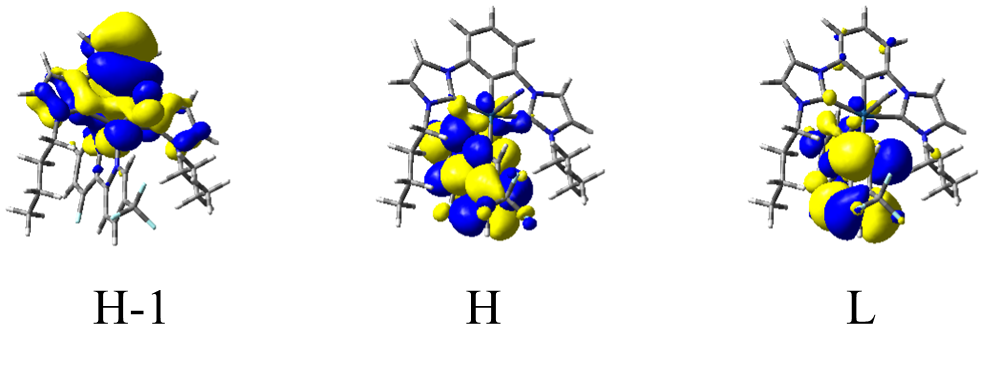


**Fig. S11.** Spatial plots (isovalue = 0.02) of selected molecular orbitals of ***p*-CF3** at the optimized T_1_ state geometry.


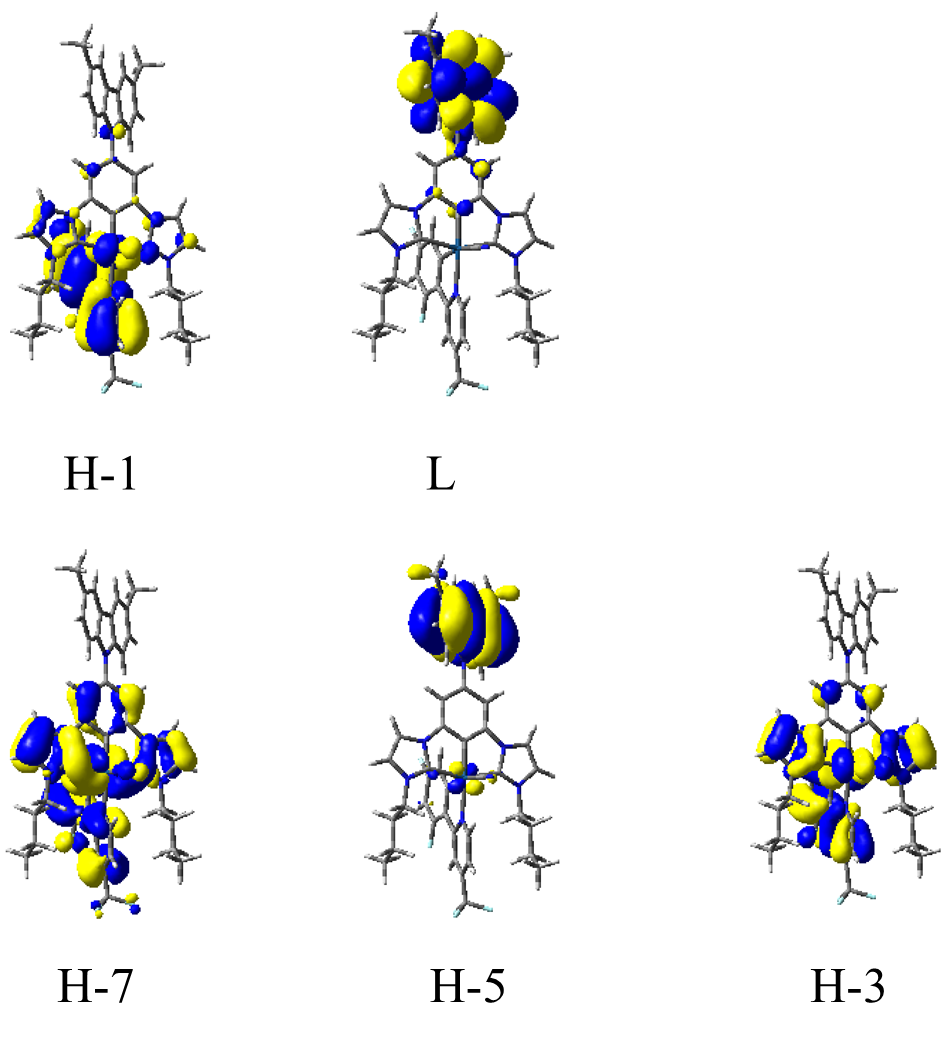


**Fig. S12.** Spatial plots (isovalue = 0.02) of selected molecular orbitals of **tBuCz-*m*-CF3** at the optimized T_1_ state geometry.


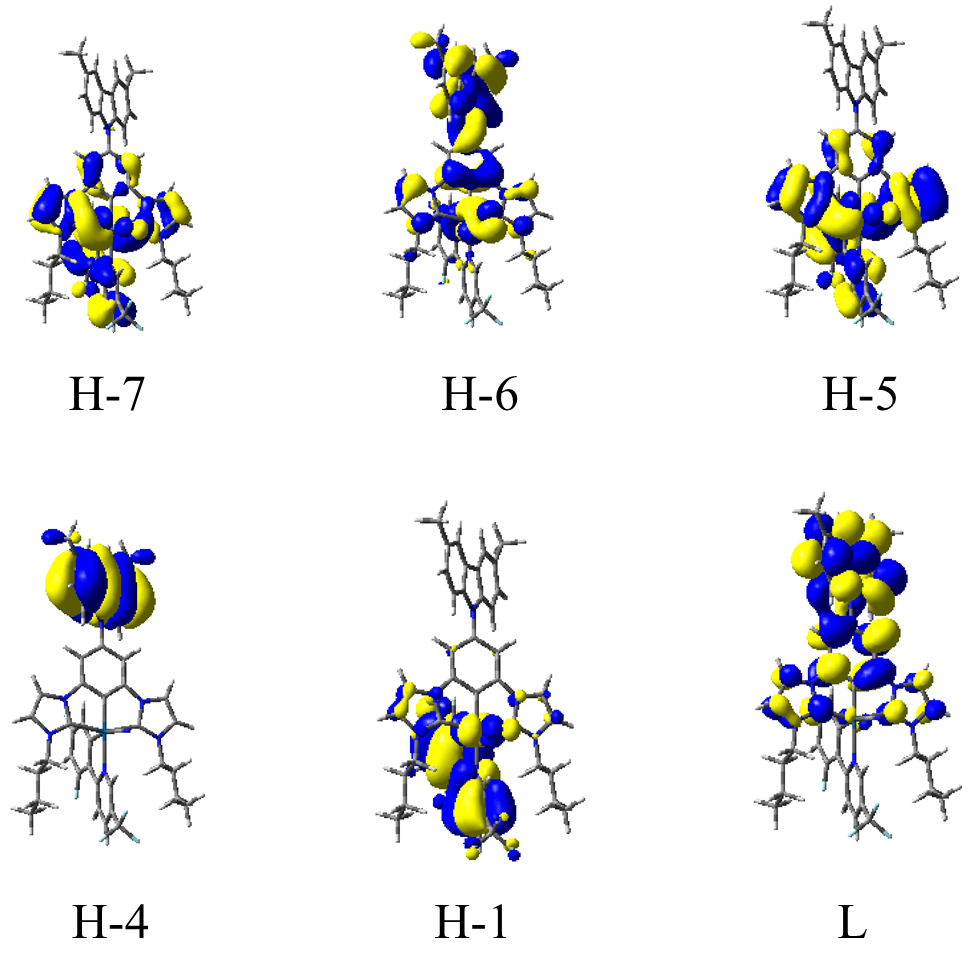


**Fig. S13.** Spatial plots (isovalue = 0.02) of selected molecular orbitals of **tBuCz-*p*-CF3** at the optimized T_1_ state geometry.

**Table S4**. Relative energies of the lowest triplet excited states (T_1_) of ***m*-CF3**, ***p*-CF3**, **tBuCz-*m*-CF3** and **tBuCz-*p*-CF3** optimized at the B3LYP level.

| Complex | ΔE(T_1_–S_0_)/cm^–1^ (λ/nm) |
| --- | --- |
| ***m*-CF3** | 21226.082 (471) |
| ***p*-CF3** | 21726.15 (460) |
| **tBuCz-*m*-CF3** | 19927.53 (502) |
| **tBuCz-*p*-CF3** | 20311.45 (492) |

**Table S5**. Cartesian coordinates for optimized structures of ***m*-CF3**.

| Ground state of ***m*-CF3** (S_0_) | | | | Excited state of ***m*-CF3** (T_1_) | | | |
| --- | --- | --- | --- | --- | --- | --- | --- |
| Ir | 1.03494 | 0.28472 | -0.4797 | Ir | 1.03328 | 0.28909 | -0.52555 |
| F | 2.25937 | -2.34091 | 4.15725 | F | 2.20654 | -2.07298 | 4.25576 |
| F | -2.2785 | -1.93621 | 3.08277 | F | -2.32706 | -1.76096 | 3.10638 |
| N | 2.63918 | 2.34062 | 0.82494 | N | 2.63497 | 2.39398 | 0.72333 |
| N | -1.12372 | 0.11526 | -0.47587 | N | -1.0626 | 0.12458 | -0.59336 |
| N | 2.98662 | -1.65314 | -1.44616 | N | 3.01357 | -1.69936 | -1.36099 |
| N | 1.11451 | -2.51068 | -2.09384 | N | 1.14203 | -2.63396 | -1.9014 |
| N | 0.62516 | 3.03471 | 1.17134 | N | 0.60865 | 3.02799 | 1.12738 |
| N | 0.87806 | 1.90201 | -3.26536 | N | 1.14249 | 1.78613 | -3.39116 |
| C | 1.62179 | -1.4437 | -1.43708 | C | 1.64815 | -1.51223 | -1.34846 |
| C | 0.70513 | -0.72428 | 1.28497 | C | 0.69274 | -0.64098 | 1.27095 |
| C | 3.61357 | 1.42165 | 0.35329 | C | 3.6179 | 1.47274 | 0.27151 |
| C | 1.31542 | 2.03621 | 0.57738 | C | 1.3144 | 2.05716 | 0.51236 |
| C | 3.79443 | -0.66376 | -0.82649 | C | 3.8151 | -0.66286 | -0.80906 |
| C | 4.99734 | 1.48084 | 0.51263 | C | 5.00232 | 1.55289 | 0.42524 |
| H | 5.48036 | 2.30267 | 1.03137 | H | 5.4779 | 2.39813 | 0.91142 |
| C | 3.00594 | 0.36241 | -0.31384 | C | 3.02173 | 0.38157 | -0.34939 |
| C | -0.67044 | -0.94749 | 1.60208 | C | -0.71611 | -0.84449 | 1.57173 |
| C | -0.3183 | -2.74794 | -2.25812 | C | -0.29167 | -2.90514 | -2.03218 |
| H | -0.45792 | -3.34403 | -3.16424 | H | -0.4302 | -3.54414 | -2.90794 |
| H | -0.78511 | -1.7758 | -2.42292 | H | -0.77938 | -1.95013 | -2.22882 |
| C | -1.65914 | -0.447 | 0.64792 | C | -1.66186 | -0.39417 | 0.61559 |
| C | 1.01466 | 1.31606 | -2.25996 | C | 1.13363 | 1.24421 | -2.3523 |
| C | 5.18187 | -0.64932 | -0.69109 | C | 5.2027 | -0.63174 | -0.67679 |
| H | 5.80434 | -1.44495 | -1.0876 | H | 5.83084 | -1.44432 | -1.02622 |
| C | 2.12691 | -3.37939 | -2.49743 | C | 2.16029 | -3.51562 | -2.25307 |
| H | 1.91596 | -4.28802 | -3.03759 | H | 1.95432 | -4.46645 | -2.71704 |
| C | 1.48676 | 3.95738 | 1.76244 | C | 1.45927 | 3.97445 | 1.69284 |
| H | 1.11986 | 4.82905 | 2.27957 | H | 1.08161 | 4.83791 | 2.21583 |
| C | -0.02436 | -2.13826 | 3.6456 | C | -0.07735 | -1.9115 | 3.69936 |
| H | -0.30113 | -2.68707 | 4.53613 | H | -0.35809 | -2.40976 | 4.61862 |
| C | -1.35609 | 3.72256 | -0.16677 | C | -1.47257 | 3.61363 | -0.11806 |
| H | -1.00517 | 3.0913 | -0.98969 | H | -1.13251 | 2.99067 | -0.95164 |
| H | -0.91484 | 4.71385 | -0.32856 | H | -1.10457 | 4.62984 | -0.30628 |
| C | 2.75429 | 3.51785 | 1.54765 | C | 2.73494 | 3.57245 | 1.44486 |
| H | 3.70391 | 3.9309 | 1.84393 | H | 3.67898 | 4.01194 | 1.7201 |
| C | 1.30571 | -1.88537 | 3.31703 | C | 1.26555 | -1.6733 | 3.37531 |
| C | 5.76583 | 0.43482 | -0.01933 | C | 5.77788 | 0.49099 | -0.0618 |
| C | 1.68048 | -1.20228 | 2.17019 | C | 1.65209 | -1.05748 | 2.18874 |
| H | 2.73507 | -1.05004 | 1.96986 | H | 2.70864 | -0.9102 | 1.99442 |
| C | -0.83299 | 3.14635 | 1.14755 | C | -0.85573 | 3.0792 | 1.17286 |
| H | -1.12671 | 3.77158 | 1.99555 | H | -1.12942 | 3.70372 | 2.02692 |
| H | -1.23978 | 2.14808 | 1.3179 | H | -1.20918 | 2.0665 | 1.3736 |
| C | -0.92981 | -3.43431 | -1.03564 | C | -0.87023 | -3.54766 | -0.77149 |
| H | -0.5139 | -4.44481 | -0.93545 | H | -0.4335 | -4.54442 | -0.63236 |
| H | -0.62287 | -2.88026 | -0.14382 | H | -0.5687 | -2.94564 | 0.09065 |
| C | 3.30578 | -2.83648 | -2.09452 | C | 3.33935 | -2.92693 | -1.91412 |
| H | 4.31964 | -3.1781 | -2.22041 | H | 4.35538 | -3.26705 | -2.02513 |
| C | -2.88455 | 3.82133 | -0.17492 | C | -3.0025 | 3.60498 | -0.04505 |
| H | -3.20811 | 4.5337 | 0.59545 | H | -3.33276 | 4.28258 | 0.75339 |
| H | -3.31919 | 2.85437 | 0.10513 | H | -3.34578 | 2.6044 | 0.2384 |
| C | -0.9822 | -1.65939 | 2.77146 | C | -1.02883 | -1.4961 | 2.80014 |
| C | -1.92358 | 0.55819 | -1.4626 | C | -1.87613 | 0.45462 | -1.62417 |
| H | -1.41358 | 0.97732 | -2.322 | H | -1.37033 | 0.80015 | -2.51829 |
| C | -3.30511 | 0.50353 | -1.38311 | C | -3.24395 | 0.39189 | -1.56204 |
| C | -3.05584 | -0.49396 | 0.79824 | C | -3.07244 | -0.40991 | 0.73049 |
| H | -3.48779 | -0.90529 | 1.69587 | H | -3.51835 | -0.73315 | 1.65847 |
| C | -3.87099 | -0.01358 | -0.21557 | C | -3.86842 | -0.01889 | -0.31587 |
| C | -3.43599 | 4.24602 | -1.53698 | C | -3.66218 | 4.00024 | -1.36663 |
| H | -3.1489 | 3.52929 | -2.31384 | H | -3.34384 | 3.32892 | -2.17087 |
| H | -3.04628 | 5.22532 | -1.83477 | H | -3.396 | 5.02242 | -1.65743 |
| H | -4.52859 | 4.30791 | -1.522 | H | -4.7522 | 3.93876 | -1.29391 |
| C | -2.45799 | -3.49316 | -1.10699 | C | -2.39807 | -3.63738 | -0.82627 |
| H | -2.76597 | -4.10057 | -1.96811 | H | -2.69755 | -4.2969 | -1.65115 |
| H | -2.84589 | -2.48415 | -1.29115 | H | -2.80711 | -2.64683 | -1.05724 |
| C | -3.0844 | -4.05193 | 0.1728 | C | -3.0032 | -4.13782 | 0.48701 |
| H | -2.74929 | -5.07782 | 0.36119 | H | -2.61377 | -5.1274 | 0.75132 |
| H | -4.17689 | -4.06258 | 0.10712 | H | -4.09268 | -4.21476 | 0.41513 |
| H | -2.80999 | -3.44898 | 1.04387 | H | -2.77197 | -3.45586 | 1.31078 |
| C | -5.36243 | 0.02877 | -0.03122 | C | -5.34178 | 0.07178 | -0.19173 |
| F | -5.75408 | 1.25399 | 0.39333 | F | -5.7793 | 1.36499 | -0.25866 |
| F | -5.79032 | -0.86721 | 0.88016 | F | -5.80797 | -0.43777 | 0.96941 |
| F | -6.01406 | -0.2159 | -1.18723 | F | -5.98111 | -0.57664 | -1.20251 |
| H | -3.91654 | 0.8704 | -2.19762 | H | -3.84246 | 0.67035 | -2.41978 |
| H | 6.84441 | 0.46424 | 0.09396 | H | 6.85607 | 0.53396 | 0.04902 |

**Table S6**. Cartesian coordinates for optimized structures of ***p*-CF3**.

| Ground state of ***p*-CF3** (S_0_) | | | | Excited state of ***p*-CF3** (T_1_) | | | |
| --- | --- | --- | --- | --- | --- | --- | --- |
| Ir | -0.77017 | -0.38198 | -0.43021 | Ir | -0.7694 | -0.38333 | -0.4303 |
| F | -3.55522 | 2.21325 | 3.48256 | F | -3.55764 | 2.21547 | 3.47779 |
| F | 1.10537 | 2.7488 | 3.50064 | F | 1.10292 | 2.75048 | 3.4995 |
| N | -2.29365 | -2.59717 | 0.69713 | N | -2.28869 | -2.60046 | 0.69937 |
| N | 1.24859 | 0.1589 | 0.02907 | N | 1.24846 | 0.16048 | 0.02769 |
| N | -2.70584 | 1.08036 | -2.05067 | N | -2.70895 | 1.0751 | -2.04966 |
| N | -0.91138 | 2.25949 | -2.29068 | N | -0.91683 | 2.2577 | -2.2903 |
| F | 4.49224 | -0.99671 | -2.12242 | F | 4.49364 | -0.99304 | -2.123 |
| N | -0.33749 | -2.87694 | 1.5721 | N | -0.33181 | -2.87647 | 1.57385 |
| N | 0.24943 | -2.18282 | -2.91328 | N | 0.25194 | -2.18266 | -2.91401 |
| C | -1.37768 | 1.15059 | -1.67851 | C | -1.38068 | 1.14774 | -1.67826 |
| F | 5.70966 | -0.92285 | -0.30984 | F | 5.7104 | -0.91777 | -0.31011 |
| C | -1.08488 | 0.78123 | 1.21566 | C | -1.08552 | 0.78123 | 1.21426 |
| C | -3.25431 | -1.91952 | -0.10121 | C | -3.25093 | -1.92488 | -0.09883 |
| C | -1.01861 | -2.06846 | 0.73479 | C | -1.01452 | -2.0696 | 0.73631 |
| C | -3.4692 | -0.00295 | -1.53549 | C | -3.47001 | -0.00936 | -1.53376 |
| C | -4.60359 | -2.22982 | -0.27084 | C | -4.59982 | -2.23738 | -0.26718 |
| H | -5.06119 | -3.08506 | 0.21558 | H | -5.05568 | -3.09309 | 0.22006 |
| C | -2.68324 | -0.81954 | -0.73082 | C | -2.68205 | -0.82436 | -0.72944 |
| C | 0.15484 | 1.30715 | 1.81193 | C | 0.15366 | 1.30839 | 1.81042 |
| C | 0.44511 | 2.77512 | -2.09959 | C | 0.43873 | 2.77592 | -2.09956 |
| H | 0.73583 | 3.30177 | -3.01264 | H | 0.72859 | 3.3025 | -3.01294 |
| H | 1.09892 | 1.91015 | -1.98508 | H | 1.09403 | 1.91216 | -1.98435 |
| F | 5.49011 | 0.85143 | -1.53522 | F | 5.48986 | 0.85604 | -1.53597 |
| C | 1.37986 | 0.94291 | 1.22037 | C | 1.37904 | 0.94562 | 1.21857 |
| C | -0.17297 | -1.53829 | -2.03099 | C | -0.17113 | -1.53888 | -2.03149 |
| C | -4.82284 | -0.27084 | -1.734 | C | -4.82343 | -0.27948 | -1.73103 |
| H | -5.44808 | 0.36154 | -2.35593 | H | -5.45014 | 0.35174 | -2.35266 |
| C | -1.91442 | 2.88192 | -3.02666 | C | -1.92151 | 2.87849 | -3.0255 |
| H | -1.73233 | 3.78095 | -3.59276 | H | -1.74143 | 3.77802 | -3.59143 |
| C | -1.15048 | -3.90577 | 2.04012 | C | -1.14284 | -3.90649 | 2.04257 |
| H | -0.78117 | -4.66141 | 2.71438 | H | -0.77203 | -4.66111 | 2.71714 |
| C | -1.20901 | 2.47242 | 3.50478 | C | -1.21147 | 2.47451 | 3.50169 |
| H | -1.27599 | 3.1312 | 4.36181 | H | -1.27915 | 3.13377 | 4.35828 |
| C | 1.94635 | -3.38224 | 0.74572 | C | 1.95235 | -3.37972 | 0.74732 |
| H | 1.69109 | -2.91408 | -0.21087 | H | 1.6967 | -2.91264 | -0.20971 |
| H | 1.67889 | -4.44264 | 0.65419 | H | 1.68569 | -4.44041 | 0.65678 |
| C | -2.38401 | -3.72542 | 1.49512 | C | -2.37683 | -3.72853 | 1.49781 |
| H | -3.29541 | -4.28793 | 1.61067 | H | -3.28718 | -4.29262 | 1.61389 |
| C | -2.36434 | 1.91294 | 2.92288 | C | -2.36638 | 1.91477 | 2.91924 |
| C | -5.37061 | -1.39381 | -1.09509 | C | -5.36888 | -1.40299 | -1.09124 |
| H | -6.42186 | -1.61914 | -1.23931 | H | -6.41989 | -1.63008 | -1.23447 |
| C | -2.30808 | 1.09347 | 1.80165 | C | -2.30923 | 1.09431 | 1.79887 |
| H | -3.23315 | 0.70807 | 1.38773 | H | -3.2338 | 0.70805 | 1.38462 |
| C | 1.09614 | -2.74493 | 1.84416 | C | 1.10174 | -2.74201 | 1.84519 |
| H | 1.28872 | -3.21096 | 2.81456 | H | 1.29543 | -3.20638 | 2.81618 |
| H | 1.31287 | -1.67933 | 1.9349 | H | 1.31688 | -1.67598 | 1.93421 |
| C | 0.54022 | 3.68169 | -0.87214 | C | 0.53209 | 3.68344 | -0.87267 |
| H | -0.06978 | 4.5803 | -1.03027 | H | -0.07923 | 4.58102 | -1.03142 |
| H | 0.10575 | 3.15034 | -0.0199 | H | 0.09829 | 3.15193 | -0.02019 |
| C | -3.04416 | 2.13741 | -2.87782 | C | -3.04969 | 2.13171 | -2.87637 |
| H | -4.03112 | 2.26364 | -3.29054 | H | -4.03715 | 2.25625 | -3.28847 |
| C | 3.44527 | -3.24303 | 1.02339 | C | 3.45116 | -3.23921 | 1.02491 |
| H | 3.69003 | -3.74539 | 1.96903 | H | 3.69614 | -3.74058 | 1.97102 |
| H | 3.69377 | -2.18453 | 1.16302 | H | 3.69895 | -2.18041 | 1.16361 |
| C | 0.00386 | 2.16804 | 2.94846 | C | 0.00183 | 2.16951 | 2.9466 |
| C | 2.33598 | -0.17682 | -0.65732 | C | 2.33632 | -0.17489 | -0.65825 |
| H | 2.169 | -0.76245 | -1.55408 | H | 2.17002 | -0.76145 | -1.55453 |
| C | 3.62523 | 0.17126 | -0.25958 | C | 3.62513 | 0.17486 | -0.26081 |
| C | 2.70047 | 1.28342 | 1.66963 | C | 2.69924 | 1.28861 | 1.66695 |
| H | 2.80465 | 1.84076 | 2.58827 | H | 2.80278 | 1.84762 | 2.58464 |
| C | 4.81068 | -0.22586 | -1.05888 | C | 4.81114 | -0.22169 | -1.05963 |
| C | 3.80463 | 0.91285 | 0.96062 | C | 3.80376 | 0.91857 | 0.95827 |
| H | 4.80106 | 1.16346 | 1.30449 | H | 4.79995 | 1.17123 | 1.30137 |
| C | 4.30112 | -3.81701 | -0.10751 | C | 4.30754 | -3.81357 | -0.10538 |
| H | 4.09669 | -3.3015 | -1.05012 | H | 4.10303 | -3.29882 | -1.0484 |
| H | 4.09497 | -4.88264 | -0.25595 | H | 4.10199 | -4.87942 | -0.25311 |
| H | 5.36746 | -3.70267 | 0.10655 | H | 5.37376 | -3.69848 | 0.10882 |
| C | 1.9864 | 4.06857 | -0.55252 | C | 1.97763 | 4.07277 | -0.55307 |
| H | 2.40177 | 4.6571 | -1.38118 | H | 2.39196 | 4.66193 | -1.38179 |
| H | 2.59417 | 3.15889 | -0.48331 | H | 2.58695 | 3.16412 | -0.48378 |
| C | 2.10512 | 4.85335 | 0.75569 | C | 2.09518 | 4.85783 | 0.75507 |
| H | 1.50534 | 5.76999 | 0.72425 | H | 1.4941 | 5.7736 | 0.72361 |
| H | 3.14314 | 5.13987 | 0.95134 | H | 3.13283 | 5.14582 | 0.95061 |
| H | 1.75891 | 4.256 | 1.6044 | H | 1.74997 | 4.26003 | 1.60388 |

**Table S7**. Cartesian coordinates for optimized structures of **tBuCz-*m*-CF3**.

| Ground state of **tBuCz-*m*-CF3** (S_0_) | | | | Excited state of **tBuCz-*m*-CF3** (T_1_) | | | |
| --- | --- | --- | --- | --- | --- | --- | --- |
| Ir | 0.88446 | 0.30442 | -0.50059 | Ir | 0.84123 | 0.24077 | -0.58077 |
| F | -0.36547 | -1.84436 | 4.36931 | F | -0.38967 | -1.1354 | 4.57217 |
| F | 4.1811 | -1.42087 | 3.34007 | F | 4.17471 | -0.88776 | 3.48318 |
| N | -0.84272 | -1.88293 | -1.35969 | N | -0.84186 | -2.08781 | -1.01291 |
| N | 3.04615 | 0.29782 | -0.39675 | N | 3.00063 | 0.29976 | -0.49855 |
| N | -0.95623 | 2.31538 | 0.53276 | N | -0.97377 | 2.393 | 0.14435 |
| N | 0.96747 | 3.25316 | 0.81271 | N | 0.9564 | 3.34938 | 0.29171 |
| N | 1.12893 | -2.66649 | -1.75346 | N | 1.14533 | -2.91462 | -1.18749 |
| N | 1.06192 | 1.5911 | -3.45199 | N | 0.93527 | 1.02722 | -3.71383 |
| C | 0.39897 | 2.12098 | 0.34367 | C | 0.38794 | 2.17314 | -0.01753 |
| C | 1.20383 | -0.54363 | 1.3496 | C | 1.20719 | -0.30311 | 1.39397 |
| C | -1.76007 | -0.91571 | -0.87708 | C | -1.75784 | -1.06493 | -0.70324 |
| C | 0.50049 | -1.56627 | -1.28596 | C | 0.50591 | -1.75069 | -0.99334 |
| C | -1.81905 | 1.27216 | 0.1132 | C | -1.82912 | 1.30367 | -0.10985 |
| C | -3.14735 | -1.01361 | -0.81246 | C | -3.13055 | -1.15459 | -0.61009 |
| H | -3.69327 | -1.87515 | -1.17911 | H | -3.67058 | -2.06314 | -0.84057 |
| C | -1.08909 | 0.21878 | -0.43061 | C | -1.08466 | 0.1565 | -0.47102 |
| C | 2.57746 | -0.7321 | 1.69463 | C | 2.58812 | -0.44437 | 1.73842 |
| C | 2.41339 | 3.45869 | 0.88178 | C | 2.40863 | 3.55436 | 0.33705 |
| H | 2.60265 | 4.53412 | 0.82405 | H | 2.60088 | 4.609 | 0.1237 |
| H | 2.84307 | 2.99633 | -0.00794 | H | 2.83813 | 2.95502 | -0.46587 |
| C | 3.57166 | -0.30249 | 0.71199 | C | 3.56853 | -0.17544 | 0.70194 |
| C | 0.91673 | 1.13331 | -2.38341 | C | 0.88677 | 0.74383 | -2.57824 |
| C | -3.20511 | 1.21398 | 0.22828 | C | -3.2027 | 1.25928 | -0.001 |
| H | -3.79062 | 2.00841 | 0.67655 | H | -3.78872 | 2.10806 | 0.32646 |
| C | 0.00352 | 4.13583 | 1.29796 | C | -0.00643 | 4.29493 | 0.65614 |
| H | 0.26676 | 5.09476 | 1.71387 | H | 0.26344 | 5.29896 | 0.94056 |
| C | 0.21185 | -3.64641 | -2.13128 | C | 0.239 | -3.96701 | -1.34737 |
| H | 0.52622 | -4.59442 | -2.5366 | H | 0.56943 | -4.97773 | -1.52343 |
| C | 1.92201 | -1.65398 | 3.86993 | C | 1.91108 | -1.03046 | 4.03734 |
| H | 2.19529 | -2.06394 | 4.83331 | H | 2.19237 | -1.2934 | 5.04939 |
| C | 3.11649 | -2.08765 | -3.13387 | C | 3.24428 | -2.6152 | -2.5083 |
| H | 2.81449 | -1.03552 | -3.10502 | H | 2.96616 | -1.57467 | -2.70223 |
| H | 2.64034 | -2.52106 | -4.02208 | H | 2.84161 | -3.22034 | -3.3306 |
| C | -1.02915 | -3.15491 | -1.87952 | C | -1.01044 | -3.451 | -1.2332 |
| H | -2.00335 | -3.59273 | -2.01964 | H | -1.97813 | -3.92167 | -1.28554 |
| C | 0.59278 | -1.47632 | 3.49295 | C | 0.58301 | -0.9048 | 3.65025 |
| C | -3.8554 | 0.06271 | -0.2496 | C | -3.84967 | 0.01973 | -0.25937 |
| C | 0.22368 | -0.93326 | 2.27153 | C | 0.22268 | -0.54178 | 2.35615 |
| H | -0.83036 | -0.81238 | 2.04826 | H | -0.83162 | -0.44859 | 2.11918 |
| C | 2.58137 | -2.78383 | -1.88409 | C | 2.60749 | -3.04933 | -1.1909 |
| H | 2.82275 | -3.85038 | -1.89364 | H | 2.83178 | -4.09589 | -0.9698 |
| H | 3.02574 | -2.3537 | -0.98493 | H | 2.98875 | -2.43889 | -0.37036 |
| C | 3.01909 | 2.84898 | 2.14705 | C | 2.99685 | 3.12965 | 1.68252 |
| H | 2.64348 | 3.38344 | 3.02873 | H | 2.61251 | 3.78401 | 2.47573 |
| H | 2.66509 | 1.81745 | 2.23241 | H | 2.64707 | 2.117 | 1.90138 |
| C | -1.20784 | 3.54809 | 1.11665 | C | -1.22089 | 3.69765 | 0.55908 |
| H | -2.20248 | 3.89771 | 1.33786 | H | -2.21242 | 4.07852 | 0.73813 |
| C | 4.63947 | -2.21039 | -3.24245 | C | 4.76929 | -2.74263 | -2.44306 |
| H | 4.91244 | -3.26669 | -3.36628 | H | 5.04163 | -3.79808 | -2.30628 |
| H | 5.10432 | -1.88557 | -2.30385 | H | 5.13694 | -2.20725 | -1.56227 |
| C | 2.88437 | -1.27278 | 2.9536 | C | 2.87515 | -0.7944 | 3.07057 |
| C | 3.85493 | 0.79477 | -1.34991 | C | 3.83903 | 0.70757 | -1.49398 |
| H | 3.35299 | 1.26469 | -2.18748 | H | 3.34953 | 1.09434 | -2.37986 |
| C | 5.23477 | 0.69801 | -1.28084 | C | 5.20258 | 0.63139 | -1.42058 |
| C | 4.96418 | -0.46082 | 0.81659 | C | 4.95009 | -0.32277 | 0.80204 |
| H | 5.3869 | -0.96227 | 1.67183 | H | 5.37164 | -0.73196 | 1.70745 |
| C | 5.78761 | 0.03551 | -0.18279 | C | 5.79464 | 0.06405 | -0.24053 |
| C | 5.21153 | -1.38923 | -4.39916 | C | 5.46537 | -2.18321 | -3.68378 |
| H | 4.97516 | -0.32678 | -4.27699 | H | 5.20563 | -1.12884 | -3.82139 |
| H | 4.79176 | -1.70979 | -5.35863 | H | 5.1737 | -2.72644 | -4.58984 |
| H | 6.2999 | -1.48842 | -4.45708 | H | 6.55312 | -2.24616 | -3.58188 |
| C | 4.54984 | 2.86941 | 2.1252 | C | 4.52775 | 3.14582 | 1.67066 |
| H | 4.90662 | 3.9074 | 2.10274 | H | 4.88608 | 4.17014 | 1.5014 |
| H | 4.89982 | 2.40637 | 1.19482 | H | 4.88338 | 2.54332 | 0.82755 |
| C | 5.16361 | 2.13527 | 3.31968 | C | 5.12259 | 2.59263 | 2.96759 |
| H | 4.86811 | 2.60315 | 4.26516 | H | 4.79521 | 3.17597 | 3.83607 |
| H | 6.25691 | 2.14183 | 3.2693 | H | 6.2168 | 2.61628 | 2.94112 |
| H | 4.83901 | 1.09062 | 3.35188 | H | 4.81625 | 1.55405 | 3.12354 |
| C | -5.98384 | -1.02495 | 0.50224 | C | -5.9716 | -1.09924 | 0.43043 |
| C | -6.1785 | 0.89941 | -0.68255 | C | -6.14651 | 0.9255 | -0.62259 |
| C | -5.51776 | -2.13745 | 1.20655 | C | -5.52482 | -2.23474 | 1.10862 |
| C | -7.37078 | -0.75097 | 0.39032 | C | -7.34953 | -0.7952 | 0.33706 |
| C | -5.94537 | 2.05033 | -1.43879 | C | -5.91306 | 2.09442 | -1.34911 |
| C | -7.4949 | 0.47849 | -0.36555 | C | -7.46079 | 0.49022 | -0.33472 |
| C | -6.46303 | -2.98067 | 1.78255 | C | -6.48762 | -3.09104 | 1.6387 |
| H | -4.45601 | -2.33432 | 1.30612 | H | -4.47128 | -2.44483 | 1.24322 |
| C | -8.29568 | -1.62047 | 0.98066 | C | -8.29183 | -1.66519 | 0.88159 |
| C | -7.05183 | 2.78202 | -1.85872 | C | -7.02018 | 2.84965 | -1.73005 |
| H | -4.93808 | 2.35883 | -1.69596 | H | -4.9147 | 2.40469 | -1.6306 |
| C | -8.58646 | 1.23842 | -0.80291 | C | -8.55111 | 1.26095 | -0.7329 |
| C | -7.85155 | -2.74391 | 1.67786 | C | -7.86844 | -2.83389 | 1.52713 |
| H | -6.12115 | -3.8518 | 2.33555 | H | -6.15999 | -3.98216 | 2.16568 |
| H | -9.36066 | -1.41908 | 0.89964 | H | -9.3509 | -1.43414 | 0.81736 |
| C | -8.37538 | 2.39766 | -1.54901 | C | -8.33945 | 2.46039 | -1.42462 |
| H | -6.89276 | 3.6804 | -2.44953 | H | -6.85913 | 3.76455 | -2.29225 |
| H | -9.59928 | 0.92393 | -0.56491 | H | -9.56276 | 0.92882 | -0.51968 |
| N | -5.26795 | -0.01897 | -0.15234 | N | -5.23522 | -0.04684 | -0.15595 |
| C | -9.53504 | 3.23891 | -2.0268 | C | -9.50013 | 3.32835 | -1.83986 |
| H | -9.52116 | 3.35871 | -3.11616 | H | -9.37728 | 3.69228 | -2.86461 |
| H | -10.49221 | 2.78717 | -1.75248 | H | -10.44609 | 2.7846 | -1.77943 |
| H | -9.5056 | 4.24579 | -1.59374 | H | -9.58096 | 4.2096 | -1.19225 |
| C | -8.82932 | -3.70164 | 2.31628 | C | -8.86839 | -3.8079 | 2.09667 |
| H | -9.85919 | -3.35391 | 2.19837 | H | -9.86214 | -3.35987 | 2.1733 |
| H | -8.76223 | -4.70074 | 1.86956 | H | -8.95277 | -4.69794 | 1.46166 |
| H | -8.63545 | -3.81698 | 3.38883 | H | -8.5673 | -4.14996 | 3.09168 |
| C | 7.27046 | -0.20787 | -0.12996 | C | 7.23982 | -0.20621 | -0.2062 |
| F | 7.5979 | -1.30234 | -0.85742 | F | 7.61664 | -1.25106 | -1.02292 |
| F | 7.70863 | -0.41592 | 1.12748 | F | 7.6978 | -0.52422 | 1.03057 |
| F | 7.9639 | 0.83003 | -0.64196 | F | 7.98513 | 0.85055 | -0.64661 |
| H | 5.85386 | 1.10958 | -2.06764 | H | 5.81286 | 0.97628 | -2.24626 |

**Table S8**. Cartesian coordinates for optimized structures of **tBuCz-*p*-CF3**.

| Ground state of **tBuCz-*p*-CF3** (S_0_) | | | | Excited state of **tBuCz-*p*-CF3** (T_1_) | | | |
| --- | --- | --- | --- | --- | --- | --- | --- |
| Ir | -0.98032 | -0.18441 | -0.28907 | Ir | 0.97841 | 0.13689 | -0.3111 |
| F | 0.8041 | 2.65599 | 4.04166 | F | -0.8352 | -1.40541 | 4.6001 |
| F | -3.85784 | 2.25891 | 3.54558 | F | 3.8084 | -1.34722 | 3.9841 |
| N | 0.76682 | 1.79827 | -1.47917 | N | -0.7403 | -2.07911 | -0.9988 |
| N | -3.12811 | -0.04327 | -0.03847 | N | 3.11021 | 0.04639 | -0.0311 |
| N | 0.78276 | -2.16388 | 0.91163 | N | -0.78889 | 2.31979 | 0.3761 |
| N | -1.16956 | -2.91649 | 1.44034 | N | 1.15491 | 3.17249 | 0.712 |
| F | -5.81448 | -1.85473 | -2.49934 | F | 5.78681 | 1.09728 | -2.8915 |
| N | -1.20129 | 2.60899 | -1.85479 | N | 1.2213 | -2.94031 | -1.1724 |
| N | -1.45387 | -1.80575 | -3.0388 | N | 1.42681 | 1.10369 | -3.3406 |
| C | -0.57431 | -1.90715 | 0.785 | C | 0.56311 | 2.04099 | 0.313 |
| F | -7.26862 | -0.27749 | -2.07858 | F | 7.21771 | -0.33292 | -2.1 |
| C | -1.12464 | 0.95371 | 1.44869 | C | 1.10661 | -0.51841 | 1.6474 |
| C | 1.6582 | 0.8562 | -0.9318 | C | -1.635 | -1.0466 | -0.6961 |
| C | -0.58851 | 1.54983 | -1.30522 | C | 0.6058 | -1.78871 | -0.8835 |
| C | 1.66539 | -1.24532 | 0.30584 | C | -1.66129 | 1.2896 | 0.0069 |
| C | 3.04028 | 0.88427 | -0.94017 | C | -3.0098 | -1.0858 | -0.6974 |
| H | 3.606 | 1.65824 | -1.44255 | H | -3.5685 | -1.9602 | -1.0096 |
| C | 0.95188 | -0.20666 | -0.32836 | C | -0.93619 | 0.13449 | -0.3614 |
| C | -2.46934 | 1.2618 | 1.85487 | C | 2.44011 | -0.74791 | 2.115 |
| C | -2.6205 | -2.99543 | 1.64458 | C | 2.59611 | 3.30329 | 0.8868 |
| H | -2.88293 | -4.05093 | 1.75617 | H | 2.85852 | 4.35199 | 0.7189 |
| H | -3.08906 | -2.61966 | 0.735 | H | 3.06601 | 2.70179 | 0.1061 |
| F | -7.18 | -2.02865 | -0.80802 | F | 7.17221 | 1.67378 | -1.3285 |
| C | -3.54179 | 0.7909 | 1.03303 | C | 3.51751 | -0.49472 | 1.2053 |
| C | -1.26221 | -1.22412 | -2.04081 | C | 1.24911 | 0.75299 | -2.2381 |
| C | 3.04502 | -1.25306 | 0.32835 | C | -3.03659 | 1.2947 | 0.0192 |
| H | 3.61674 | -2.00835 | 0.8524 | H | -3.61459 | 2.1481 | 0.3544 |
| C | -0.22612 | -3.7901 | 1.98517 | C | 0.21562 | 4.14399 | 1.0352 |
| H | -0.51723 | -4.66028 | 2.55072 | H | 0.50692 | 5.12399 | 1.3805 |
| C | -0.271 | 3.50764 | -2.38317 | C | 0.3022 | -3.93701 | -1.4784 |
| H | -0.57688 | 4.41677 | -2.87485 | H | 0.6146 | -4.93551 | -1.7424 |
| C | -1.54514 | 2.48034 | 3.79598 | C | 1.5048 | -1.39671 | 4.3026 |
| H | -1.71396 | 3.03976 | 4.70774 | H | 1.6708 | -1.71901 | 5.3242 |
| C | -3.2406 | 1.91976 | -3.09244 | C | 3.2346 | -2.64372 | -2.5684 |
| H | -2.95592 | 0.87231 | -2.95117 | H | 2.9754 | -1.58811 | -2.7178 |
| H | -2.78566 | 2.24609 | -4.03675 | H | 2.7497 | -3.20831 | -3.3767 |
| C | 0.96623 | 3.00391 | -2.1433 | C | -0.9349 | -3.39931 | -1.364 |
| H | 1.94355 | 3.39019 | -2.37979 | H | -1.9118 | -3.834 | -1.5037 |
| C | -0.26274 | 2.19984 | 3.33258 | C | 0.2305 | -1.18531 | 3.7976 |
| C | 3.72702 | -0.18129 | -0.30749 | C | -3.70509 | 0.0971 | -0.341 |
| C | -0.04547 | 1.44506 | 2.18047 | C | 0.02461 | -0.74531 | 2.4942 |
| H | 0.97766 | 1.24005 | 1.885 | H | -0.997 | -0.58551 | 2.1612 |
| C | -2.66149 | 2.74127 | -1.94196 | C | 2.6706 | -3.09381 | -1.2294 |
| H | -2.8832 | 3.80551 | -2.05937 | H | 2.8932 | -4.14781 | -1.0378 |
| H | -3.07253 | 2.41043 | -0.98644 | H | 3.0914 | -2.50222 | -0.4127 |
| C | -3.06163 | -2.1665 | 2.85034 | C | 3.03601 | 2.81969 | 2.2607 |
| H | -2.62686 | -2.5893 | 3.76567 | H | 2.60041 | 3.46459 | 3.0364 |
| H | -2.65387 | -1.15809 | 2.73708 | H | 2.63081 | 1.81239 | 2.411 |
| C | 1.00275 | -3.32159 | 1.64893 | C | -1.01039 | 3.61179 | 0.8192 |
| H | 1.98687 | -3.70633 | 1.85866 | H | -1.99579 | 4.0349 | 0.9324 |
| C | -4.76471 | 2.04173 | -3.17119 | C | 4.7459 | -2.82572 | -2.638 |
| H | -5.04008 | 3.08897 | -3.35682 | H | 4.9905 | -3.89202 | -2.5337 |
| H | -5.19925 | 1.76986 | -2.20295 | H | 5.2121 | -2.31062 | -1.7889 |
| C | -2.60823 | 2.00388 | 3.05313 | C | 2.571 | -1.16981 | 3.454 |
| C | -4.05386 | -0.64578 | -0.79768 | C | 4.03501 | 0.39938 | -0.9276 |
| H | -3.67635 | -1.28102 | -1.58966 | H | 3.65621 | 0.81668 | -1.8545 |
| C | -5.41323 | -0.46167 | -0.63157 | C | 5.39141 | 0.24248 | -0.7336 |
| C | -4.92963 | 1.04272 | 1.19587 | C | 4.89801 | -0.71502 | 1.423 |
| H | -5.24046 | 1.72083 | 1.97741 | H | 5.2087 | -1.17152 | 2.3533 |
| C | -6.39224 | -1.14941 | -1.49566 | C | 6.36451 | 0.66578 | -1.7516 |
| C | -5.86602 | 0.44346 | 0.39305 | C | 5.83841 | -0.36472 | 0.4891 |
| H | -6.92319 | 0.64718 | 0.51742 | H | 6.89411 | -0.54552 | 0.6638 |
| C | -5.35971 | 1.14478 | -4.25842 | C | 5.3309 | -2.28252 | -3.9347 |
| H | -5.1275 | 0.09415 | -4.06294 | H | 5.1405 | -1.20762 | -4.0238 |
| H | -4.95979 | 1.4009 | -5.24604 | H | 4.8898 | -2.77812 | -4.8078 |
| H | -6.44854 | 1.23882 | -4.29589 | H | 6.4152 | -2.42932 | -3.9726 |
| C | -4.58574 | -2.08742 | 2.96984 | C | 4.55291 | 2.77708 | 2.395 |
| H | -4.99789 | -3.09241 | 3.13322 | H | 4.96492 | 3.79068 | 2.2912 |
| H | -5.00103 | -1.72835 | 2.02175 | H | 4.96721 | 2.18118 | 1.5716 |
| C | -5.02632 | -1.15238 | 4.09818 | C | 4.99051 | 2.16918 | 3.7211 |
| H | -4.63882 | -1.48888 | 5.06692 | H | 4.59551 | 2.74048 | 4.5702 |
| H | -6.11763 | -1.10947 | 4.17084 | H | 6.08241 | 2.15108 | 3.8083 |
| H | -4.66296 | -0.13509 | 3.92596 | H | 4.63041 | 1.13828 | 3.8118 |
| C | 5.94155 | 0.94143 | -0.0287 | C | -5.8946 | -0.9624 | 0.1526 |
| C | 5.95463 | -1.28436 | -0.54473 | C | -5.92569 | 1.10091 | -0.8096 |
| C | 5.59116 | 2.23766 | 0.35307 | C | -5.5335 | -2.1375 | 0.8087 |
| C | 7.2959 | 0.54051 | -0.10765 | C | -7.2473 | -0.60479 | -0.0153 |
| C | 5.62033 | -2.58285 | -0.93326 | C | -5.60059 | 2.2867 | -1.4654 |
| C | 7.3042 | -0.87446 | -0.43758 | C | -7.26709 | 0.70801 | -0.6306 |
| C | 6.623 | 3.139 | 0.60309 | C | -6.5561 | -2.97639 | 1.2347 |
| H | 4.5572 | 2.53659 | 0.47392 | H | -4.4982 | -2.3909 | 1.0076 |
| C | 8.30911 | 1.46363 | 0.15187 | C | -8.2519 | -1.45889 | 0.4272 |
| C | 6.66303 | -3.47776 | -1.16027 | C | -6.64829 | 3.09911 | -1.8813 |
| H | 4.59101 | -2.88808 | -1.0765 | H | -4.57369 | 2.568 | -1.6704 |
| C | 8.32861 | -1.79108 | -0.67532 | C | -8.29729 | 1.53611 | -1.0635 |
| C | 7.9816 | 2.77852 | 0.49807 | C | -7.9141 | -2.66559 | 1.0444 |
| H | 6.3707 | 4.15299 | 0.89954 | H | -6.2946 | -3.89999 | 1.7441 |
| H | 9.3508 | 1.16104 | 0.09622 | H | -9.2967 | -1.18509 | 0.3053 |
| C | 8.01681 | -3.10831 | -1.02725 | C | -7.99619 | 2.75201 | -1.6815 |
| H | 6.42358 | -4.49344 | -1.46155 | H | -6.41539 | 4.03061 | -2.3905 |
| H | 9.36694 | -1.48173 | -0.59825 | H | -9.33349 | 1.23481 | -0.9334 |
| N | 5.12276 | -0.17447 | -0.29487 | N | -5.08909 | 0.0797 | -0.3328 |
| C | 9.10464 | -4.12588 | -1.26444 | C | -9.08809 | 3.67891 | -2.1326 |
| H | 8.92741 | -4.69028 | -2.1856 | H | -8.89689 | 4.05711 | -3.1426 |
| H | 10.08556 | -3.65001 | -1.34059 | H | -10.06109 | 3.17971 | -2.132 |
| H | 9.15042 | -4.85291 | -0.44481 | H | -9.16018 | 4.55041 | -1.4702 |
| C | 9.05767 | 3.80301 | 0.75795 | C | -8.978 | -3.62079 | 1.5033 |
| H | 10.03976 | 3.33332 | 0.85463 | H | -9.96 | -3.14029 | 1.5327 |
| H | 9.11607 | 4.53028 | -0.06065 | H | -9.049 | -4.48169 | 0.8268 |
| H | 8.85756 | 4.36623 | 1.67515 | H | -8.7572 | -4.01299 | 2.5017 |

Photoluminescence Properties


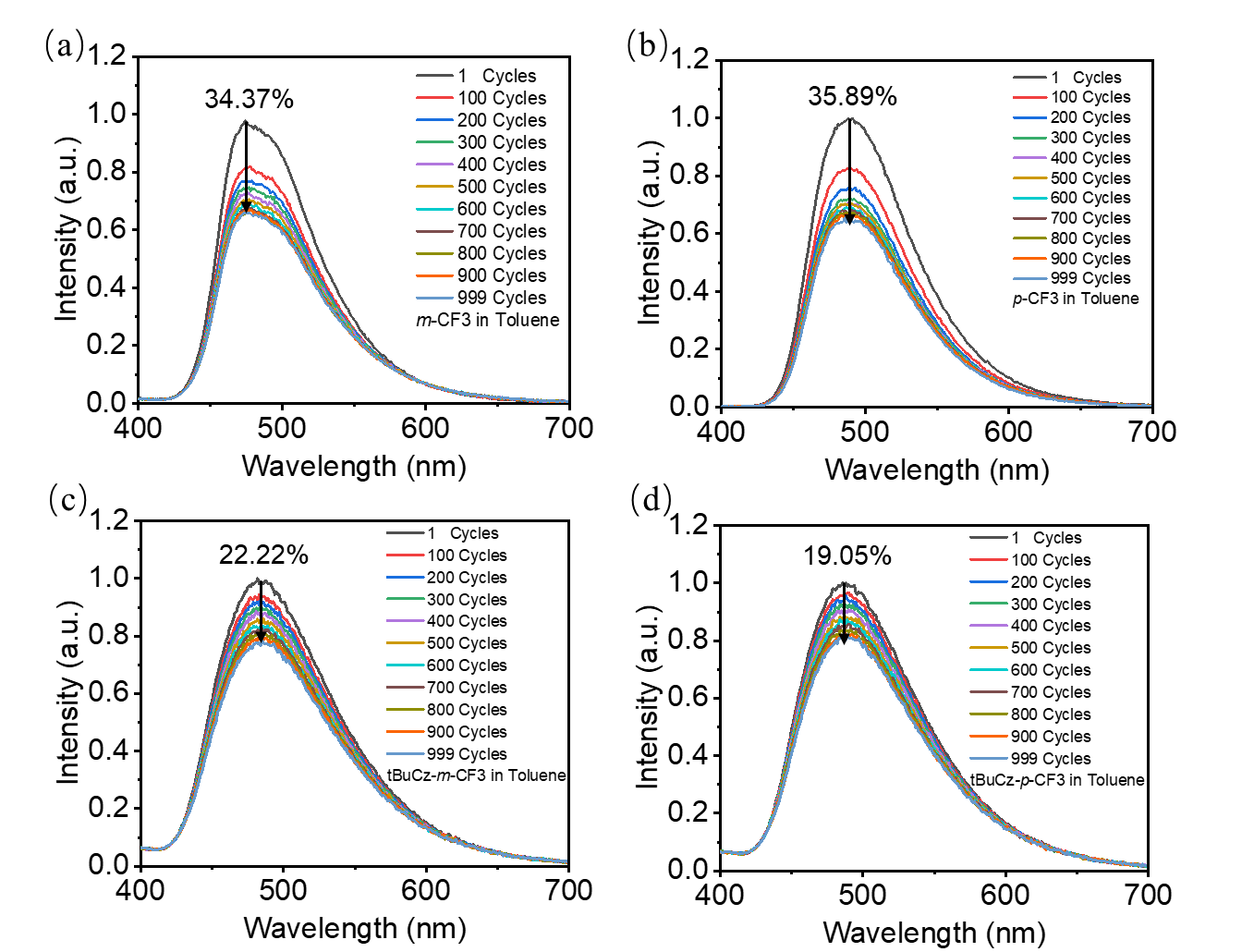


**Fig. S14** Multi-scan of PL spectra (a) ***m*-CF3**, (b) ***p*-CF3**, (c) **tBuCz-*m*-CF3** and (d) **tBuCz-*p*-CF3** at 2 10^-5^ M in toluene under irradiation of 150 W xenon lamp..


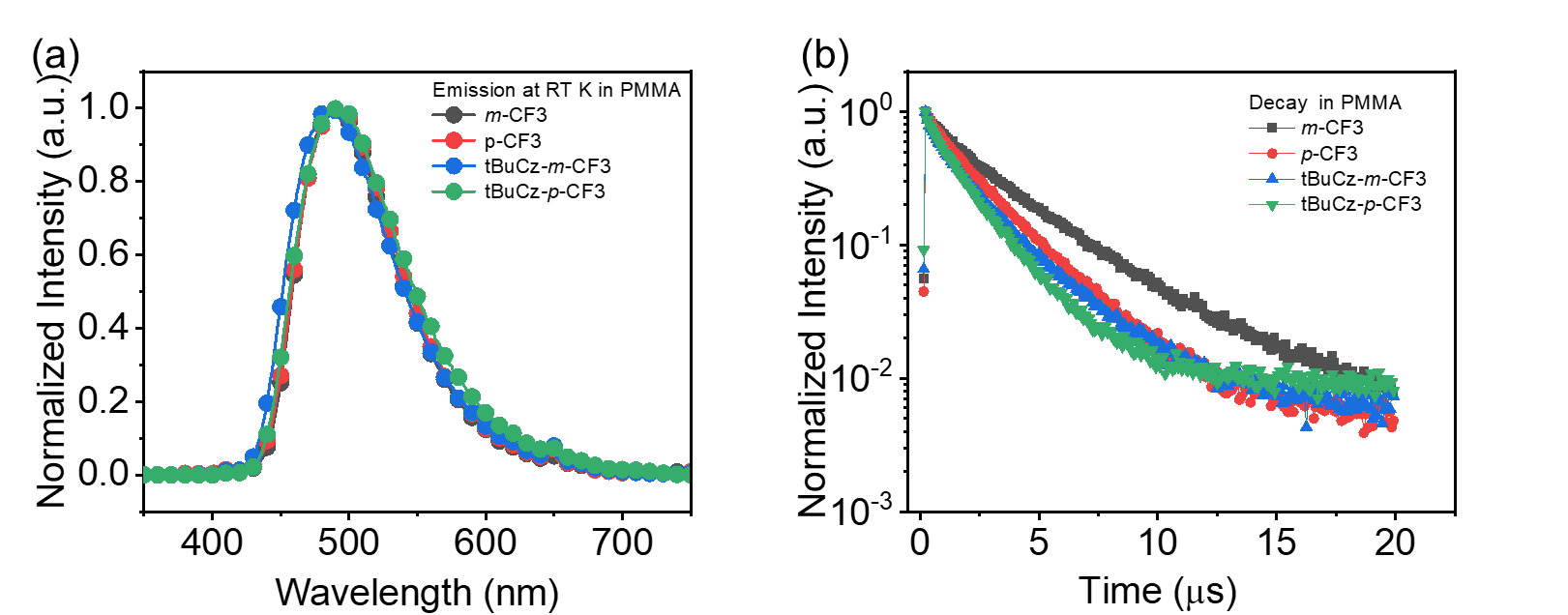


**Fig. S15**. Photophysical properties of the studied Ir(III) emitters in PMMA matrix with concentration of 2 wt% at room temperature. (a) emission spectra. (b) PL decay curves.

**Table S9**. Summary of photophysical properties of the studied Ir(III) emitters in PMMA matrix with concentration of 2 wt% at room temperature.

| 2 wt% in PMMA matrix | λ (nm) | τ (μs) | Φ (%) | k*_r_* (10^5^ s^-1^) | K*_nr_* (10^5^ s^-1^) |
| --- | --- | --- | --- | --- | --- |
| *m*-CF3 | 487 | 3.38 | 82 | 2.43 | 0.53 |
| *p*-CF3 | 493 | 2.36 | 86 | 3.64 | 0.59 |
| tBuCz-*m*-CF3 | 484 | 2.29 | 91 | 3.97 | 0.39 |
| tBuCz-*p*-CF3 | 495 | 1.86 | 94 | 5.05 | 0.32 |

Electroluminescent Performance


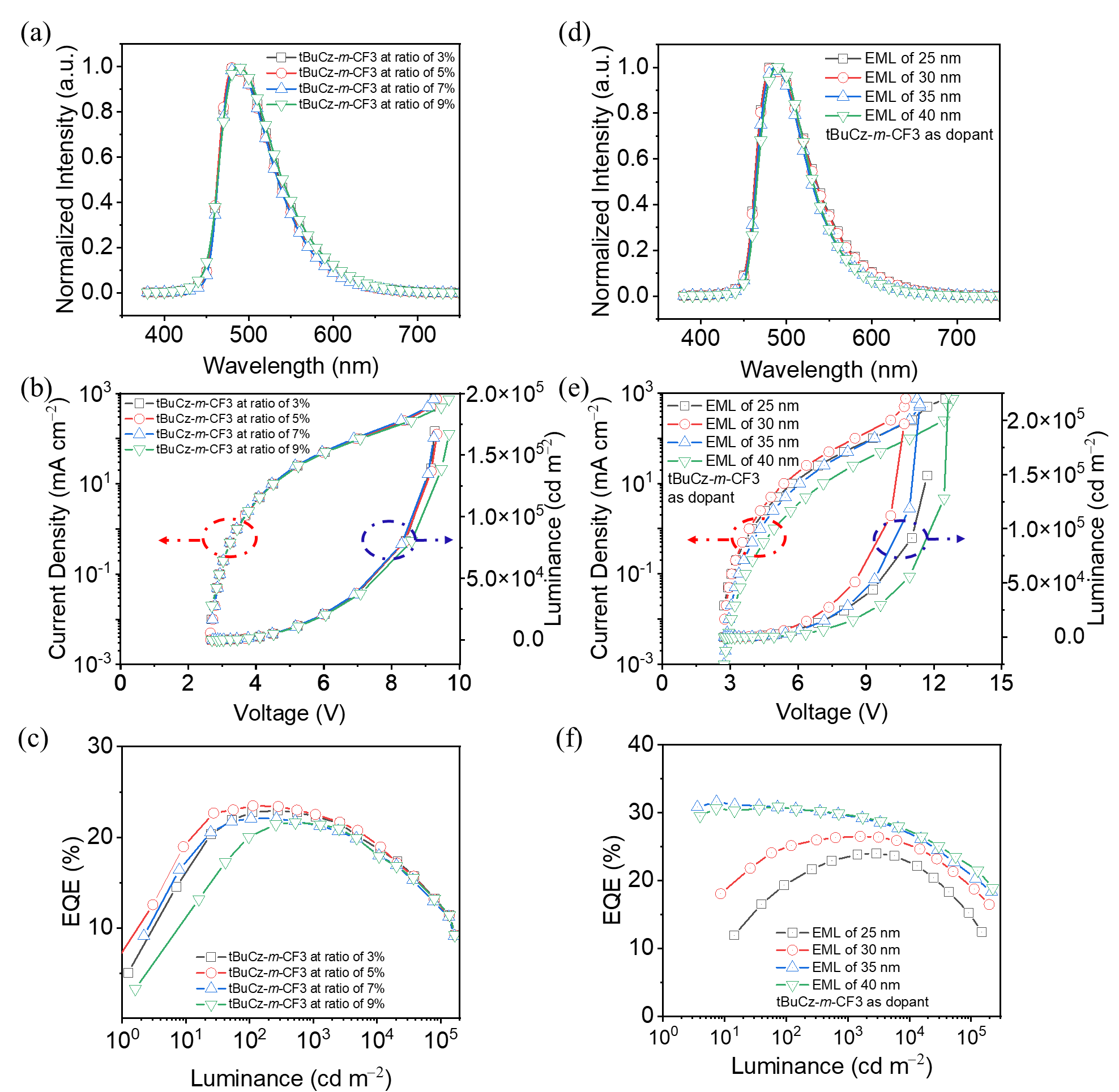


Fig. S16. Electroluminescent performances based on tBuCz-*m*-CF3 doped in EML with varied thickness and doping concentrations.

(a) EL spectra; (b) *J-V-L* curves and (c) EQE versus luminance with varied dopant concentration; (d) EL spectra; (e) *J-V-L* curves and (f) EQE versus luminance with varied EML thickness.

Table S10. OLED performances based on tBuCz-*m*-CF3 with varied EML thickness and doping concentrations.

| **Dopant** | **EQE (%)** | | | | **CE_m_** | **PE_m_** | **CIE** | **L_max_** |
| --- | --- | --- | --- | --- | --- | --- | --- | --- |
|  | Max. | 1000 cd m^-2^ | 10000 cd m^-2^ | 50000 cd m^-2^ | cd A^-1^ | lm W^-1^ | x, y | cd m^-2^ |
| ^a^ 3%  tBuCz-*m*-CF3 | 22.96 | 22.31 | 18.61 | 15.74 | 52.77 | 55.62 | 0.175,0.400 | 169340 |
| 5%  tBuCz-*m*-CF3 | 23.48 | 22.59 | 18.95 | 15.72 | 56.54 | 58.46 | 0.175,0.400 | 166970 |
| 7%  tBuCz-*m*-CF3 | 23.38 | 20.97 | 17.99 | 15.29 | 52.81 | 54.61 | 0.181,0.417 | 134432 |
| 9%  tBuCz-*m*-CF3 | 22.80 | 22.55 | 18.81 | 15.54 | 51.56 | 47.64 | 0.181,0.417 | 131211 |
| ^b^ 25 nm  tBuCz-*m*-CF3 | 24.02 | 22.20 | 18.33 | 15.45 | 57.48 | 35.62 | 0.172,0.407 | 148960 |
| 30 nm  tBuCz-*m*-CF3 | 26.53 | 24.67 | 21.54 | 18.77 | 63.30 | 45.95 | 0.173,0.416 | 196611 |
| 35 nm  tBuCz-*m*-CF3 | 31.62 | 30.19 | 27.76 | 24.00 | 73.82 | 78.81 | 0.175,0.446 | 214255 |
| **40 nm**  **tBuCz-*m*-CF3** | **30.91** | **30.51** | **28.00** | **23.53** | **73.55** | **66.56** | **0.183,0.477** | **224844** |

^a^ The dopant concentration is varied from 3 wt%, 5 wt%, 7 wt% and to 9 wt% with a constant EML thickness of 20 nm; while the doping ratio of SiCzCz is maintained at 30 wt%, while doping ratios of SiTrzCz2 are varied from 67 wt%, 65 wt%, 63 wt% and to 61 wt%, respectively.

^b^ The EML thickness is varied from 25 nm, 30 nm, 35 nm and to 40 nm with a constant doping ratio of **tBuCz-*m*-CF3** maintained at 5 wt%.


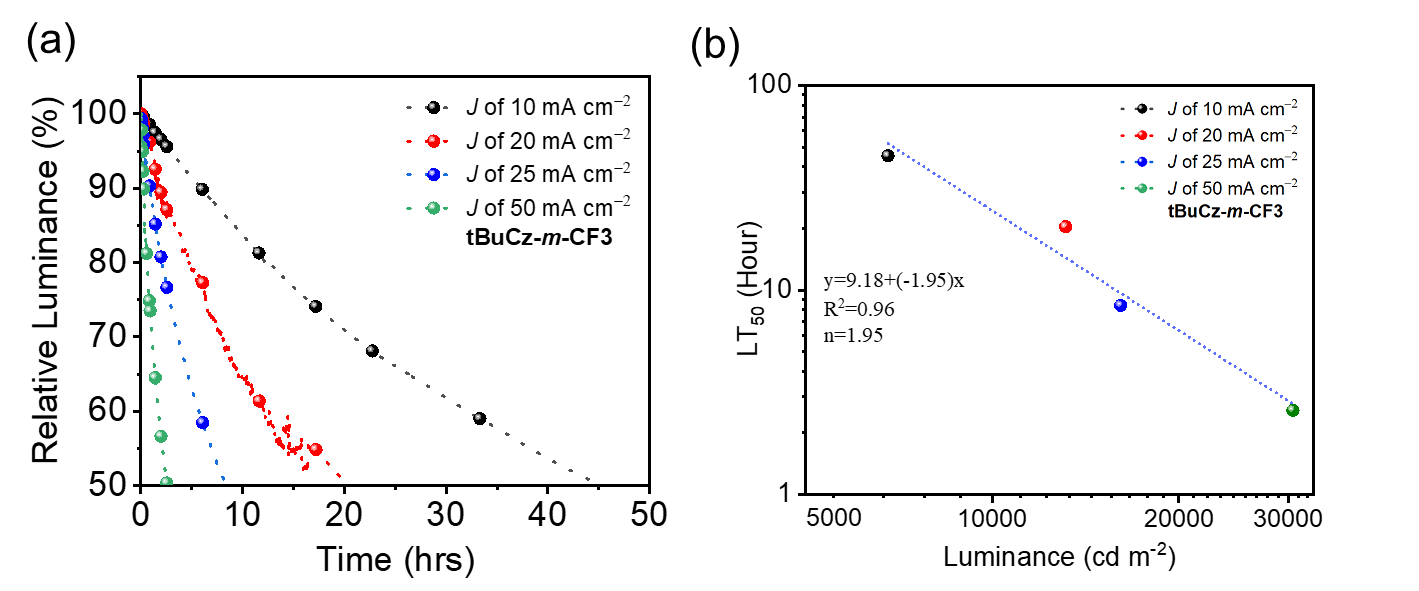


Fig. S17. The measurement of device stability.

(a) Plot of relative luminance versus operational lifetime at various current density; (b) Plots of LT_50_ lifetime versus initial luminance (L_0_) with the fit using the formula LT_50_L_0_n = constant for blue devices based on the emitter of **tBuCz-*m*-CF3**.

**
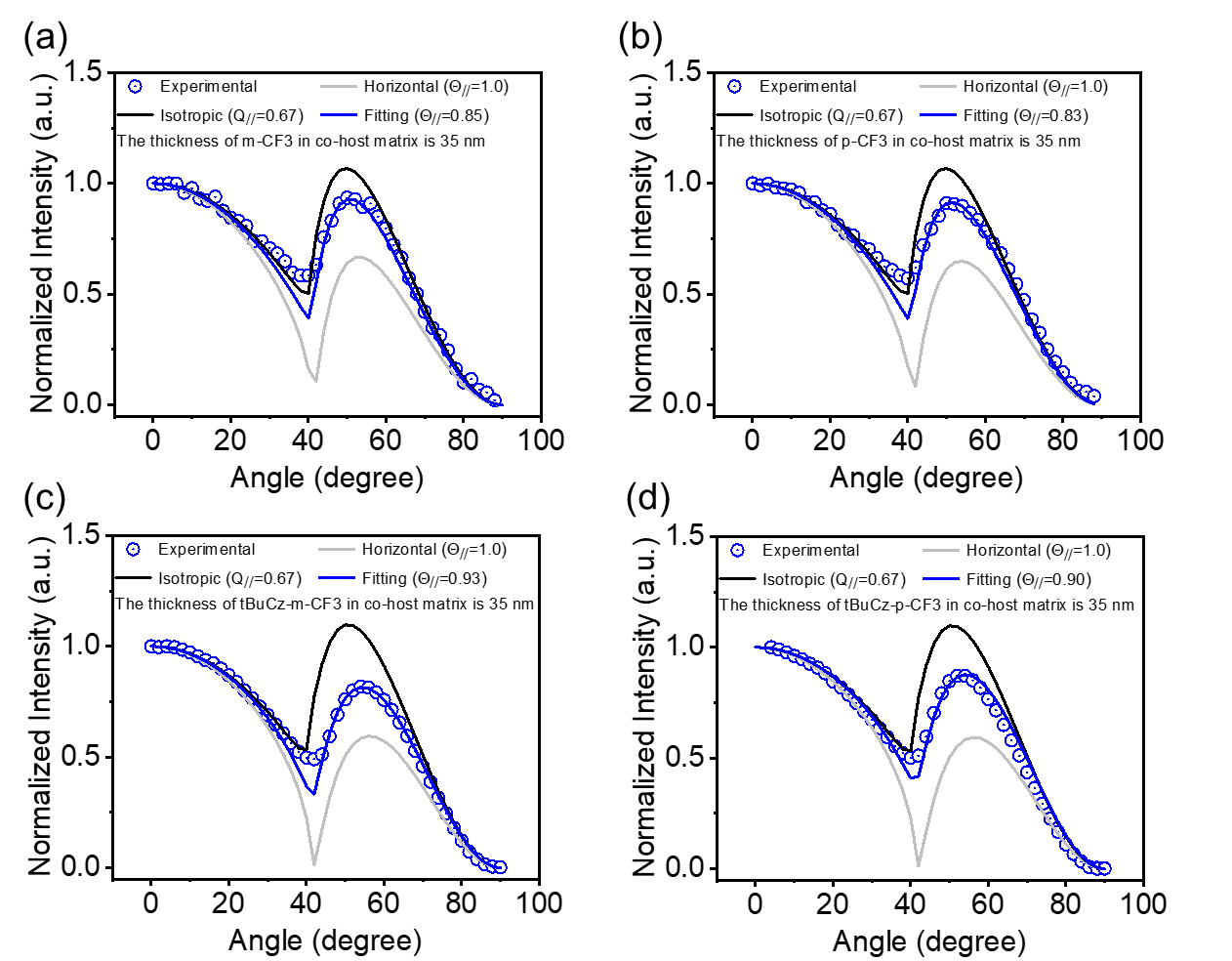
**

**Fig. S18.** Angle dependent PL (ADPL) measurement showing the experimental and fitting result of horizontal dipole ratio (Θ_//_).


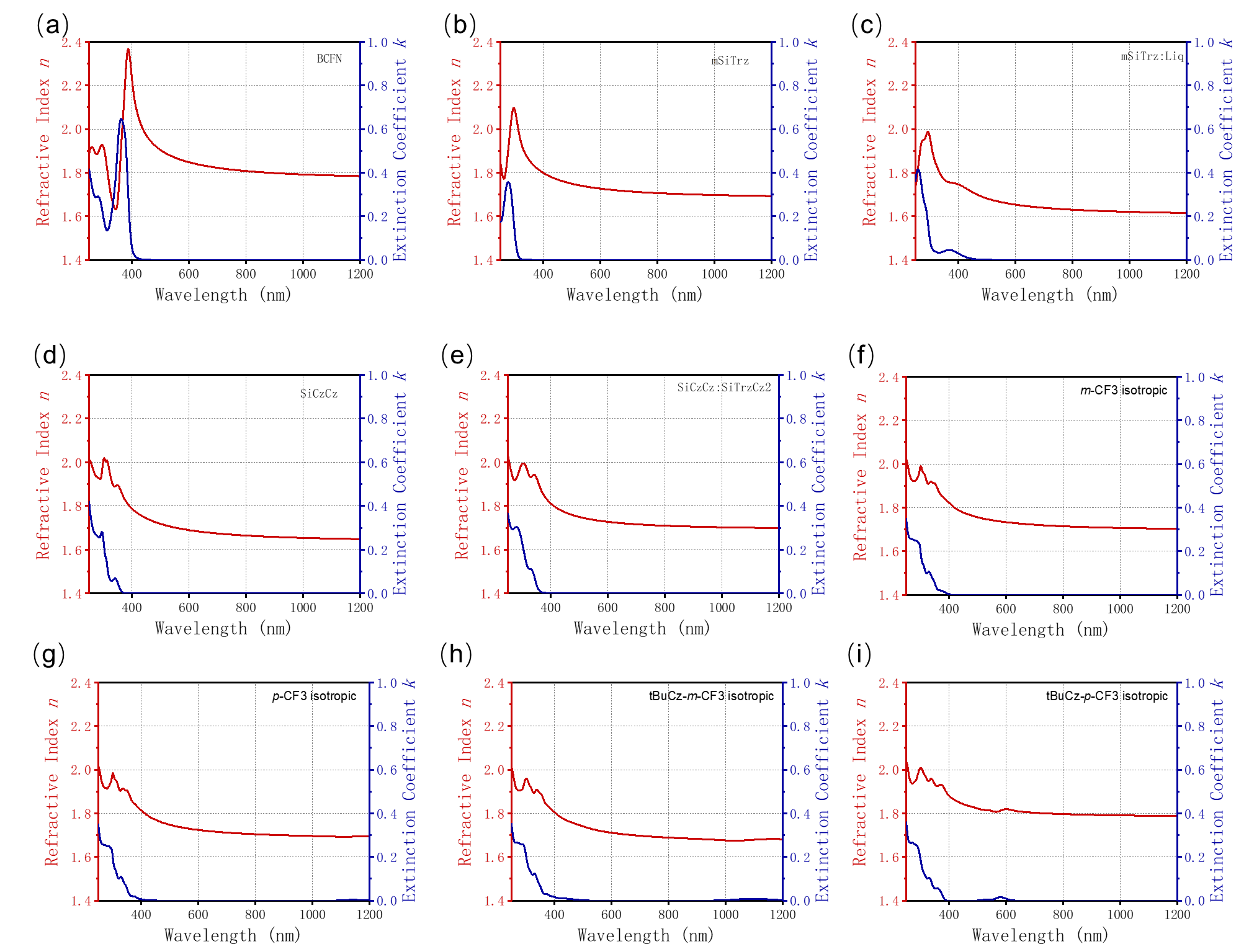


**Fig. S19.** Refractive index of organic materials used in OLED device that were measured by variable angle spectroscopic ellipsometry (VASE). Red line represents refractive index (n) and blue line represents extinction coefficient (k). Ellipsometry data Ψ and Δ were fitted using isotropic general oscillator model provided by CompleteEase® program manufactured by J. A. Woollam.


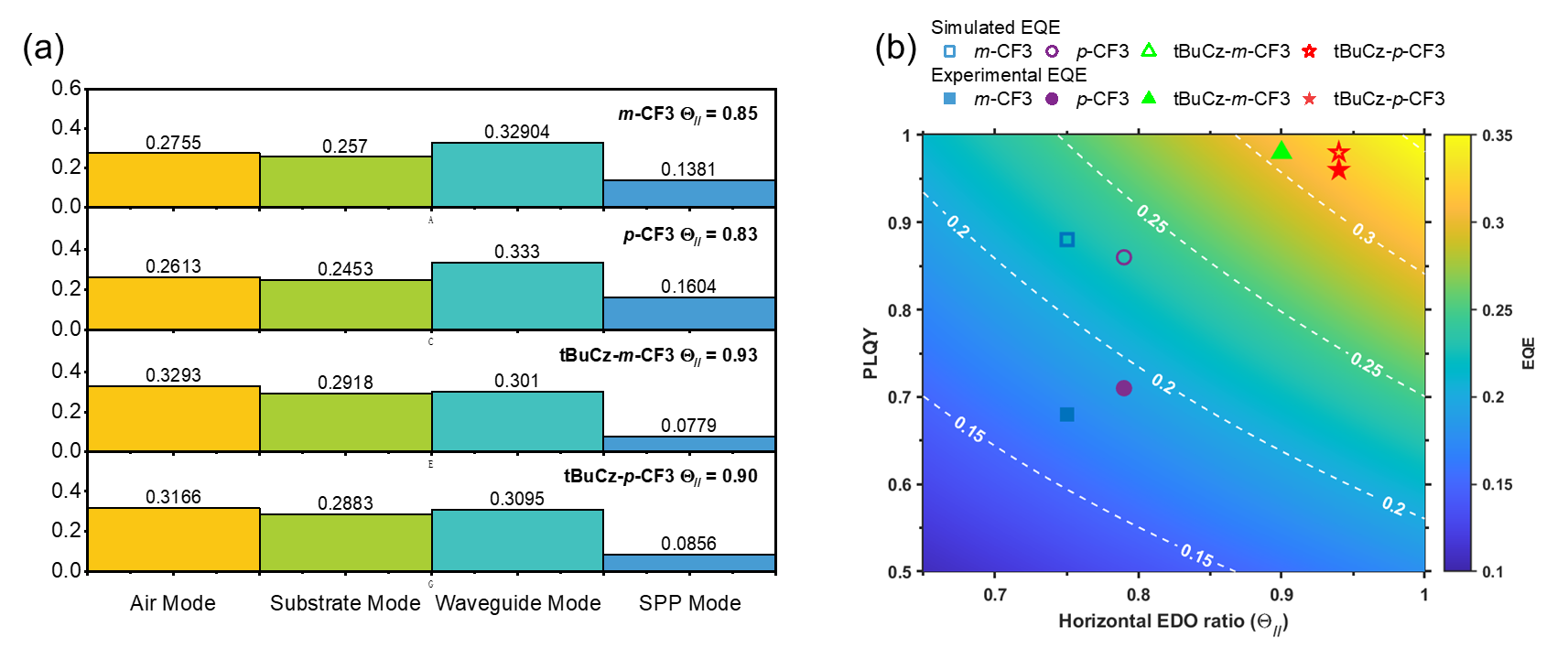


**Fig. S20**. a) Simulated outcoupling efficiency (air mode) and the probability of light dissipated to other modes. b) Simulated maximum achievable EQE values presented as a contour plot, with a color bar representing the estimated EQE values.


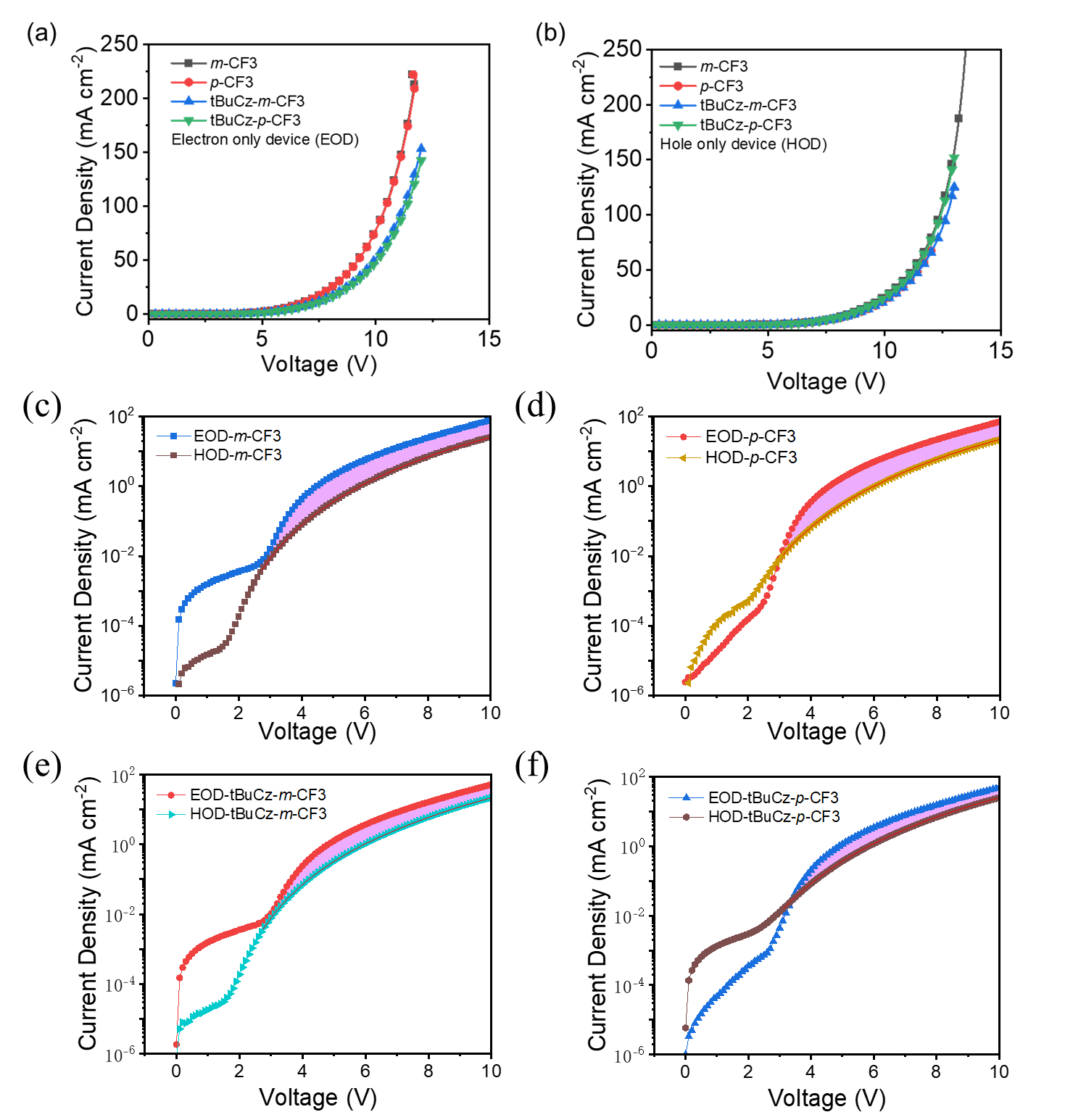


**Figure S21**. Current density-voltage characteristics of the (a) electron only device; and (b) hole-only device for all the Ir(III) complexes; the EOD and HOD curves for (c) ***m*-CF3**, (d) ***p*-CF3**, (e) **tBuCz-*m*-CF3** and (d) **tBuCz-*p*-CF3**.


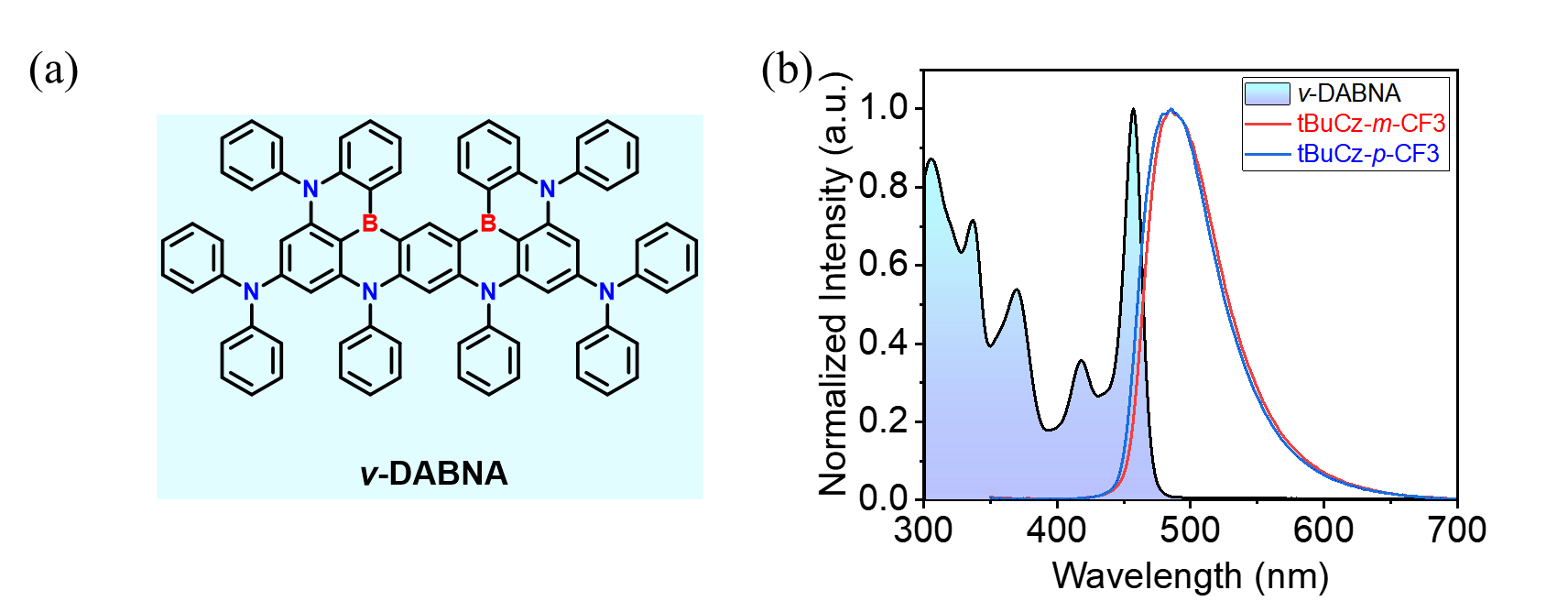


**Figure S22**. (a)The molecular structure of *v*-DABNA; (b) the absorption of *v*-DABNA in toluene and the emissions of **tBuCz-*m*-CF3** and **tBuCz-*p*-CF3** in co-host matrix.


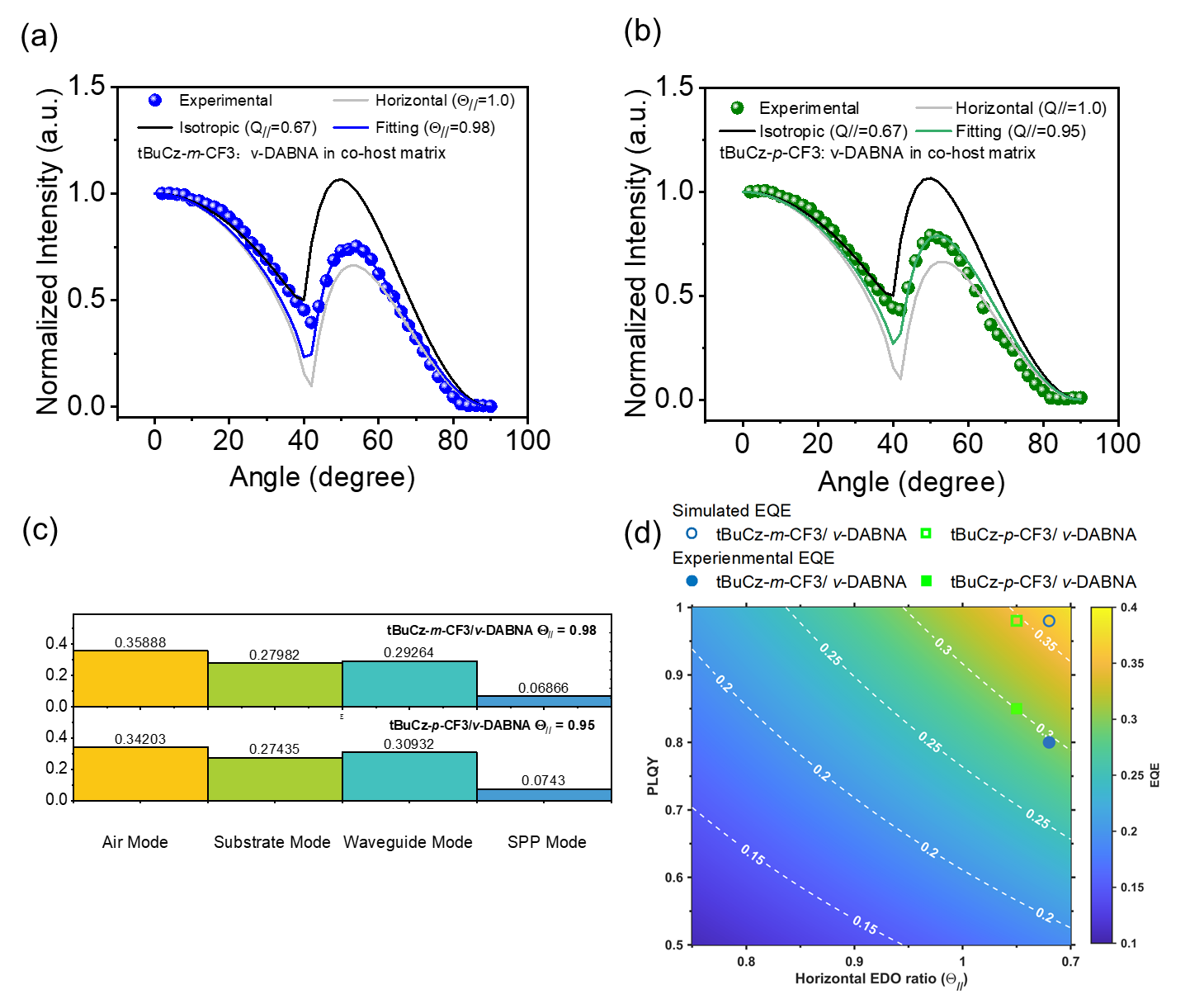


**Figure S23.** Angle dependent PL (ADPL) measurement showing the experimental and fitting result of horizontal dipole ratio (Θ_//_) for (a) tBuCz-*m*-CF3/ v-DABNA and (b) tBuCz-*p*-CF3/ v-DABNA in co-host materix with the thickness of 35 nm; (c) Simulated outcoupling efficiency (air mode) and the probability of light dissipated to other modes. (d) Simulated maximum achievable EQE values presented as a contour plot, with a color bar representing the estimated EQE values.


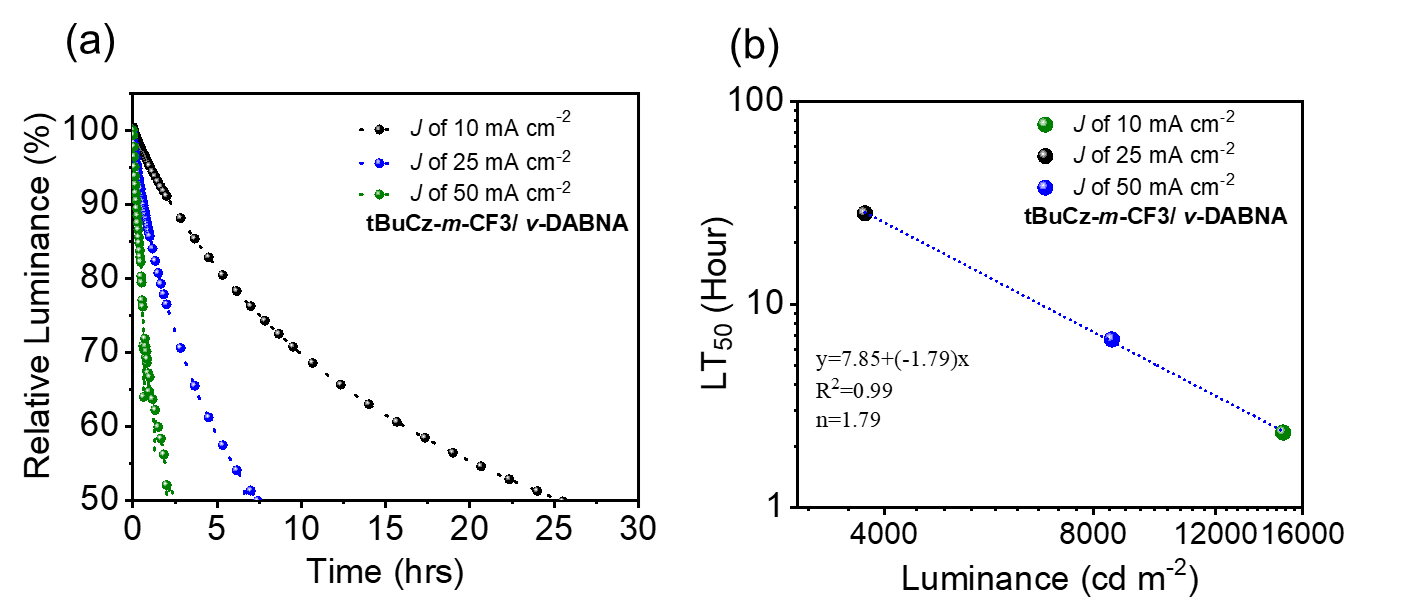


**Figure S24.** The measurement of hyper-OLED stability.

(a) Plot of relative luminance versus operational lifetime at various current density; (b) Plots of LT_50_ lifetime versus initial luminance (L_0_) with the fit using the formula LT_50_L_0_n = constant for hyper-OLEDs based on the emitter of tBuCz-*m*-CF3/ v-DABNA.

Table S11. Reported performances of green to blue-emitting Ph-OLEDs based on Ir(III) phosphors.

| **Samples** | **Dopants** | **EL(nm)** | **EQE(%)** | **reported LT data** | **CIE** | **Ref.** |
| --- | --- | --- | --- | --- | --- | --- |
| **1** | ***m*-CF3** | 492 | 19.05 | LT_50_ = 452 h, (1000 nit) | 0.179, 0.498 | This Work |
| **2** | ***p*-CF3** | 496 | 17.40 | LT_50_ = 390 h, (1000 nit) | 0.167, 0.463 | This Work |
| **3** | **tBuCz-*m*-CF3** | 485 | 31.62 | LT_50_ = 1162 h, (1000 nit) | 0.175, 0.446 | This Work |
| **4** | **tBuCz-*p*-CF3** | 485 | 30.72 | LT_50_ = 1237 h, (1000 nit) | 0.166, 0.410 | This Work |
| 5 | Ir1D | 462 | 25.1 | LT_70_ = 355 h, (1000 nit) | 0.175, 0.280 | ^6^ |
| 6 | Ir4 | 474 | 17.8 | LT_70_ = 93.0 h, (500 nit) | - | ^7^ |
| 7 | MS2 | 475 | 30.1 | LT_50_ = 2203 h, (327 nit) | 0.16, 0.38 | ^8^ |
| 8 | 1 | 462 | 30.9 | LT_50_ = 5 h, (200 nit) | 0.14, 0.23 | ^9^ |
| 9 | Ir(dmp)_3_ | 462 | 8.5 | LT_50_ = 510 h, (1000 nit) | 0.14, 0.23 | ^10^ |
| 10 | Ir(dmp)_3_ | 462 | 10.4 | LT_90_ = 110 h, (800 nit) | 0.15, 0.20 | ^11^ |
| 11 | 1a | 470 | 18.1 | LT_50_ = 34.3 h, (400 nit) | 0.15, 0.35 | ^12^ |
| 12 | *f*-ct1a | 472 | 20.5 | LT_70_ = 99 h, (500 nit) | 0.13, 0.19 | ^13^ |
| 13 | Ir(dbi)_3_ | 474/495 | 14 | LT_70_ = 180 h, (500 nit) | 0.19, 0.44 | ^14^ |
| 14 | Ir(dbi)_3_ | 474/495 | 24.8 | LT_50_ = 140 h, (1000 nit) | 0.19, 0.41 | ^15^ |
| 15 | *f*-Ir(iprpmi)_3_ | 471 | 19.6 | LT_50_ = 122.6 h, (1000 nit) | 0.17, 0.40 | ^16^ |
| 16 | Ir(cb)_3_ | 467 | 21.0 | LT_50_ = 3130 h, (100 nit) | 0.14, 0.18 | ^17^ |
| 17 | Ir(cb)_3_ | 460 | 27.6 | LT_50_ = 10700 h, (100 nit) | 0.12, 0.13 | ^18^ |
| 18 | IrE | 462 | 23.4 | LT_95_ = 232 h, (500 nit) | - | ^19^ |
| 19 | Ir(dbi)_3_ | 474/495 | 4.91 | LT_70_ > 50 (1000 nit) | 0.20, 0.41 | ^20^ |
| 20 | Ir(dbi)_3_ | 474/495 | 6.83 | LT_70_ > 30 (1000 nit) | 0.20, 0.43 | ^20^ |
| 21 | Ir(ppy)_3_ | 516 | 24.2 | LT_50_ = 16281 (1000 nit) | 0.31, 0.62 | ^21^ |

**Table S12.** EQE-CIEy relationship of recently reported ν-DABNA-based hyper OLED devices with different sensitizers.

| **Samples** | **Sensitizer** | **V_on_ (V)** | **EQE_max_ (%)** | **λ_EL_ (nm)** | **CIE (x, y)** | **FWHM (nm)** | **LT (h)** | **Ref.** |
| --- | --- | --- | --- | --- | --- | --- | --- | --- |
| 1 | DOBNA-OAr | 3.4 | 34.4 | 469 | 0.12, 0.11 | 18 | - | ^22^ |
| 2 | DBA-BFICz | 3.1 | 38.8 | 473 | 0.12, 0.15 | 19 | - | ^23^ |
| 3 | HDT-1 | 3.0 | 27 | 470 | 0.15, 0.20 | 18 | LT_95_=18h (1000 cd m^-2^) Tandem | ^24^ |
| 4 | CN-Ir | 3.0 | 27.3 | 470 | 0.132, 0.162 | 20 | LT_50_=121h (1000 cd m^-2^) | ^25^ |
| 5 | PCzTRz | 3.0 | 33.5 | 473 | 0.12, 0.18 | 19 | LT_50_ =151h (1000 cd m^-2^) | ^26^ |
| 6 | PtON7-dtb | 3.0 | 32.2 | 473 | 0.111, 0.141 | 20 | LT_50_ =156.3h (1000 cd m^-2^) | ^27^ |
| 7 | TSF-dCz | - | 34.7 | 471 | 0.13, 0.15 | 19 | - | ^28^ |
| 8 | m-2-tBu | 3.5 | 22 | 472 | 0.120, 0.155 | 21 | - | ^29^ |
| 9 | Complex 5 | - | 23.4 | 469 | 0.13, 0.12 | 18 | LT_50_ =259h (1000 cd m^-2^) | ^30^ |
| 10 | Au-1 | 4.0 | 16.6 | 472 | 0.14, 0.18 | 23 | - | ^31^ |
| 11 | B3 | 4.0 | 26.17 | 473 | 0.116, 0.114 | - | - | ^32^ |
| 12 | DBA-DTMCz | 3.2 | 43.9 | 473 | 0.12, 0.16 | 21 | - | ^33^ |
| 13 | B-5-TMS | - | 33.4 | 470 | 0.119,0.123 | 17 | LT_50_=4552h (100 cd m^-2^) | ^34^ |
| 14 | Complex 6 | 3.4 | 15.76 | 472 | 0.13, 0.19 | - | - | ^35^ |
| 15 | *f*ct-1c | 3.1 | 35.5 | 472 | 0.119, 0.107 | 18 | LT_70_=95h (500 cd m^-2^) | ^13^ |
| 16 | D-5CzBN | - | 30.4 | 468 | 0.15, 0.22 | 22 | LT_80_=561h (1000 cd m^-2^) | ^36^ |
| 17 | tBuCz-dfppy-CN | 3.4 | 31.6 | 470 | 0.120, 0.136 | 18 | - | ^37^ |
| 18 | ACRSA | - | 30 | 476 | - | 17.7 | - | ^38^ |
| 19 | Ce-2 | 3.1 | 30.0 | 471 | 0.13, 0.14 | 20 | - | ^39^ |
| 20 | *f*-ct5mix | 3.6 | 32.0 | 470 | 0.127, 0.098 | 17 | - | ^40^ |
| 21 | tBuCz-*m*-CF3 | 2.75 | 29.50 | 470 | 0.141, 0.233 | 20 | LT_50_=302h (1000 cd m^-2^) | This work |
| 22 | tBuCz-*p*-CF3 | 2.80 | 29.78 | 470 | 0.140, 0.233 | 21 | LT_50_=318h (1000 cd m^-2^) | This work |

References

1 (M. J. Frisch, G. W. Trucks, H. B. Schlegel, G. E. Scuseria, M. A. Robb, J. R. Cheeseman, G. Scalmani, V. Barone, G. A. Petersson, H. Nakatsuji, X. Li, M. Caricato, A. V. Marenich, J. Bloino, B. G. Janesko, R. Gomperts, B. Mennucci, H. P. Hratchian, J. V. Ortiz, A. F. Izmaylov, J. L. Sonnenberg, D. Williams-Young, F. Ding, F. Lipparini, F. Egidi, J. Goings, B. Peng, A. Petrone, T. Henderson, D. Ranasinghe, V. G. Zakrzewski, J. Gao, N. Rega, G. Zheng, W. Liang, M. Hada, M. Ehara, K. Toyota, R. Fukuda, J. Hasegawa, M. Ishida, T. Nakajima, Y. Honda, O. Kitao, H. Nakai, T. Vreven, K. Throssell, J. A. Montgomery, Jr., J. E. Peralta, F. Ogliaro, M. J. Bearpark, J. J. Heyd, E. N. Brothers, K. N. Kudin, V. N. Staroverov, T. A. Keith, R. Kobayashi, J. Normand, K. Raghavachari, A. P. Rendell, J. C. Burant, S. S. Iyengar, J. Tomasi, M. Cossi, J. M. Millam, M. Klene, C. Adamo, R. Cammi, J. W. Ochterski, R. L. Martin, K. Morokuma, O. Farkas, J. B. Foresman, and D. J. Fox, Gaussian, Inc., Wallingford CT, 2019).

2 Hay, P. J. & Wadt, W. R. Ab initio effective core potentials for molecular calculations. Potentials for K to Au including the outermost core orbitals. *J. Chem. Phys.* **82**, 299-310 (1985). <https://doi.org/10.1063/1.448975>

3 Hay, P. J. & Wadt, W. R. Ab initio effective core potentials for molecular calculations. Potentials for the transition metal atoms Sc to Hg. *J. Chem. Phys.* **82**, 270-283 (1985). <https://doi.org/10.1063/1.448799>

4 Tapia, O. Solvent effect theories: Quantum and classical formalisms and their applications in chemistry and biochemistry. *Journal of Mathematical Chemistry* **10**, 139-181 (1992). <https://doi.org/10.1007/BF01169173>

5 Tomasi, J. & Persico, M. Molecular Interactions in Solution: An Overview of Methods Based on Continuous Distributions of the Solvent. *Chem. Rev.* **94**, 2027-2094 (1994). <https://doi.org/10.1021/cr00031a013>

6 Bae, H. J. *et al.* Protecting Benzylic C-H Bonds by Deuteration Doubles the Operational Lifetime of Deep‐Blue Ir‐Phenylimidazole Dopants in Phosphorescent OLEDs. *Advanced Optical Materials* **9**, 2100630 (2021). <https://doi.org/https://doi.org/10.1002/adom.202100630>

7 Kim, S. *et al.* Degradation of blue-phosphorescent organic light-emitting devices involves exciton-induced generation of polaron pair within emitting layers. *Nature Communications* **9**, 1211 (2018). <https://doi.org/10.1038/s41467-018-03602-4>

8 Sarma, M. *et al.* Anomalously Long-Lasting Blue PhOLED Featuring Phenyl-Pyrimidine Cyclometalated Iridium Emitter. *Chem* **3**, 461-476 (2017). <https://doi.org/https://doi.org/10.1016/j.chempr.2017.08.001>

9 Zaen, R., Park, K. M., Lee, K. H., Lee, J. Y. & Kang, Y. Blue Phosphorescent Ir(III) Complexes Achieved with Over 30% External Quantum Efficiency. *Advanced Optical Materials* **7**, 1901387 (2019). <https://doi.org/https://doi.org/10.1002/adom.201901387>

10 Zhang, Y., Lee, J. & Forrest, S. R. Tenfold increase in the lifetime of blue phosphorescent organic light-emitting diodes. *Nature Communications* **5**, 5008 (2014). <https://doi.org/10.1038/ncomms6008>

11 Zhao, H., Arneson, C. E., Fan, D. & Forrest, S. R. Stable blue phosphorescent organic LEDs that use polariton-enhanced Purcell effects. *Nature* **626**, 300-305 (2024). <https://doi.org/10.1038/s41586-023-06976-8>

12 Yi, S. *et al.* Stable Blue Phosphorescence Iridium(III) Cyclometalated Complexes Prompted by Intramolecular Hydrogen Bond in Ancillary Ligand. *Inorganic Chemistry* **55**, 3324-3331 (2016). <https://doi.org/10.1021/acs.inorgchem.5b02511>

13 Yan, J. *et al.* Electroluminescence and hyperphosphorescence from stable blue Ir(III) carbene complexes with suppressed efficiency roll-off. *Nature Communications* **14**, 6419 (2023). <https://doi.org/10.1038/s41467-023-42090-z>

14 Seo, J.-A. *et al.* Long lifetime blue phosphorescent organic light-emitting diodes with an exciton blocking layer. *Journal of Materials Chemistry C* **3**, 4640-4645 (2015). <https://doi.org/10.1039/c5tc00640f>

15 Yang, J. W. & Lee, J. Y. Correlation of the molecular structure of host materials with lifetime and efficiency of blue phosphorescent organic light-emitting diodes. *Phys. Chem. Chem. Phys.* **17**, 24468-24474 (2015). <https://doi.org/10.1039/C5CP03469H>

16 Zhang, L. *et al.* Highly Efficient Blue Phosphorescent Organic Light-Emitting Diodes Employing a Host Material with Small Bandgap. *ACS Applied Materials & Interfaces* **8**, 16186-16191 (2016). <https://doi.org/10.1021/acsami.6b01304>

17 Yun, J. H. *et al.* Thermally activated delayed fluorescence type exciplex host for long lifetime in deep blue phosphorescent organic light-emitting diodes. *Chemical Engineering Journal* **417**, 128086 (2021). <https://doi.org/10.1016/j.cej.2020.128086>

18 Jung, M., Lee, K. H., Lee, J. Y. & Kim, T. A bipolar host based high triplet energy electroplex for an over 10 000 h lifetime in pure blue phosphorescent organic light-emitting diodes. *Materials Horizons* **7**, 559-565 (2020). <https://doi.org/10.1039/C9MH01268K>

19 Ihn, S.-G. *et al.* Cohosts with efficient host-to-emitter energy transfer for stable blue phosphorescent organic light-emitting diodes. *Journal of Materials Chemistry C* **9**, 17412-17418 (2021). <https://doi.org/10.1039/d1tc04680b>

20 Kim, H. O. *et al.* COMPARISON OF TIMING OF NON-CULPRIT LESION PERCUTANEOUS CORONARY INTERVENTION IN MYOCARDIAL INFARCTION PATIENTS. *Journal of the American College of Cardiology* **69**, 130 (2017). <https://doi.org/https://doi.org/10.1016/S0735-1097(17)33519-2>

21 Kawano, T. *et al.* Tetrapyridine/triphenyltriazine-conjugated electron transporters for low-power-consumption, high-stability phosphorescent OLEDs. *Journal of Materials Chemistry C* **11**, 4129-4135 (2023). <https://doi.org/10.1039/D2TC04647D>

22 Kondo, Y. *et al.* Narrowband deep-blue organic light-emitting diode featuring an organoboron-based emitter. *Nature Photonics* **13**, 678-682 (2019). <https://doi.org/10.1038/s41566-019-0476-5>

23 Braveenth, R. *et al.* Achieving Narrow FWHM and High EQE Over 38% in Blue OLEDs Using Rigid Heteroatom-Based Deep Blue TADF Sensitized Host. *Advanced Functional Materials* **31**, 2105805 (2021). <https://doi.org/https://doi.org/10.1002/adfm.202105805>

24 Chan, C.-Y. *et al.* Stable pure-blue hyperfluorescence organic light-emitting diodes with high-efficiency and narrow emission. *Nature Photonics* **15**, 203-207 (2021). <https://doi.org/10.1038/s41566-020-00745-z>

25 Chung, W. J. *et al.* Over 30 000 h Device Lifetime in Deep Blue Organic Light-Emitting Diodes with y Color Coordinate of 0.086 and Current Efficiency of 37.0 cd A−1. *Advanced Optical Materials* **9**, 2100203 (2021). <https://doi.org/https://doi.org/10.1002/adom.202100203>

26 Jeon, S. O. *et al.* High-efficiency, long-lifetime deep-blue organic light-emitting diodes. *Nature Photonics* **15**, 208-215 (2021). <https://doi.org/10.1038/s41566-021-00763-5>

27 Nam, S. *et al.* Improved Efficiency and Lifetime of Deep-Blue Hyperfluorescent Organic Light-Emitting Diode using Pt(II) Complex as Phosphorescent Sensitizer. *Advanced Science* **8**, 2100586 (2021). <https://doi.org/https://doi.org/10.1002/advs.202100586>

28 Huang, T., Wang, Q., Meng, G., Duan, L. & Zhang, D. Accelerating Radiative Decay in Blue Through-Space Charge Transfer Emitters by Minimizing the Face-to-Face Donor–Acceptor Distances. *Angewandte Chemie International Edition* **61**, e202200059 (2022). <https://doi.org/https://doi.org/10.1002/anie.202200059>

29 Jin, J. *et al.* Iridium(III) Phosphors–Bearing Functional 9-Phenyl-7,9-dihydro-8H-purin-8-ylidene Chelates and Blue Hyperphosphorescent OLED Devices. *Advanced Photonics Research* **3**, 2100381 (2022). <https://doi.org/https://doi.org/10.1002/adpr.202100381>

30 Lo, K.-W., Tong, G. S. M., Cheng, G., Low, K.-H. & Che, C.-M. Dinuclear PtII Complexes with Strong Blue Phosphorescence for Operationally Stable Organic Light-Emitting Diodes with EQE up to 23 % at 1000 cd m^−2^. *Angewandte Chemie International Edition* **61**, e202115515 (2022). <https://doi.org/https://doi.org/10.1002/anie.202115515>

31 Zhou, D., Wu, S., Cheng, G. & Che, C.-M. A gold(iii)–TADF emitter as a sensitizer for high-color-purity and efficient deep-blue solution-processed OLEDs. *Journal of Materials Chemistry C* **10**, 4590-4596 (2022). <https://doi.org/10.1039/D1TC05487B>

32 Zhu, Z.-L. *et al.* Efficient Blue Electrophosphorescence and Hyperphosphorescence Generated by Bis-tridentate Iridium(III) Complexes. *Inorganic Chemistry* **61**, 8898-8908 (2022). <https://doi.org/10.1021/acs.inorgchem.2c01026>

33 Lee, H. *et al.* Efficient pure blue hyperfluorescence devices utilizing quadrupolar donor-acceptor-donor type of thermally activated delayed fluorescence sensitizers. *Nature Communications* **14**, 419 (2023). <https://doi.org/10.1038/s41467-023-35926-1>

34 Wu, C. *et al.* New [3+2+1] Iridium Complexes as Effective Phosphorescent Sensitizers for Efficient Narrowband Saturated-Blue Hyper-OLEDs. *Advanced Science* **10**, e2301112 (2023). <https://doi.org/10.1002/advs.202301112>

35 Wu, C. *et al.* Blue Iridium (III) Phosphorescent OLEDs with High Brightness Over 10000 cd m^−2^ and Ultralow Efficiency Roll-Off. *Advanced Optical Materials* **11**, 2201998 (2023). <https://doi.org/https://doi.org/10.1002/adom.202201998>

36 Huang, T. *et al.* Enhancing the efficiency and stability of blue thermally activated delayed fluorescence emitters by perdeuteration. *Nature Photonics* (2024). <https://doi.org/10.1038/s41566-024-01379-1>

37 Shi, K. *et al.* Enhanced Emitting Dipole Orientation Based on Asymmetric Iridium(III) Complexes for Efficient Saturated-Blue Phosphorescent OLEDs. *Advanced Science* **n/a**, 2402349 (2024). <https://doi.org/https://doi.org/10.1002/advs.202402349>

38 Stavrou, K., Franca, L. G., Danos, A. & Monkman, A. P. Key requirements for ultraefficient sensitization in hyperfluorescence organic light-emitting diodes. *Nature Photonics* (2024). <https://doi.org/10.1038/s41566-024-01395-1>

39 Sun, Y.-F. *et al.* Efficient Deep-Blue Organic Light-Emitting Diodes Employing Doublet Sensitization. *Advanced Materials* **n/a**, 2408118 (2024). <https://doi.org/https://doi.org/10.1002/adma.202408118>

40 Yan, J. *et al.* Structural Engineering of Iridium(III) Phosphors with Imidazo[4,5-b]pyrazin-2-ylidene Cyclometalates for Efficient Blue Electroluminescence. *Small Methods*, 2301555 (2024). <https://doi.org/https://doi.org/10.1002/smtd.202301555>
